# Dummy Atoms in Alchemical Free Energy Calculations

**DOI:** 10.1021/acs.jctc.0c01328

**Published:** 2021-06-14

**Authors:** Markus Fleck, Marcus Wieder, Stefan Boresch

**Affiliations:** † Faculty of Chemistry, Department of Computational Biological Chemistry, University of Vienna, Währingerstraße 17, A-1090 Vienna, Austria; ‡ Department of Pharmaceutical Sciences, Faculty of Life Sciences, University of Vienna, Althanstraße 14, 1090 Vienna, Austria

## Abstract

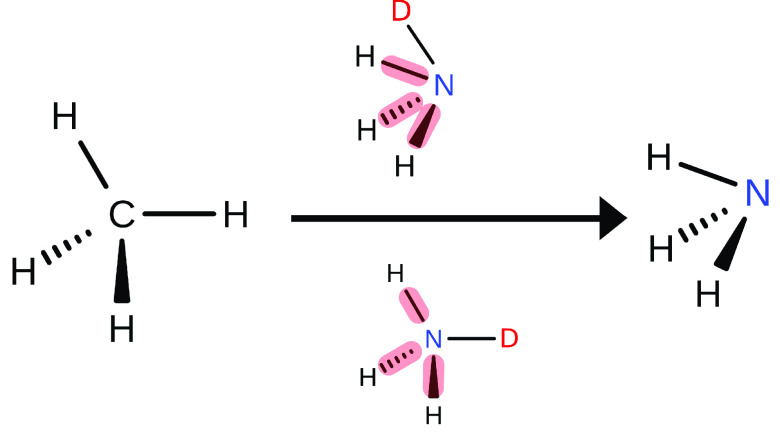

In calculations of relative free energy differences, the number of atoms of the initial and final states is rarely the same. This necessitates the introduction of dummy atoms. These placeholders interact with the physical system only by bonded energy terms. We investigate the conditions necessary so that the presence of dummy atoms does not influence the result of a relative free energy calculation. On the one hand, one has to ensure that dummy atoms only give a multiplicative contribution to the partition function so that their contribution cancels from double-free energy differences. On the other hand, the bonded terms used to attach a dummy atom (or group of dummy atoms) to the physical system have to maintain it in a well-defined position and orientation relative to the physical system. A detailed theoretical analysis of both aspects is provided, illustrated by 24 calculations of relative solvation free energy differences, for which all four legs of the underlying thermodynamic cycle were computed. Cycle closure (or lack thereof) was used as a sensitive indicator to probing the effects of dummy atom treatment on the resulting free energy differences. We find that a naive (but often practiced) treatment of dummy atoms results in errors of up to *k*_*BT*_ when calculating the relative solvation free energy difference between two small solutes, such as methane and ammonia. While our analysis focuses on the so-called single topology approach to set up alchemical transformations, similar considerations apply to dual topology, at least many widely used variants thereof.

## 1 Introduction

So-called alchemical free energy simulations (FES) have become a standard tool of computational chemists, both in academia as well as in pharmaceutical research in industry, in particular for lead optimization.^1−3^ While the methodology can be used to compute “absolute” free energies (solvation free energy differences, binding free energy differences),^4−6^ in many cases knowledge of relative free energy differences, for example, the binding free energy difference between two ligands, is sufficient.^1−3^ These successful applications and the beginning widespread use by nonexperts make it important to keep an eye on remaining methodological challenges and pitfalls.

**Figure 1:**
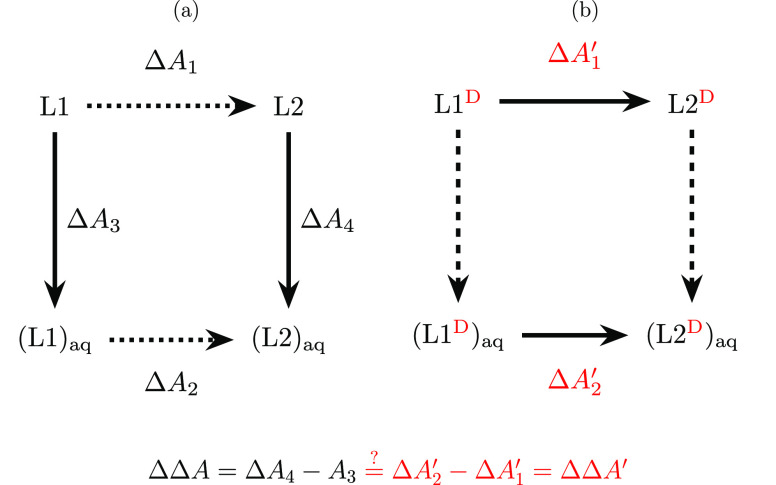
(a) *Idealized* thermodynamic cycle used to compute the relative solvation free energy difference between two solutes, L1 and L2. Since the free energy is a state function, *ΔΔA* = *ΔA*_4_ – *ΔA*_3_ = *ΔA*_2_ – *ΔA*_1_.^7^ The horizontal, dashed arrows indicate the idealized alchemical paths in the absence of dummy atoms. (b) Thermodynamic cycle *required in practice* whenever the number of atoms in L1 and L2 is not the same. The presence of dummy atoms is indicated by the superscript D. For example, L1^D^ denotes ligand L1 including any dummy atoms attached to make the number of atoms at the two end states identical. Thus, along the horizontal, alchemical paths one computes *ΔΔA*′ = *A*_2_^′^ – *A*_1_^′^, rather than *ΔΔA* = *A*_2_ – *A*_1_.

Calculations of relative free energy differences rely on a thermodynamic cycle as shown in Figure 1(a).^7^ Rather than computing the double free energy difference of interest according to *ΔΔA* = *ΔA*_4_ – *ΔA*_3_, as would be done in an experiment, it is obtained along the “alchemical” paths, *ΔΔA* = *ΔA*_2_ – *ΔA*_1_. Thus, one needs to transform only a small part of the system, i.e., such as changing a functional group into another. In most practical applications, however, the number of atoms in the initial and final chemical moiety (the parts which are different in, for example, two ligands of interest) is not the same. Since the number of particles in simulations in the canonical ensemble must not change, one needs to add “placeholders” at one or both end states to formally maintain the overall number of particles. Especially in the so-called single topology paradigm,^8^ these placeholders are usually referred to as *dummy atoms*. This is made explicit in Figure 1(b), where the presence of dummy atoms is indicated by the superscripts D. In most practical calculations, one really computes *ΔΔA*^′^ = *A*_2_^′^ – *A*_1_^′^ as shown in Figure 1(b), relying on that *ΔΔA*′ equals *ΔΔA* from the idealized cycle Figure 1(a).

Dummy atoms do not participate in any nonbonded interactions. However, they must remain connected to the physical system, either through bonded energy terms or suitable restraints.^9−11^ Typically, some or all of the bonded force field terms present for the native atom are kept for the corresponding dummy atom. If dummy atoms are attached to the physical part of the system through bonded energy terms, the question arises whether they alter the properties of the physical system. In dual topology approaches,^8,12^ in which both states are present simultaneously, the problem seems avoided, but at each of the endpoints, one of the groups has no nonbonded interactions with the remainder of the system, yet remains connected to the physical system. Thus, it is justified to consider dummy atoms also in connection with dual topology approaches.^12,13^ In the literature, dummy atoms have been discussed mostly in connection with the contribution from changes in bonded energy terms to free energy differences.^9−11,13,14^ However, we are not aware of practical recipes how to best and/or correctly handle dummy atoms in alchemical transformations. The goal of this article is to fill this gap, by providing both a theoretical analysis, as well as practical guidelines. In doing so, we focus on the single topology paradigm, but we outline how our findings are relevant to (some forms of) dual topology and single/dual topology hybrid approaches.

The equivalence of the idealized thermodynamic cycle Figure 1(a) and the situation found in practice (Figure 1(b)) is guaranteed if and only if dummy atoms add the same factor to each of the two free energy differences. Phrased differently, their contribution to the partition function must be multiplicative and, hence, separable. If a dummy atom is attached to physical atoms by three nonredundant bonded energy terms (expressed in terms of three suitable internal coordinates), such a factorization is indeed possible.^15^ This is, however, *not* common practice. While rarely stated explicitly, it seems customary to turn off the nonbonded interactions with a dummy atom but to maintain *all* bonded energy terms, which this (dummy) atom had at the corresponding physical end state. In modern force fields, all possible valence and dihedral angles are generated based on connectivity, resulting in many more than three bonded terms per atom. Furthermore, using just three bonded terms per dummy atom may result in unstable simulations and/or failure of the free energy methods to converge. Such erratic behavior of dummy atoms, which we refer to as “flapping”, has been documented (see ref (13) and the SI of ref (16)), but we are not aware of any systematic analysis of such phenomena.

To summarize, in this study, we want to address systematically two questions: (1) Do dummy atoms influence the real atoms, or, phrased alternatively, is their contribution to a single free energy difference additive so that it cancels from the thermodynamic cycle of interest? (2) How many and/or what type of bonded terms are needed to keep dummy atoms in a well-defined average geometry, as well as the whole dummy group in a “meaningful” orientation relative to the physical part of the system? Both aspects are addressed first theoretically (Sections 2.1 and 2.2), followed by a systematic outline and set of rules on how to handle dummy atoms (Section 2.3). Some aspects concerning setting up alchemical FES in the dual topology paradigm are discussed in Section 2.4. The theoretical considerations are accompanied by results from relative solvation free energy calculations for 12 pairs of molecules, many of which were carried out in more than one way. To probe the effects of the handling of dummy atoms on the overall results, we also computed absolute solvation free energies. Thus, we could explicitly calculate *ΔΔA* = *ΔA*_4_ – *ΔA*_3_ in the notation of Figure 1(a). The result(s) along the alchemical route, *ΔΔA*^′^ = *ΔA*_2_^′^ – *ΔA*_1_^′^ (cf. Figure 1(b)) was (were) then compared to this reference value. On the basis of these calculations, we can discern unambiguously whether the presence of dummy atoms (or rather their treatment) leads to systematic errors.

## 2 Theory

### 2.1 Dummy Atom Contributions to the Partition Function

#### 2.1.1 General Considerations

The potential energy function of a system *L*^*D*^ containing dummy atoms (D) can be schematically written as
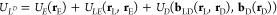
1In eq 1, the term *U*_*LE*_ encompasses all bonded and nonbonded interactions within the physical molecule L and the nonbonded interactions between L and the environment E (e.g., aqueous solution, receptor to which L is bound, etc.). *U*_*E*_ denotes all interactions in E and is irrelevant for the following. The last term, *U*_*D*_, comprises all interactions in which dummy atoms participate. By employing the notation **b**_LD_(**r**_L_,**r**_D_) and **b**_D_(**r**_D_), we emphasize that dummy atoms interact only through bonded terms. Additivity of the potential energy does *not* result in a factorization of the partition function (i.e., in general, *Z*(*L*^*D*^) ≠ *Z*(*L*) × *Z*(*D*)),^17,18^ which is required for the identity *ΔA*_2_ – *ΔA*_1_ = *ΔA*_2_^′^ – *ΔA*_1_^′^ (cf. Introduction and Figure 1) to hold. However, using *suitable* internal coordinates for the position and orientation of the dummy atoms ({**b**_LD_^′^,**b**_D_^′^}) and adapting steps outlined, for example, in refs (15 and 19−21), one can indeed obtain the partition function in the desired form
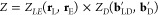
2The derivation of eq 2 relies crucially on using exactly *three*, *nonredundant* degrees of freedom (three bonded energy terms) per dummy atom. Additional force field terms acting on a dummy atom, the situation found in practice, are referred to as *redundant*.^15^ The prerequisites and steps leading to eq 2 are outlined in more detail in Section 1.1 of the SI.

When investigating how the presence of redundant energy terms affects the separability of the partition function, one has to distinguish two cases. Any redundant degrees of freedom (bonded energy terms), which depend only on positions (coordinates) of dummy atoms, are of no concern; their contribution to the partition function can always be factored. In fact, within large groups of dummy atoms, all bonded interactions should be kept to maintain its structural integrity. By contrast, care is required in the “junction region”, i.e., for redundant bonded terms, which involve coordinates of physical *as well as* of dummy atoms. It is these cases that we analyze in the following subsection.

#### 2.1.2 Coupling between Dummy and Physical Atoms

The prerequisite for eq 2 is that each dummy atom in direct 1–2 or 1–3/1–4 proximity to atoms of the physical system L is connected by three nonredundant energy terms in suitable internal coordinates (distances, angles, dihedral angles). As mentioned in the Introduction, however, in modern force fields, a bonded energy term is assigned to each valence and dihedral angle formed by the covalent bonds; this means that there are usually more than three bonded energy terms acting on an atom. We, therefore, need to explore the effect of keeping additional bonded terms in the junction region.

##### Six-Angle Constraints

**Figure 2:**
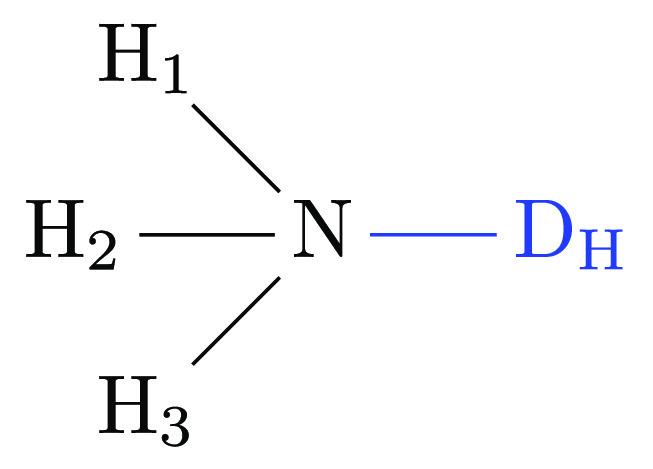
Ammonia endpoint with dummy atom D_*H*_ of the alchemical transformation methane CH_4_ to NH_3_.

Consider the mutation of methane to ammonia; the ammonia endpoint with the dummy atom attached is shown in Figure 2. As there are five atoms, there are 3 × 5 – 6 = 9 nonredundant degrees of freedom, four of which are needed for the four bonds, leaving five nonredundant valence angle degrees of freedom. In methane, however, there are six H–C–H bond angles. Thus, what is the effect of keeping the redundant sixth angle, present at the methane endpoint, for ammonia with the additional dummy atom attached? The six angles share a common central atom; this leads to the following constraint (written in generic form):^22^
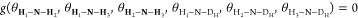
3The use of constraints to qualitatively understand the influence of redundant degrees of freedom is exemplified in Section 1.2 of the SI, using a two-dimensional model system.

Any of the three angles θ_H_i_–N–D_H__ can be picked as the redundant angle term. Equation 3 makes clear that internal degrees of freedom (i.e., bonded energy terms) involving the dummy atom couple with bonded terms involving only the physical ammonia end state (marked in bold). Thus, when keeping the sixth angle (maintaining the redundant bond angle term), the separation of the partition function eq 2 is no longer possible; the θ_H_i_–N–D_H__ and θ_H_i_–N–H_j__ angles are coupled.

##### Coupling between Valence and Dihedral Angles

**Figure 3:**
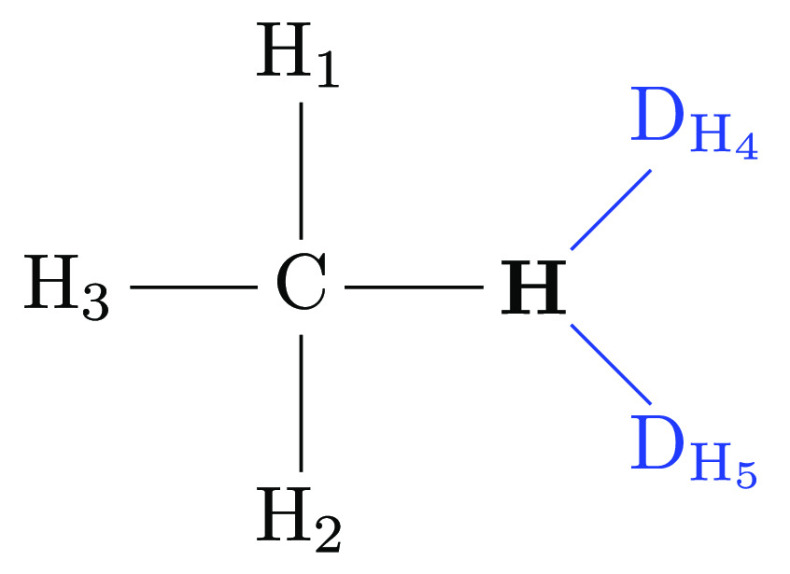
Methane endpoint of the alchemical transformation from methylamine to methane. Hydrogen **H** corresponds to the amine nitrogen. The two dummy atoms, corresponding to the amine hydrogens, are shown explicitly.

**Figure 4:**
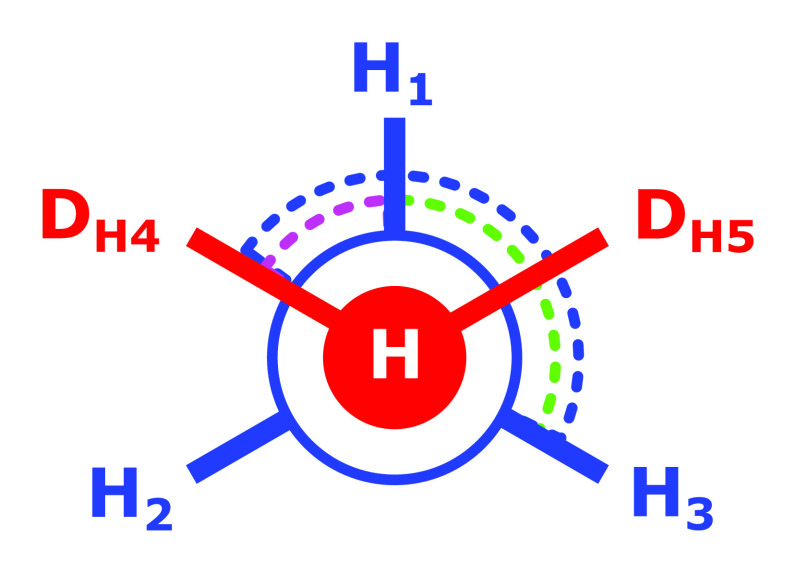
Newman projection-like depiction of two dihedrals sharing three atoms, using the methylamine to methane transformation as an example (see Figure 3 for the atom labeling). Atoms/bonds in the foreground are drawn in red and atoms/bonds in the background in blue. The methyl carbon C is hidden behind the red circle for H. The two dihedrals φ_D_H4_–H–C–H_1__ and φ_D_H4_–H–C–H_3__ under consideration are indicated in purple and blue, respectively. The difference θ_H_1_–C–H_3__^′^ between the two dihedrals is colored in green; this is also the angle between the projection of the bonds H_1_–C and H_3_–C into the plane perpendicular on the bond H–C.

An additional class of constraints arises from interactions between valence and dihedral angles. We discuss two representative cases using as an example the alchemical transformation from methylamine to methane. The methane end state including dummy atoms is shown in Figure 3; the hydrogen corresponding to a nitrogen at the methylamine end state is highlighted in bold. Here, we concentrate on the effects, if any, of adding redundant dihedral angle terms on top of the three nonredundant degrees of freedom (i.e., bonded energy terms) one can use for each of the dummy atoms. We start by considering dummy atom D_H_4__. It could be connected through the bond *r*_D_H4_–H_ and, for example, the angle θ_D_H4_–H–C_ plus the dihedral φ_D_H4_–H–C–H_1__ to the physical methane molecule (the atom labeling is shown in Figure 3). What about keeping additional dihedrals present at the methane endpoint? We first consider the case when two dihedrals share three common atoms. An example for such a situation would be adding (or keeping) the dihedral angle energy term φ_D_H4_–H–C–H_3__ in addition to φ_D_H4_–H–C–H_1__ at the methane endpoint. A dihedral angle φ_1–2–3–4_ describes the relative orientation between the two outer bonds *r*_1–2_ and *r*_3–4_ with respect to a plane perpendicular to the middle bond *r*_2–3_. In other words, it is the angle between the two outer bonds when projected to a plane perpendicular to the central bond. This projection for the φ_D_H4_–H–C–H_1__ (in purple) and φ_D_H4_–H–C–H_3__ dihedrals (in blue) in the methylamine to methane transformation is depicted in Figure 4. The outer bond D_H_4__–H shared by the two dihedrals acts as the common anchor. Thus, the difference between the two dihedral angles is the angle θ_H_1_–C–H_3__^′^ formed by the projections of the bonds C–H_1_ and C–H_3_ into the plane perpendicular to C–H. But this projected angle (shown in green in Figure 4)
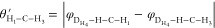
4also depends on the bond angles θ_H_1_–C–H_3__, θ_H–C–H_1__, and θ_H–C–H_3__. We, thus, can formulate the generic constraint equation if the additional dihedral angle term in φ_D_H4_–H–C–H_3__ is present
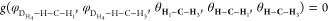
5Equation 5 involves the angle terms in θ_H_1_–C–H_3__, θ_H–C–H_1__, and θ_H–C–H_3__ of the physical methane end state (marked in bold). Thus, the constraint resulting from two dihedrals to a dummy atom in which three atoms are shared prevents the factorization of the partition function eq 2.

Another type of coupling between valence and dihedral angles arises if two dihedrals share the same central bond. In the methylamine–methane transformation this is, for example, the case when one adds/keeps the dihedral angle term in φ_D_H5_–H–C–H_3__, in addition to the term in φ_D_H4_–H–C–H_1__. In contrast to the previous case, there is no common anchor bond. Nevertheless, the two dihedral terms influence the angles of the projections of the bond pairs D_H_4__–H and D_H_5__–H, as well as C–H_1_ and C–H_3_ on the plane perpendicular to the central bond C–H. As before, each of these two projection angles also depends on three regular angle terms. For the specific case, this leads to the constraint
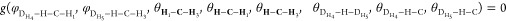
6Again, three physical valence angle terms highlighted in bold appear in the constraint equation. Thus, the strict separability of the partition function eq 2 is again lost.

The two cases involving both valence and dihedral angles arise fairly frequently in practice. To distinguish between the two forms of dihedral constraints, we refer to the first (three common atoms) as “single-anchored” and to the latter (common central bond) as “dual-anchored”.

##### Urey–Bradley Terms

Some force fields, such as the CHARMM family of force fields, augment selected angle bending terms by a Urey–Bradley term, a harmonic term in the 1–3 distance (see, for example, ref (23) for the rationale). It effectively introduces a cycle for the three atoms which form the angle. Hence, if one or more of these atoms are transformed into dummy atoms and the Urey–Bradley term is maintained, constraints may arise affecting the physical part of the molecule. One can easily convince oneself that the additional Urey–Bradley term of a bond angle needs to be deleted *whenever* the angle is made up from two physical and one dummy atoms. As an example, consider mutating a methyl group CH_3_ into a hydroxyl group OHD_2_, in which two of the methyl hydrogens have become dummy atoms. Let us assume that for the bond angle H–O–D a Urey–Bradley term is kept (O and H denote the atoms of the physical hydroxyl group). In the presence of the Urey–Bradley term *r*_H–D_, the following constraint applies
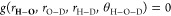
7As it involves a physical degree of freedom (*r*_H–O_), it affects the separability of the partition function eq 2.

##### Coupled Three Angles

**Figure 5:**
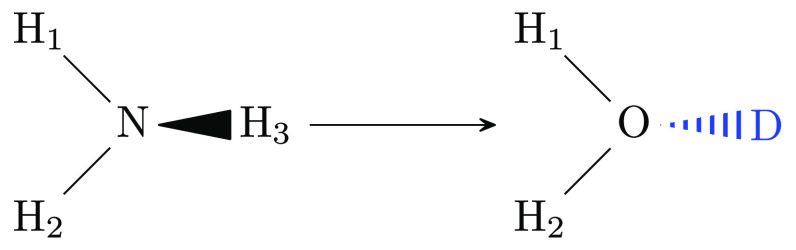
Alchemical transformation of ammonia into water.

Finally, unintended coupling between degrees of freedom of dummy atoms and physical atoms also arises when there are three atoms bound to a central atom, and one of these is transformed into a dummy atom. Note that in this case the dummy atom is connected to the physical system by only three bonded terms; i.e., the requirement of three nonredundant degrees of freedom is fulfilled. In addition, for such constellations, flapping can occur; this aspect is analyzed further in Section 2.2. As the simplest possible case, consider the alchemical transformation of ammonia to water shown in Figure 5.

In this particular case, one *has* to employ the bond *r*_O–D_ and the two angles θ_H_1_–O–D_ and θ_H_2_–O–D_ to maintain a well-defined position of the dummy atom relative to water. However, the choice of one bond stretching and two angle bending terms is appealing whenever a dummy atom has to be attached to a branched configuration. Although we are using only three nonredundant degrees of freedom, there is still coupling between the two angles involving the dummy atom and the physical bond angle θ_H_1_–O–H_2__ of the water molecule. Specifically, the following constraint applies to the physical bond angle
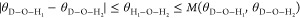
8where we use the shorthand
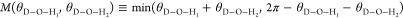
A full analysis of the situation is presented in Section 2.1 of the SI. The central result is that the effect of constraint eq 8 can be removed for all practical purposes by choosing θ_D–O–H_1__ = θ_D–O–H_2__ = π/2. In this case, the separability of the partition function (eq 2) is regained. In other words, whenever one anchors a dummy atom by one bond and two angles with respect to a physical molecule, one should set the equilibrium values of the two angles involving the dummy atom to 90°. As discussed shortly, this choice for the bond angles involving the dummy atom also effectively prevents flapping. Changing (an) angle(s) to 90° is a drastic change in molecular geometry. It may reduce phase–space overlap and necessitate additional λ-states. That being said, we did not encounter such difficulties in the test cases presented below.

### 2.2 Maintaining Structural Integrity of Dummy Atom Groups

So far, the focus was on cleanly factorizing the contributions from dummy atoms to the partition function. In line with earlier recommendations,^9−11,13,14^ this can be achieved by connecting dummy atoms to physical atoms through exactly three nonredundant bonded energy terms, although care is needed when using one bond and two bond angles. However, if held by only three nonredundant energy terms, a dummy atom (or a dummy atom group) may adopt poorly defined positions and/or orientations with respect to the physical molecule it is attached to. Such behavior occurs when the three internal coordinates and corresponding bonded energy terms used to position the dummy atom have more than one solution in terms of Cartesian coordinates, which, as we show below, can happen for a number of reasons. We use the term *flapping* to refer to such situations; descriptions of such phenomena can be found, for example, in ref (13) and the SI of ref (16).

#### Multiple Energy Minima

**Figure 6:**
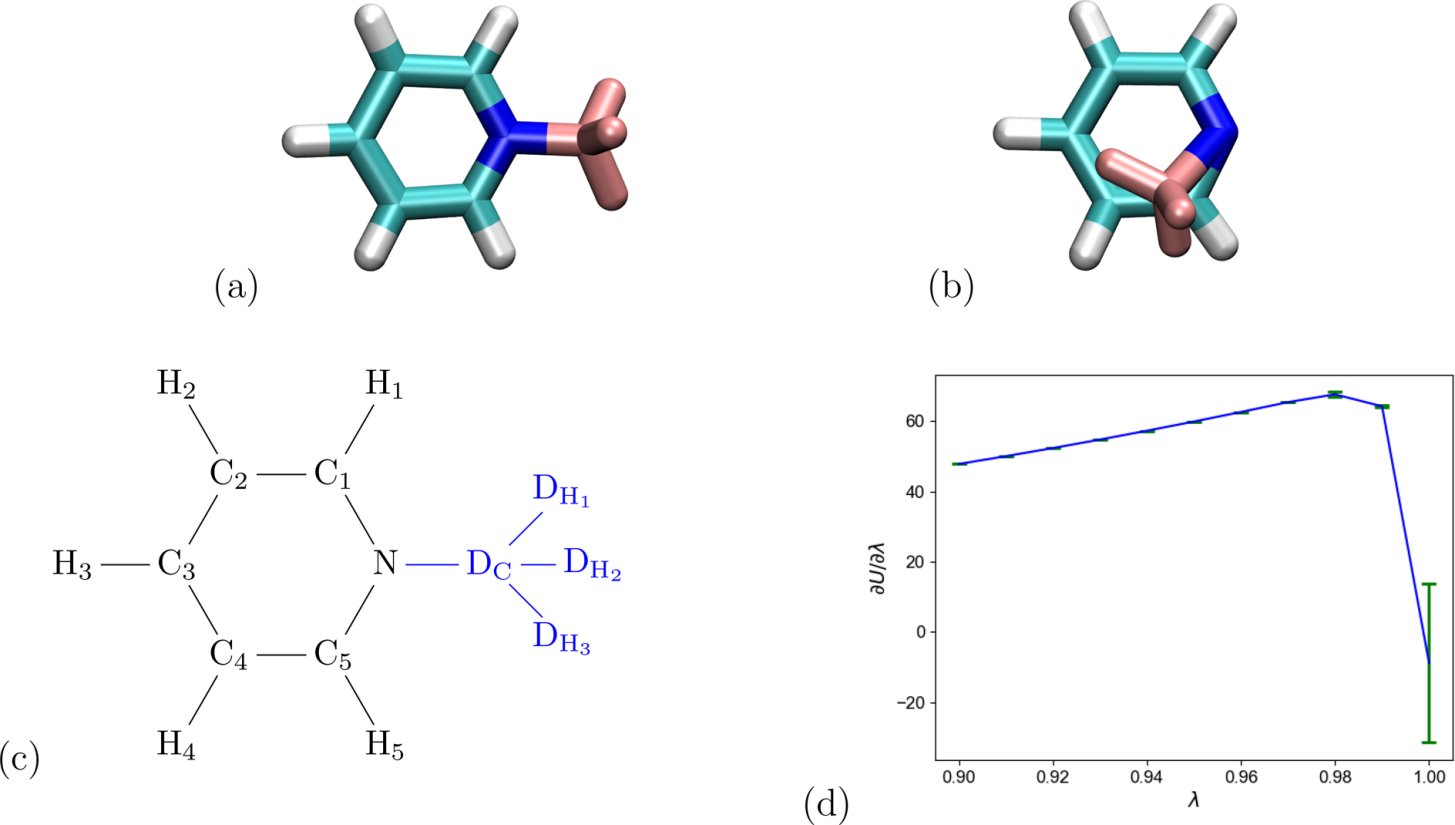
Alchemical transformation of toluene to pyridine. Two possible geometries encountered for the pyridine end state with dummy atoms (in pink). (a) Dummy methyl group in “proper” (toluene-like) geometry. (b) Alternative geometry resulting from the use of a periodic dihedral angle term (see main text for details). (c) Schematic sketch of pyridine end state with atom labels. (d) ⟨∂*U*/∂λ⟩_λ_ in kcal/mol near the pyridine end state, including error bars.

First, one of the nonredundant energy terms used can have multiple energy minima. For example, dihedral angle potentials are usually periodic, and this can cause a dummy atom (or dummy group) to adopt multiple positions in unexpected ways. To illustrate how this can lead to flapping, we turn to the alchemical transformation of toluene to pyridine (Figure 6). Following the best practice with respect to separability of the partition function, we attach dummy atom D_C_ to pyridine using the bond stretching term D_C_–N, the angle bending term D_C_–N–C_1_, and the dihedral D_C_–N–C_1_–C_2_ (Figure 6c depicts the atom labels). The force field parameters for these terms are taken from the corresponding energy terms in toluene. In this specific instance, the dihedral angle term in the CHARMM CGenFF force field^24−26^ has 2-fold periodicity. In toluene, the geometry of the methyl group with respect to the ring is enforced through the combination of several bonded terms, the most important being the angle D_C_–N–C_5_. At the pyridine endpoint, only the three bonded terms listed above are active. In this “reduced” force field, the two geometries shown in Figure 6a and b are both valid, and during a simulation at the pyridine end state (λ = 1), both configurations occur with equal probability. Figure 6d shows the effect of this behavior on the key quantity of thermodynamic integration, ⟨∂*U*/∂λ⟩_λ_ near λ = 1. At the pyridine end state, ⟨∂*U*/∂λ⟩_λ_ becomes extremely noisy, with error bars of ±15 kcal/mol. Further, the overall shape of the integrand changes drastically; from a smooth, almost linear behavior up to λ = 0.98, there is a sharp drop by almost 60 kcal/mol. Since the free energy difference is based on the numerical integration of these data, it is doubtful whether a converged result can be obtained. The two obvious solutions in such cases are to apply a harmonic potential to the dihedral angle or to use a dihedral potential with a periodicity of one. In the latter case, it may be necessary to simultaneously increase the force constant.

#### Essential Redundant Energy Terms

**Figure 7:**
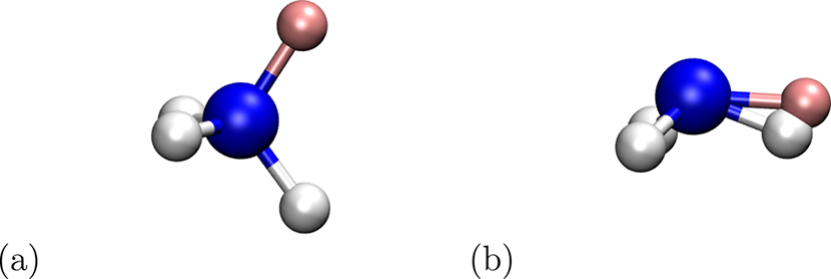
Alchemical transformation of methane (CH_4_) to ammonia (NH_3_) (see also Figure 2). The ammonia end state is shown (nitrogen, blue; hydrogen, gray; dummy atom, pink). (a) The “proper” geometry, where the dummy atom is positioned as it would be in methane. (b) One alternative position the dummy atom can adopt if attached only through nonredundant bonded terms, i.e., one bond and two angles.

Second, three degrees of freedom may simply be insufficient to maintain the dummy atom (group) in a position and orientation which is also meaningful at the corresponding native state. Consider again the transformation of methane to ammonia. With respect to the separability of the partition function (cf. Section 2.1.2), one of the three θ_H_i_–N–D_H__ angle terms has to be turned off. However, without this third, “redundant” θ_H_i_–N–D_H__ angle term, the dummy atom attached to ammonia switches between a “proper” (methane-like) geometry (Figure 7a) and positions more or less on top of one of the ammonia hydrogens (Figure 7b). The underlying reason for this flapping (which one can easily observe even during relatively short gas phase simulations) is that both geometries have the same average H–N–D angle values with respect to the two (nonredundant) angle bending energy terms that were kept.

A related situation is found when a dummy atom is attached by one bond and two bond angles to a central atom in a branched configurations, such as the transformation of ammonia to water depicted in Figure 5. The three internal coordinates lead to two possible Cartesian coordinates for the dummy atoms, one “above” and one “below” the plane, in which the water atoms are situated. If one attempts to avoid this by positioning the dummy atom in the same plane as the water atoms, coupling between the dummy atom and physical atom degrees of freedom arises (cf. the end of Section 2.1.2). This may be acceptable if the physical molecule is a rigid ring system but clearly should be avoided if the physical system is flexible. The theoretical analysis of the constraint arising in such cases (Sections 2.1.2 and 2.1 of the SI) suggested to use equilibrium values of 90° to position the dummy atom. In Section 2.2 of the SI, we also carry out a detailed analysis of the possible paths, along which flapping can occur for a dummy atom or group held by one bond stretching and two angle bending terms. The main result is that choosing 90° as the equilibrium value for the bond angle terms involving the dummy atom also results in the highest possible energy barrier and effectively prevents flapping.

#### Anchoring to a Free Rotator

**Figure 8:**
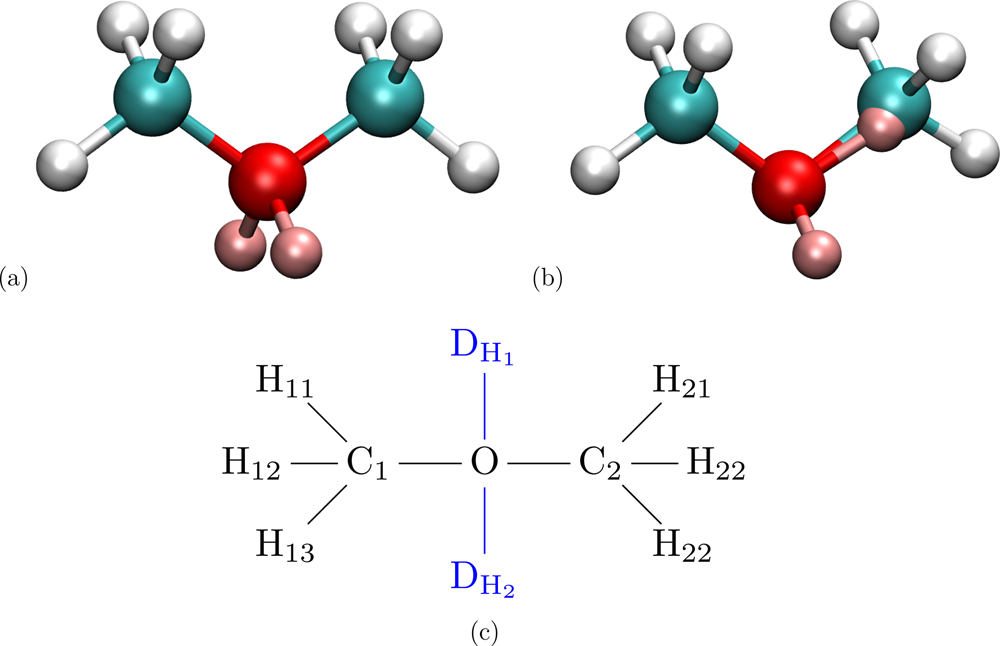
Dimethyl ether end state of the alchemical transformation of propane to dimethyl ether. (a) The dummy atoms (colored in pink) are positioned in the “proper” geometry, as they would be in propane. (b) Alternative geometry of the dummy atoms. (c) Nomenclature/atom labels used in the main text.

When a dummy atom or group is connected to the physical system by a bond stretching, angle bending, and dihedral angle term, there is a third, potential source for flapping. Consider the alchemical transformation of propane to dimethyl ether (Figure 8). We discuss the case of dummy atom D_H_1__; the situation for D_H_2__ is analogous. We anchor D_H_1__ to the physical molecule via the bond D_H_1__–O, the angle D_H_1__–O–C_1_, and the dihedral D_H_1__–O–C_1_–H_11_. The position of the dummy atom is thus linked to the methyl group at C_1_, and consequently, it is forced to partake in the rapid rotation of the methyl group. Because nonbonded interactions of the dummy atom are turned off at the dimethyl ether end state, the dummy atom is forced to follow rotamer interconversions of the physical methyl group, leading to positions as shown in Figure 8(b). This positioning next to C_2_ could only be prevented by a D_H_1__–O–C_2_ angle, which, however, would introduce a redundant degree of freedom coupling the dummy atom to the physical part of the system.

### 2.3 Toward a Systematic Approach: Types of Junctions

In the two preceding sections, we presented in detail the two guiding principles concerning the correct treatment of dummy atoms: separability of their contributions to the partition function and maintaining them in a well-defined position and orientation. On the basis of these on occasion contradictory requirements, we now outline best practices how to handle dummy atoms present in the most important types of alchemical transformations.

**Figure 9:**
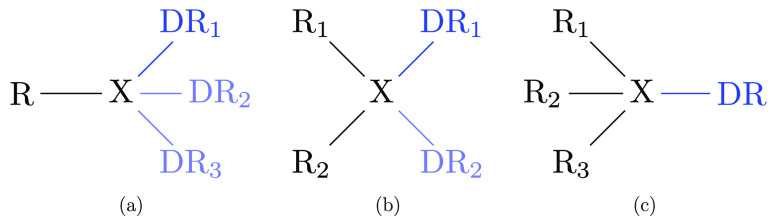
Schematic depiction of the junction types. (a) Terminal junction(s): solid blue represents a single terminal junction. More than one branch can be connected to the physical bridge atom X (DR_2_/DR_3_ shown in light blue). (b) Dual junction with one (solid blue) or two branches (solid and light blue) connected to X. (c) Triple junction.

We distinguish between the three cases depicted in Figure 9. Chemical moieties in the physical system are denoted as R_*i*_. *Dummy groups*, consisting of one or more dummy atoms, are labeled by analogy as DR_*i*_. The physical atom X directly connected to one or more dummy groups by one or more bond stretching terms is referred to as the *physical bridge atom*. The three cases shown in Figure 9 differ by the number of physical groups R_*i*_ bound to the bridge atom X. If there is only one such group, we refer to this as a *terminal* junction (Figure 9(a)); the cases of two and three physical groups bound to X are denoted as *dual* (Figure 9(b)) and *triple* junctions (Figure 9(c)), respectively. We note in passing that there might be other types of junctions with more than three physical moieties attached to the physical bridge atom, for example, when X is a hexavalent sulfur before the alchemical transformation. Related situations may arise when the mutation is set up in the dual topology paradigm, to which we return to in Section 2.4. While the physical groups connected to X can be part of a cyclic structure (e.g., R_1_ and R_2_ in Figure 9(b)), we assume that the dummy groups are always disjoint.

Clearly, the focus has to be the junction between the physical parts and the dummy groups, i.e., any bonded terms encompassing atoms of a physical group R_*i*_, the bridge atom X, and a dummy group DR_*i*_. Within a “dummy group” (a chemical moiety transformed completely into dummy atoms), there are only bonded interactions, for example, bond stretching, angle bending, and dihedral energy terms. Any energy terms involving only dummy atoms from the *same* dummy group can and should always be kept. By contrast, bonded terms between two dummy groups require care, as they will involve the physical bridge atom X. The dummy atom of a dummy group having a bond stretching term to X is referred to as *dummy bridge atom* and the bonded term itself as the *bridge bond*. Depending on the details of the alchemical transformation, i.e., the chemical nature of the initial and final states, there can be more than one dummy group connected the physical bridge atom in terminal and dual junctions. This is indicated by the use of solid and light blue colors for dummy groups in Figure 9. This distinction turns out to be only important for dual junctions; further details are, therefore, deferred to Section 2.3.2.

Before discussing terminal, dual, and triple junctions, in detail, we point out two additional considerations. First, the geometry of the dummy group(s), in particular, how dummy atoms are positioned relative to the physical atoms, does not necessarily have to be identical or similar to the geometry of the original molecule before the transformation. The relevant criterion is that the geometry of the dummy group itself, as well as its position and orientation with respect to the physical system remains defined; i.e., flapping must not occur. Second, on occasion, it will not be possible to prevent flapping *and* achieve factorization of the partition function (cf. the case of the methane to ammonia transformation). In such cases, we strive to keep potential errors minimal by suitable tweaking of the force field parameters.

#### 2.3.1 Terminal Junction

Many alchemical transformations lead to a terminal junction. In principle, one has to distinguish between a single, combined two or three terminal junctions (cf. Figure 9a). Prototypical examples for these three cases are methanol to methane (CH_3_–OH → CH_3_–**H**D), methylamine to methane (CH_3_–NH_2_ →CH_3_–**H**D_2_), and ethane to methane (CH_3_–CH_3_ → CH_3_–**H**D_3_), respectively, where the physical bridge hydrogen atom is marked in bold. In the case of terminal junctions, however, it turns out that there is no need to distinguish between these three cases. Here, we outline the general considerations and provide specific details for a nontrivial mutation, the alchemical transformation of hexane to propane, in Section 3.3.

A potentially problematic six-angle constraint can arise in the case of combined three terminal junctions (cf. Figure 9a), i.e., when there are three dummy groups attached to the physical bridge atom *X*. However, one can easily convince oneself that the constraint equation resulting in this case only couples angle degrees of freedom involving dummy atoms. The same is true for any six-angle constraint centered about a dummy bridge atom. Therefore, for a *terminal junction*, one can always keep *all* bond angle terms. If the force field employs Urey–Bradley terms, then any such term involving the bond *r*_R-X_ and one of the dummy bridge atoms (cf. Figure 9a) needs to be deleted (or, alternatively, its force constant be set to zero).

If all dihedral angles between physical atoms and the dummy atoms/groups were kept, various single-anchored and dual-anchored dihedral constraints about the bond *r*_R–X_ arise. In Section 2.1, when discussing the mutation of methylamine to methane, we showed that such coupling can be removed by deleting selected dihedrals. Specifically, one has to identify the *physical* atoms *two* bonds away from the physical bridge atom. For one of them, all dihedral angles reaching into the dummy group(s) can be kept. Any dihedral angle terms that originate from the respective other physical atoms and reach into the dummy group(s) have to be deleted. As already mentioned, with the exception of Urey–Bradley terms (if present), all other bonded terms, including *all* bond angles, can be kept.

#### 2.3.2 Dual Junction

##### Single Branch

The toluene to pyridine transformation discussed in Section 2.2 (Figure 6) serves as our example of a dual junction where a *single* dummy atom group (the methyl group of the toluene end state) is attached to the physical bridge atom (the pyridine nitrogen). One obvious difference to a terminal junction is that the physical bridge atom N is the vertex of a physical bond angle (C_1_–N–C_5_). Thus, we need to take care not to affect this angle with our dummy atoms.

There are two possibilities to anchor the dummy bridge atom D_C_ with respect to the physical molecule. The first option is to use a consecutive bond, angle, and dihedral angle term, for example, D_C_–N, D_C_–N–C_1_, or D_C_–N–C_1_–C_2_. To prevent flapping, the periodicity of the dihedral angle term has to be removed. As outlined in Section 2.2, one can replace it by a harmonic term or set the periodicity to one. In the calculations reported below, we chose the last option, in combination with increasing the force constant. Obviously, one needs to pay attention that this modified energy term positions the dummy atom in the correct geometry. Proceeding in this manner, the dummy atom contribution to the partition function remains a multiplicative factor, and flapping is prevented.

Alternatively, one can anchor the dummy bridge atom D_C_ by one bond and *two* bond angles, i.e., not using a dihedral angle at all. In our example (Figure 6), the bonded terms to position D_C_ are the bond D_C_–N and the angles D_C_–N–C_1_ and D_C_–N–C_5_. To avoid complications from additional nonredundant terms, all dihedral angle terms starting from D_C_ into the physical system need to be deleted or their force constants set to zero. However, this approach introduces the constraint eq 8. Since based on the geometry of toluene the dummy atom is in the same plane as the physical atoms, the two angles positioning the dummy atom will have an influence on the third, physical angle. As in this particular case where a ring system is involved, the practical effect may be small, but for nonaromatic sp^2^ carbons, such a constraint should be avoided. On the basis of the considerations of Sections 2.1.2 and 2.2 (see also the SI), the best way to mitigate the constraint, as well as to avoid flapping, is to set the equilibrium values of the bond angle terms to the dummy atom to 90°, together with increasing the corresponding force constants.

As for the remaining dummy atoms D_H_i__, the following considerations apply in either of the above two approaches. There is no six-angle constraint present involving physical as well as dummy angles. Concerning the dihedrals starting from D_H_i__ into the physical system, single-anchored as well as dual-anchored dihedral constraints need to be avoided. Similarly to what was discussed in the case of the terminal junction, all dihedral angle terms must either terminate at atom C_1_ or C_5_; the respective other dihedrals need to be deleted.

##### Two Branches

**Figure 10:**
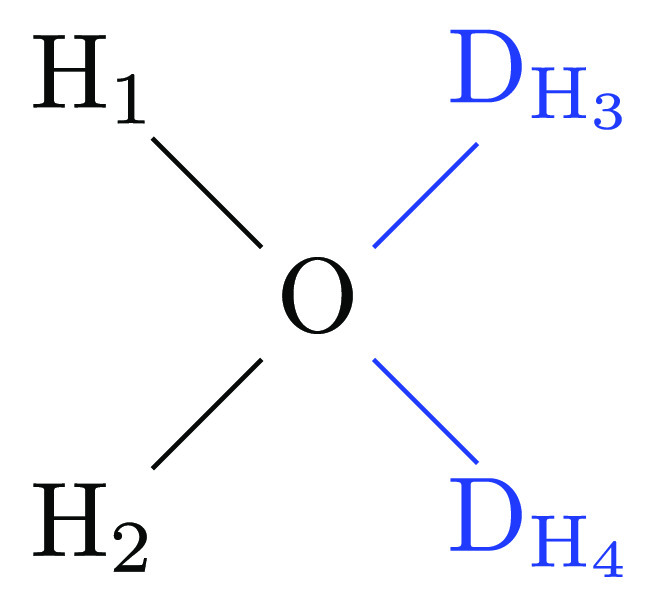
Methane to water transformation; the water endpoint with dummy atoms attached is shown.

We now turn to dual junctions where *two* dummy groups are attached to the physical bridge atom. The general rule in this case is that any bonded terms which depend on dummy atoms from both groups should be deleted. Then, each dual junction can be fully anchored to the physical molecule separately and thus treated independently by one of the two possibilities outlined above. Obviously, the two dummy groups should be placed in such a way that they do not collide. As the simplest possible example, we consider the alchemical transformation of methane to water (see Figure 10 which depicts the water endpoint). The methane carbon has been transformed into an oxygen, and the two superfluous hydrogens have been turned into dummy atoms D_H_3__ and D_H_4__. To decouple the two dummy “groups” (each consisting of a single atom), the angle D_H_3__–O–D_H_4__ needs to be deleted. In this manner, a six-angle constraint otherwise present is removed. Given the limited number of atoms, the only way to position the dummy atoms relative to the physical water is to use the bond O–D_H_3__ and the two angles H_1_–O–D_H_3__ and H_2_–O–D_H_3__, with analogous terms for D_H_4__. To maintain factorization of the partition function and to avoid flapping, the equilibrium values of the angles to dummy atoms need to be set to 90°, and we recommend increasing the force constant to 100 kcal mol^–1^ rad^–2^.

The methane to water transformation is the simplest example of a dual junction where two dummy groups are connected to the physical bridge atom. We already stressed that there should be *no* bonded interactions involving dummy atoms from both groups; in this simple example, the only term that needs to be removed is the angle D_H_3__–O–D_H_4__. If the two dummy groups were bigger, then this rule would extend to any dihedral angles involving dummy atoms from both groups; dummy atoms deeper in the dummy group should be anchored either to atoms of the physical molecule or to dummy atoms exclusively of their own branch.

If the physical region is larger, each of the dummy bridge atoms of the two dummy groups can also be attached to the physical bridge atom by a bond, an angle, and a dihedral energy term. Here, two of the angles anchoring the two dummy bridge atoms (one angle in each dummy group) are replaced by dihedrals. Thus, there is no six-angle constraint about the physical bridge atom present, which, for example, for methane to water, necessitated the removal of the angle D_H_3__–O–D_H_4__. In such cases, the general rule of decoupling dummy branches in a dual junction can be relaxed; when positioning each dummy atom using a bond, an angle and a dihedral energy term, one can safely include the angle bending term involving the physical and the two dummy bridge atoms. Any dihedral angle terms involving dummy atoms of both groups still need to be deleted.

As outlined earlier for toluene to pyridine, ambiguities resulting from the periodicity of the dihedral angle term need to be avoided. Further, as discussed for propane to dimethyl ether in Section 2.2, problems may arise if the “anchor” of the dihedral angle term is itself a rotamer, such as a methyl group. So while choosing a bond, an angle, and a dihedral angle to position a dummy atom is legitimate, one has to pay attention to the properties of the physical atoms which are available as anchor. The case of propane to dimethyl ether will be analyzed in detail in the Results.

#### 2.3.3 Triple Junction

For triple junctions, one has to distinguish two cases. If the physical system (the part of the physical system), which the dummy group is connected to, is planar (e.g., the physical bridge atom is a sp^2^ hybridized carbon), a clean separability of the partition function can be achieved while simultaneously avoiding flapping. If, on the other hand, the dummy atom is attached to a nonplanar moiety (e.g., the physical bridge atom is an amine nitrogen), then this clean separability is not possible anymore since at least one redundant angle term is needed to avoid flapping.

##### Planar Triple Junction

**Figure 11:**
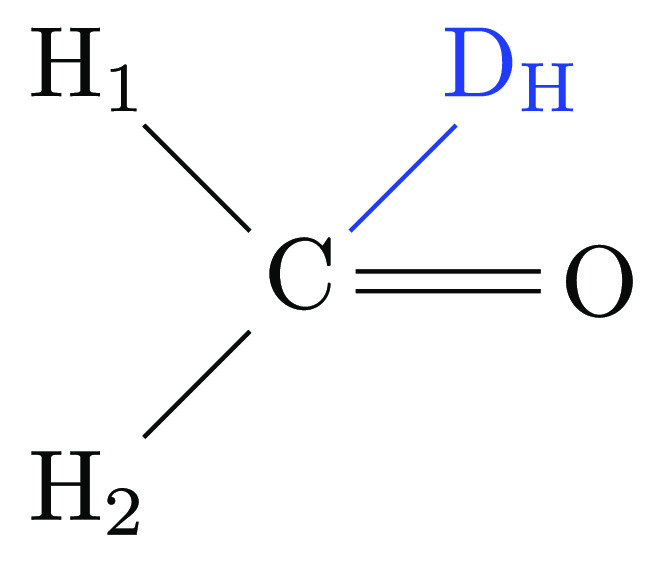
Methane to formaldehyde transformation; the formaldehyde endpoint is shown.

A simple example for the planar case is the alchemical transformation of methane to formaldehyde. The formaldehyde endpoint is depicted in Figure 11. One methane hydrogen has become the carbonyl oxygen; the fourth hydrogen has been transformed into the dummy atom D_H_. Since the physical system is planar in its minimum energy conformation, one can adopt the following strategy. One of the three physical atoms connected to the physical bridge atom is chosen, and *all* bonded terms between this atom and the dummy atom (or dummy atoms in case of a larger dummy group) are deleted. Here, we pick the oxygen O since it results in a symmetry of the remaining bonded terms, but this choice is arbitrary. Specifically, we delete the angle O–C–D_H_. Since the system is too small for dihedral angle terms, we anchor the dummy atom by means of bond C–D_H_ and angles H_1_–C–D_H_ and H_2_–C–D_H_. In a complete analogy with the dual junction, we set the equilibrium value of the two angle terms to 90° and use a reasonably hard force constant, for example, 100 kcal/(mol rad^2^). In this manner (cf. Section 2.1.2), we achieve exact factorization of the partition function. While we do not use the methane to formaldehyde transformation as one of our model systems, the approach just outlined is employed for the transformation of acetone to its tautomeric form 2-propenol, where the alchemical mutation involves changing a methyl (sp^3^ carbon) to a methylene group (sp^2^ carbon).

##### Nonplanar Triple Junction

An illustrative example of the nonplanar case is the alchemical transformation of methane to ammonia (see also Sections 2.1.2 and 2.2). There, we showed that flapping results if the six-angle constraint is removed by deleting one of the three angles involving a dummy atom. The “90°” trick we used for the dual junction as well as the planar case does not help here. If one anchors the dummy atom by means of two 90° angles with respect to two of the physical hydrogens, then distorted geometries as the one shown in Figure 7(b) still occur because of nitrogen inversion. Keeping all three angles H_i_–N–D, on the other hand, to prevent flapping hinders separability of the partition function. Here, the best one can do is to mitigate the effect of these angles on the physical system by tweaking their force field parameters. First, force constants of the H_i_–N–D angle terms should be set to a low value (<5 kcal/(mol rad^2^)). As there are three angles to anchor the dummy atom, it will be kept in a well-defined orientation relative to all three physical atoms (or groups for larger molecules) even by small force constants. Second, their equilibrium values should be adapted so that the minimum energy of the physical molecule is not affected. In practice, these adapted equilibrium values can be found by minimizing ammonia with the dummy atom attached, using very low H_i_–N–D dummy angle force constants, for example, 0.05 kcal/(mol rad^2^).

In both examples, there are no dihedral angles. For larger molecules, all dihedrals starting from the dummy bridge atom into the physical molecule need to be removed. If there are dihedral angles involving physical atoms and originating from dummy atoms connected to the dummy bridge atom, one has to be careful to avoid dual anchored dihedral constraints. Here, the situation becomes analogous to what was discussed for the dual junction, and the appropriate number of dihedrals needs to be deleted.

### 2.4 “Higher” Junctions and Dual Topology

Our discussion of terminal, dual, and triple junctions covers the majority of alchemical transformations when employing the single topology approach. Next, we investigate whether our findings and conclusions apply to the dual topology paradigm.^8,12^ The term “dual topology” with respect to setting up alchemical mutations is somewhat ambiguous. In one group of approaches, both end states, i.e., both solutes or ligands, are present simultaneously and held by restraints on top of each other. The interactions with the two entities, which never see each other, are turned off and on, respectively, as a function of the coupling parameter. An early example is work by Axelsen and Li;^27^ Riniker et al. used this approach in their enveloping distribution sampling method.^28^ The *Separated Topologies* method by Rocklin et al. falls into this category as well.^29^ This type of dual topology is also often employed in FES using QM/MM Hamiltonians (see, for example, ref (30)). An analysis whether the restraints applied to keep the two molecules on top of each other influence the properties of the respective end states is beyond the scope of this work.

**Figure 12:**
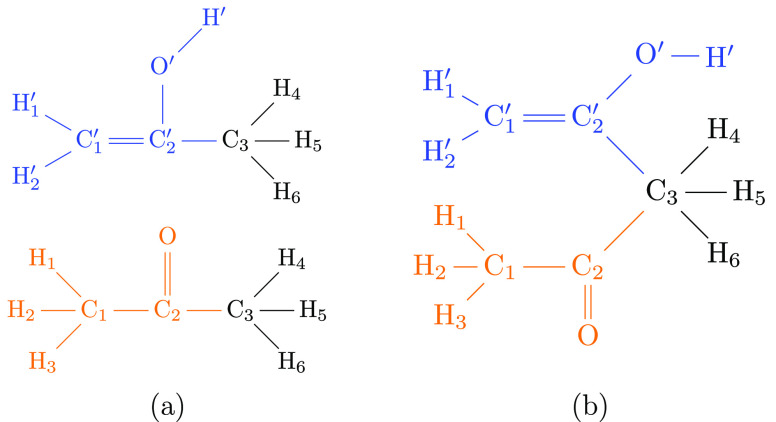
Dual topology setup for the alchemical transformation of acetone to 2-propenol. (a) The 2-propenol (top) and acetone (bottom) end states. (b) The dual topology hybrid structure used in this work.

However, since frequently the two end states are quite similar, for example, differing in one or two functional groups, one often defines a “common core”, for which either only a single set of coordinates is present, or where the corresponding atom positions are held exactly on top of each other by constraints.^31^ Only those atoms/groups which are different at the end states are present simultaneously and free to move independently; this is how the term dual topology was used and discussed by Shirts and Mobley^12^ or Boresch and Karplus.^9,10^ In this case, one ends up with “chimeric” hybrids to achieve the desired alchemical transformation; in Figure 12, we depict such a hybrid molecule for the mutation of acetone to 2-propenol.

The system consists of three parts: a common methyl group drawn in black and two “branches” corresponding to acetone and 2-propenol in orange and blue, respectively. At the acetone endpoint of the transformation, the orange branch is physical, and the blue part is equivalent to a dummy group. At the 2-propenol endpoint, the situation is reversed. If one extends our classification of junctions, we, therefore, have at each of the end states a “quadruple junction” (four physical moieties connected to the physical bridge atom C_3_).

Applying our framework to this case, the first thing to note is that in a dual topology approach the two branches, i.e., the acetone part (orange) and the 2-propenol part (blue) of the hybrid, must never have any interactions, neither nonbonded, nor bonded, with each other. Assuming this to be the case, we consider, for example, the acetone end state. Here, the 2-propenol branch is a dummy group, which we need to anchor with respect to the common methyl group (drawn in black in Figure 12). Since, in effect, we have eliminated the acetone branch from consideration, this reduces our task to that of a triple junction, with C_3_ the physical bridge atom. Since the geometry is nonplanar, strict factorization of the dummy group contribution to the partition function is not possible without incurring flapping (cf. Section 2.3.3). Thus, as in the methane to ammonia example, the force field parameters of the angle terms H_i_–C_3_–C′_2_ need to be modified so that the physical methyl group is perturbed as little as possible. To avoid additional dual anchored dihedral constraints, we choose one of the hydrogens from the shared methyl group, for example, H_4_. Dihedral angles extending from this atom into the 2-propenol dummy group are kept, whereas all dihedral angle terms to H_5_ and H_6_ are deleted. At the 2-propenol end state, the situation is reversed and analogous considerations apply to the acetone dummy group.

The single topology paradigm alchemical transformations, for example, involving pentavalent phosphor or hexavalent sulfur, could also lead to “higher-order” junctions. In such cases a similar strategy as just described for the dual topology case can be utilized. Specifically, bonded interactions of the dummy group with some of the physical groups branching from the physical bridge atom have to be removed, effectively reducing the connectivity of the dummy group to that of a triple junction.

## 3 Methods

### 3.1 Overview

To test and validate the proposed treatment of dummy atoms, we calculated relative solvation free energy differences for 12 pairs of solutes (see Table 1 for the model compounds, where we also introduce the abbreviations used in the following). In total, 24 alchemical transformations were carried out since in some cases we employed more than one way of handling the dummy atoms. The full list is compiled in Table 2, which contains also the number of water molecules present in the respective simulation system and the number of dummy atoms arising in the alchemical transformation, as well as the number of bonded energy terms involving dummy atoms that were modified (see Section 3.3 for further details).

**Table 1:** Abbreviations for Solutes

methane	MET	ethane	ETH	propane	PRP
hexane	HEX	toluene	TOL	water	WAT
methanol	MEOH	propanol	POL	phenol	PHE
dimethyl ether	DIM	pyridine	PYR	cyclohexadienone	CYC
ammonia	AMM	methylamine	MTA	acetone	ACE
2-propenol	PEOL				

**Table 2:** Overview of Simulations Carried out

	Alchemical transformation	Abbrev.	n_wat_^a^	n_dum_^b^	n_mod_^c^
Terminal junction	hexane → propane	HEX2PRP-1	565	9	0
	hexane → propane^d^	HEX2PRP-2	565	9	0
	toluene → methane	TOL2MET	567	10	0
					
Dual junction	ethane → methanol^e^	ETH2MEOH	567	2	4
	methane → water^e^	MET2WAT	567	2	4
	methane → water^e^,^f^	MET2WAT-qfs	567	2	4
	toluene → pyridine^e^	TOL2PYR-1	562	4	2
	toluene → pyridine^g^	TOL2PYR-2	562	4	1
	hexane → propenol^e^	HEX2POL-1	565	8	4
	hexane → propanol^d^	HEX2POL-2	565	8	0
	propane → dimethyl ether^e^	PRP2DIM-1	565	2	4
	propane → dimethyl ether^g^	PRP2DIM-2	565	2	0
	propane → dimethyl ether^g^,^h^	PRP2DIM-3	565	2	0
					
Triple junction	phenol → cyclohexadienone^e^	PHE2CYC	562	1	2
	ethane → methylamine^i^,^j^	ETH2MTA-1	567	1	3
	ethane → methylamine^d^	ETH2MTA-2	567	1	3
	methane → ammonia^i^,^j^	MET2AMM-1	567	1	3
	methane → ammonia^i^	MET2AMM-2	567	1	3
	methane → ammonia^d^	MET2AMM-3	567	1	0
	ethane → ammonia^i^,^j^	ETH2AMM-1	567	4	3
	ethane → ammonia^i^	ETH2AMM-2	567	4	3
	ethane → ammonia^d^	ETH2AMM-3	567	4	0
	acetone →2-propenol^e^	ACE2PEOL-1	565	1	2
					
Dual topology	acetone →2-propenol^i^,^j^	ACE2PEOL-2	565	6	6
	acetone →2-propenol^d^	ACE2PEOL-3	565	6	0

a Number of water molecules present in aqueous solution.

b Number of dummy atoms needed in the alchemical transformation.

c Number of bonded energy terms involving dummy atoms for which force field parameters were adjusted.

d Naive approach (see main text).

e Bond and two angles approach at the dual/triple junction.

f As MET2WAT, but with a time step of 0.25 fs.

g Bond, angle, dihedral approach at the dual/triple junction.

h Additional λ-states at the dimethyl ether end state (see main text).

i Equilibrium values of valence angle terms involving dummy atoms at the triple junction adjusted.

j Force constants of valence angle terms involving dummy atoms at the triple junction reduced to 3.55 kcal/rad^2^.

Proving the correctness of our proposed dummy anchoring scheme requires the comparison to reference values, in which errors resulting from dummy atoms cannot arise. As outlined in the Introduction, we resort to the thermodynamic cycles shown in Figure 1. Specifically, for a pair of solutes L1 and L2, we compute the relative free energy difference of solvation *ΔΔA*_*solv*_^*L1*→*L2*^ both as the difference of the two absolute solvation free energies, *ΔΔA*_*solv*_^*L1*→*L2*^ = *ΔA*_4_–*ΔA*_3_, as well as along the alchemical paths, i.e., *ΔΔA*_*solv*_^*L1*→*L2*^ = *ΔA*_2_^′^ – *ΔA*_1_^′^. In calculations of absolute solvation free energy differences (*ΔA*_3_, *ΔA*_4_), ambiguities as to how to handle dummy atoms do not arise. Dummy atoms are only present for the corresponding alchemical transformations *ΔA*_1_^′^ and *ΔA*_2_^′^. Our check for correctness is whether the equality *ΔA*_2_^′^–*ΔA*_1_^′^ = *ΔA*_4_–*ΔA*_3_ is fulfilled. Systematic, i.e., statistically significant, deviations of this identity will indicate nonseparability of the partition function and/or the presence of flapping. To test for significance of the two different treatments of dummy atoms, we performed Welch’s *t*-test^32^ on the two set of results.

For all 12 systems, we carry out calculations following the best practice outlined in Theory. As mentioned in the Introduction, one rarely finds details how dummy atoms are treated in practice. One strategy we have seen and used on occasion ourselves is to keep all bonded terms to dummy atoms, i.e., not to remove any redundant bonded terms and/or adapt equilibrium values or force constants. For several systems, we report results using this approach, which we refer to as “naive”. Full details are presented in Section 3.3.

Before continuing, we outline two quick checks concerning various aspects discussed in Theory. We strongly recommend them as they take little time and can prevent many problems in production simulations. Both need to be carried out for just the solute (or ligand) in the gas phase, so the computational cost is negligible. (1) With respect to the correct factorization of the partition function, the following zeroth order criterion needs to be fulfilled; the minimum energy conformation of the solute (or ligand) must be the same, regardless whether dummy atoms are present or not. This can be tested as follows. Minimize the physical solute (no dummy atoms present); this is the reference geometry and minimum energy. Then, repeat the minimization for the solute with dummy atoms attached. The presence of the dummy atoms may contribute to the energy of the system, but they must not affect the resulting geometry of the physical molecule. Thus, upon removal of the dummy atoms (e.g., in CHARMM using the DELEte ATOM command), the energy and geometry of the remaining, physical system—*without* further minimization!—must be exactly the same as that obtained during the reference calculation. We stress that this is a *necessary*, but not a sufficient, criterion. Even if the dummy atoms do not hinder the physical system to adopt the minimum energy geometry, they can still affect other conformations and/or the flexibility of the physical system; one such case is encountered in the alchemical transformation of methane to ammonia. (2) We also recommend carrying out an exploratory MD simulation of the solute (ligand) with dummy atoms in the gas phase. If flapping occurs for whatever reason, this is detected easily within a few seconds or at most minutes of computer time.

### 3.2 Common Simulation Setup

All simulations were carried out with CHARMM.^33^ Specifically, the PERT module and the associated PSSP soft-core model^34,35^ were used for all free energy calculations reported here. For most solutes listed in Table 2, there are topologies and force field parameters in the CHARMM CGenFF force field.^24−26^ The exceptions are methane, for which the topology and parameters were trivially constructed by analogy, as well as 2-propenol (tautomeric form of acetone in the ACE2PEOL transformation) and cyclohexadienone (tautomer of phenol in PHY2CYC). Topologies and parameters for these compounds were generated with the CGenFF interface at paramchem.org.^24−26^

Calculations in solution utilized a cubic water box containing 572 TIP3 waters.^36,37^ Its size (side length of ≈26 Å) is sufficiently large so that the default CHARMM cutoff scheme can be used (cf. below). Waters overlapping with the solutes were deleted. For each alchemical transformation studied, the number of water molecules was the same during the calculation of the relative free energy difference, as well as the corresponding calculations of absolute solvation free energies (for the detailed list, see Table 2). Electrostatic interactions were calculated by Ewald summation^38^ with κ = 0.34 Å^–1^, employing the particle mesh Ewald method (PME)^39^ on a 32 × 32 × 32 grid. Lennard-Jones interactions were switched off between 10 and 12 Å.^40^ The temperature was kept around 300 K using a Nosé–Hoover thermostat^41^ with a thermal piston mass of 1000 kcal ps^2^ mol^–1^. Pressure was controlled by a Langevin piston barostat^42^ to be around 1 atm. The mass of the pressure piston was 400 amu, and the collision frequency was 20 ps^–1^. In all simulations, a time step of 1 fs was used, with the exception of MET2WAT-qfs, where the time step was reduced to 0.25 fs. The solute molecules were fully flexible; waters were kept rigid using SHAKE.^43^

In the gas phase, no cutoffs were applied to the nonbonded interactions. Temperature was controlled using Langevin dynamics,^44^ with the friction coefficient set to 5 ps^–1^ on all atoms. The temperature of the heatbath as well as the integration time step were chosen consistently with the simulations in solution.

All free energy calculations (gas phase, solvated phase, calculations of relative, and absolute free energy differences) employed the following protocol. Each alchemical transformation was carried out using 21 equidistant λ-states, λ = 0.00, 0.05, ..., 1.00, and was repeated five times, starting the simulations from different initial random velocities. Each state was equilibrated for 200 ps, followed by a 2 ns production run. The derivatives needed for TI were accumulated on the fly by the CHARMM PERT module,^33^ and coordinates were saved every 50 steps. From these the energy differences needed for BAR were obtained in a postprocessing step. Soft cores were used throughout as for larger, flexible molecules they are necessary even in the gas phase. Since our target is internal consistency in closing the thermodynamic cycle Figure 1(c), we did not apply long-range corrections for the Lennard-Jones terms.

### 3.3 Simulated Systems

A full description of each alchemical transformation can be found in Figures 5–17 of the SI; here, we point out the most salient features, focusing on representative examples. These illustrate the general considerations given earlier for concrete cases and show how to read the detailed information in the SI. Table 3 lists all changes to bonded terms involving dummy atoms for the four transformations discussed below.

**Table 3:** Treatment of Bonded Terms Involving Dummy Atoms of Four Representative Examples

	HEX2PRP-1	TOL2PYR-1	TOL2PYR-2	ACEPEOL-1
Deleted	D_C_5__–H_32_–C_3_–H_31_	D_C_–N–C_1_–C_2_	D_C_–N–C_5_	
	D_C_5__–H_32_–C_3_–H_32_	D_C_–N–C_1_–H_1_	D_C_–N–C_1_–H_1_	
	D_H_41__–H_32_–C_3_–H_31_	D_C_–N–C_5_–H_5_	D_C_–N–C_5_–H_5_	D_H_–O–C_2_–C_1_^c^
	D_H_41__–H_32_–C_3_–H_32_	D_C_–N–C_5_–H_4_	D_C_–N–C_5_–C_4_	D_H_13__–C_1_–H_12_^d^
	D_H_42__–H_32_–C_3_–H_31_	D_H_1__–D_C_–N–C_5_	D_H_1__–D_C_–N–C_5_	D_H_13__–C_1_–C_2_–C_3_^d^
	D_H_43__–H_32_–C_3_–H_32_	D_H_2__–D_C_–N–C_5_	D_H_2__–D_C_–N–C_5_	D_H_13__–C_1_–C_2_–O^d^
		D_H_3__–D_C_–N–C_5_	D_H_3__–D_C_–N–C_5_	
				
Modified	None	D_C_–N–C_1_^a^	D_C_–N–C_1_–C_2_^b^	D_H_13__–C_1_–H_11_^d^^,^^a^
		D_C_–N–C_5_^a^		D_H_13__–C_1_–C_2_^d^^,^^a^

a Equilibrium angle set to 90°, force constant increased to 100 kcal/mol/rad^2^.

b Periodicity set to 1, force constant raised to 100 kcal/mol, and equilibrium angle set to 0°.

c Acetone endpoint.

d 2-propenol endpoint.

#### Terminal Junction: Hexane to Propane

**Figure 13:**
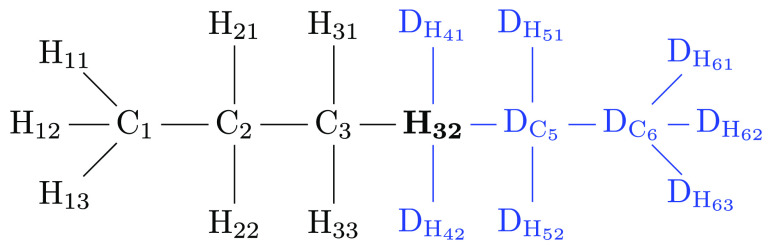
Propane endpoint of the alchemical transformation of hexane to propane; all dummy atoms are explicitly shown.

As a nontrivial example of a *terminal junction*, we consider the transformation of hexane to propane (HEX2PRP-1); the propane endpoint is shown in Figure 13. In Section 2.3.1, we showed that for terminal junctions all bond angle terms involving dummy atoms can be kept. However, selected dihedral angle terms need to be deleted. As described in Section 2.3.1, one has to identify the *physical* atoms *two* bonds away from the physical bridge atom; for the propane end state, these are C_2_, H_31_, and H_33_. We decided to keep the dihedrals φ_D_i_–H_32_–C_3_–C_2__ with D_i_ denoting dummy atoms D_C_5__, D_H_41__, and D_H_42__. This choice is motivated as it is the dihedral angle along the alkane main chain. Therefore, all dihedrals D_i_–H_32_–C_3_–H_31_ and D_i_–H_32_–C_3_–H_33_ have to be deleted (or the respective force constants need to be set to zero). This results in the six dihedral angles in the row “Deleted” in Table 3. For a terminal junction, there is no need to modify a force field term; hence, there is no entry in the row “Modified”. For the second terminal junction example, TOL2MET, we proceeded analogously (for details, see Figure 6 of the SI).

#### Dual Junction: Toluene to Pyridine

TOL2PYR serves as our representative example of a *dual junction*. As discussed in Section 2.3.2, two approaches to handle dummy atoms are possible in this case; details for both of them are listed in Table 3. The first, using one bond and two angles to position the dummy bridge atom D_C_, is referred to as TOL2PYR-1. The equilibrium values of the bond angles involving the dummy atom were changed to 90°; to make sure flapping is avoided, the force constant was increased to 100 kcal(mol rad^2^)^−1^. Since these two angles fully anchor D_C_, all dihedrals originating from pyridine and ending in D_C_ were deleted. Additionally, to avoid dihedral constraints originating from the hydrogens D_H_i__ of the dummy methyl group (Figure 6c), all dihedrals ending in C_5_ were removed.

Alternatively, the dummy bridge atom D_C_ can be positioned by one bond, one angle, and one dihedral angle. Details for this approach are listed in entry TOL2PYR-2 in Table 3. Compared to TOL2PYR-1, these were the differences: The D_C_–N-C_5_ angle was deleted, and the D_C_–N-C_1_-C_2_ dihedral kept instead. Thus, D_C_ was anchored by the bond D_C_–N, bond angle D_C_–N–C_1_, and dihedral D_C_–N–C_1_–C_2_. The angle term D_C_–N–C_1_ was not modified. The force constant of the dihedral angle was increased to 100 kcal/mol, its periodicity changed to 1, and its equilibrium value set to 0°, maintaining the dummy atom in the plane of the pyridine ring and preventing flapping to a position inside the ring (cf. Section 2.2).

Since dual junctions can arise frequently in practice, several calculations involving them were carried out (Table 2). For all of them, the bond and two angles approach was used. The calculations MET2WAT, HEX2POL, ETH2MEOH, and PRP2DIM all involve *two* dual junctions, i.e., two groups of dummy atoms connected to the physical bridge atom. As stressed in Section 2.3.2, any bonded terms involving dummy atoms in both of these two branches need to be removed. For example, for the transformation of methane to water (MET2WAT), the angle D_H_3__–O–D_H_4__ was deleted (cf. Section 2.3.2). Full details for MET2WAT, HEX2POL, ETH2MEOH, and PRP2DIM can be found in the SI. As is discussed further in Results, MET2WAT is the only case where we could not close the thermodynamic cycle within statistical error bars when applying best practice. Therefore, this calculation was repeated with a time step of 0.25 fs (as opposed to the regular 1 fs); this calculation is referred to as MET2WAT-qfs. For HEX2POL, we also report results using the “naive” approach, i.e., keeping all bonded terms involving dummy atoms using the force field parameters of the corresponding physical terms at the hexane end state. Any Urey–Bradley terms involving a dummy and two physical atoms were removed even in the naive approach because of the reasons discussed in Section 2.1.2. Finally, for PRP2DIM we also used the bond–angle–dihedral approach for each of the two dummy atoms. In this particular system, one has to anchor the dihedral in a freely rotable methyl group, a potential source of flapping (Section 2.2). Therefore, in addition to the standard protocol (PRP2DIM-2), a second set of calculations using a finer λ-spacing near the dimethyl-ether endpoint was carried out (PRP2DIM-3). Specifically, between λ = 0.9 and λ = 1.0, a 0.005 λ-spacing was used, resulting in a total of 39 λ-states. Since the dummy atoms were anchored independently and since we did not change the periodicity of the dihedral angles, the dummy atoms could adopt positions on top of each other. To prevent this, we also kept the angle D_H_1__–O–D_H_2__. As outlined in Section 2.3.2, this is the one exception of the general rule to delete all terms involving dummy atoms of different groups; the factorization of the partition function is not affected.

#### Triple Junction: Acetone to 2-Propenol

The final example detailed in Table 3, ACE2PEOL-1, has a planar *triple junction* at the enol end state. As described in Section 2.3.3, this situation can be handled exactly by employing a bond and two angles approach, which was done here. In analogy to what was described for TOL2PYR-1, the equilibrium values of the two bond angles to the dummy atom were changed to 90°, and their force constants were increased to 100 kcal/(mol rad^2^). The dummy atom present at the acetone endpoint is a terminal junction, which was treated according to the general rules for this case. Our second example involving tautomerism, PHE2CYC, was treated analogously (SI).

Nonplanar triple junctions arise, for example, when alchemically morphing an alkane to an amine; several such transformations (ETH2MTA, MET2AMM, and ETH2MAMM; Table 2) were studied. For each of them, free energy differences were computed (i) using a naive approach, i.e., keeping all bonded terms using the physical bond angle parameters for the dummy atoms, though deleting Urey–Bradley terms (MET2AMM-3, ETH2MTA-2, ETH2AMM-3), as well as (ii) our best practice approach (MET2AMM-1, ETH2MTA-1, ETH2AMM-1), adapting the equilibrium bond angles of the D–N–D angles and lowering their force constants to 3.55 kcal mol^–1^rad^–2^ to disturb the amine end state as little as possible. In MET2AMM-2 and ETH2AMM-2, we additionally explored the effect of adjusting the equilibrium angles but keeping the force constants at their original values.

While most of our examples employed the single topology paradigm, ACE2PEOL was also studied using a dual topology setup (cf. Section 2.4). The two branches, acetone (orange) and 2-propenol (blue in Figure 12) were not allowed to interact, neither by bonded nor nonbonded terms. Thus, this alchemical transformation features nonplanar triple junctions at the methyl common core (black) at both endpoints. The calculations labeled ACE2PEOL-2 were set up according to our best practice; i.e., equilibrium values and force constants of the angle terms involving dummy atoms (angles C_2_–C_3_–H_i_ of the acetone branch and angles C′_2_–C_3_–H_i_ (*i* = 1,···,3) of the 2-propenol branch) were adapted (cf. MET2AMM-1 above). All dihedrals ending in either H_5_ or H_6_ of the shared methyl group were deleted to remove the respective dihedral constraints. By contrast, in ACE2PEOL-3, all bonded terms were kept unchanged; i.e., this is the “naive” approach for a dual topology setup.

## 4 Results

Table 4 lists results for each of the relative solvation free energy differences studied using the or one of the best practice approaches. We report the results along the alchemical path (*ΔΔG*_*solv*_), as well as the difference of the respective absolute solvation free energy differences (*ΔΔG_solv_^abs^*), together with the difference *ΔΔG*_*solv*_ – *ΔΔG_solv_^abs^*, which ideally should be identically zero. The detailed raw results for each of the systems, free energy differences in gas phase and aqueous solution, obtained with TI and BAR, are reported in Section 5 of the SI (Figures 5–17). Any deviation of the cycle closure *ΔΔG_solv_* – *ΔΔG_solv_^abs^* needs to be gauged together with the statistical uncertainty, which can also be found in the table. A *p*-value ≥ 0.05 (rightmost column in Table 4) indicates that the cycle closure error is statistically indistinguishable from zero. The agreement in relative solvation free energy differences along the two pathways is excellent with the single exception of MET2WAT, where cycle closure within statistical error bars was obtained only when using a smaller time step (MET2WAT-qfs). Given the small size of the solute (3/5 atoms), we suspect that this may result from hidden difficulties maintaining its average temperature. Our model transformations involve up to 10 dummy atoms (TOL2MET); hence, overall, our results indicate that any contributions from dummy atoms cancel from the double free energy difference of interest if best practice is followed.

**Table 4:** Comparison of Solvation Free Energy Differences from Absolute and Relative Alchemical Transformations Using the or One Best Practice Approach Described in the Main Text^a^

Transformation	*ΔΔG*_*solv*_	*ΔΔG_solv_^abs^*	*ΔΔG*_*solv*_ – *ΔΔG_solv_^abs^*	*p*^b^
HEX2PRP-1	–0.47 ± 0.04	–0.49 ± 0.06	0.02 ± 0.07	0.55
TOL2MET	2.46 ± 0.02	2.50 ± 0.05	–0.04 ± 0.05	0.15
ETH2MEOH	–6.93 ± 0.03	–6.94 ± 0.02	0.01 ± 0.04	0.55
MET2WAT-qfs	–9.24 ± 0.03	–9.27 ± 0.02	0.03 ± 0.04	0.11
MET2WAT	–9.18 ± 0.04	–9.27 ± 0.02	0.09 ± 0.04	0.00
TOL2PYR-1	–4.69 ± 0.02	–4.67 ± 0.04	–0.02 ± 0.04	0.36
HEX2POL-1	–7.09 ± 0.04	–7.11 ± 0.07	0.02 ± 0.09	0.60
PRP2DIM-1	–3.33 ± 0.02	–3.36 ± 0.03	0.03 ± 0.04	0.11
PHE2CYC	–0.01 ± 0.02	0.01 ± 0.06	–0.02 ± 0.06	0.51
ETH2MTA-1	–5.90 ± 0.02	–5.87 ± 0.02	–0.03 ± 0.03	0.05
MET2AMM-1	–6.22 ± 0.02	–6.19 ± 0.02	–0.03 ± 0.03	0.05
ETH2AMM-1	–6.08 ± 0.02	–6.06 ± 0.02	–0.02 ± 0.03	0.15
ACE2PEOL-1	1.03 ± 0.02	1.05 ± 0.03	–0.02 ± 0.04	0.25

a See Table 2 for abbreviations used.

b *p*-value obtained using Welch’s *t* test (cf. main text).

Free energy differences for additional setups are reported in Table 5. Again, all raw results can be found in Figures 5–17 of the SI. First, for some dual junctions (TOL2PYR-2, PRP2DIM-2, and PRP2DIM-3), we repeated the calculations with the alternative best practice approach, i.e., bond, angle, dihedral setup instead of bond and two angle setup. In all cases, cycle closure is excellent; the special case of the two propane to dimethyl ether calculations is discussed separately below. Table 5 also contains results for several transformations using a naive setup, where all bonded terms were kept. In the case of the terminal (HEX2PRP-2) and the dual junction (HEX2POL-2), this has no apparent effect on cycle closure; i.e., in both cases, the relative solvation free energy difference remains unaffected. This is also the case when a dual topology setup was used. The results for ACE2PEOL-2 (best practice result) and ACE2PEOL-3 (naive approach) are for all practical purposes identical, and in both cases, the cycle closure criterion is fulfilled.

**Table 5:** Comparison of Solvation Free Energy Differences in kcal/mol from Absolute and Alchemical Transformations Using Alternative Approaches^a^

Transformation	*ΔΔG*_*solv*_	*ΔΔG_solv_^abs^*	*ΔΔG*_solv_ – *ΔΔG_solv_^abs^*	*p*^b^	*ΔΔG_solv_*^c^
HEX2PRP-2^d^	–0.50 ± 0.05	–0.49 ± 0.06	–0.01 ± 0.08	0.78	–0.47 ± 0.04
TOL2PYR-2	–4.67 ± 0.02	–4.67 ± 0.04	0.00 ± 0.04	1.00	–4.69 ± 0.02
HEX2POL-2^d^	–7.09 ± 0.05	–7.11 ± 0.07	0.02 ± 0.09	0.62	–7.09 ± 0.04
PRP2DIM-2	–3.36 ± 0.02	–3.36 ± 0.03	0.00 ± 0.04	1.00	–3.33 ± 0.02
PRP2DIM-3	–3.34 ± 0.02	–3.36 ± 0.03	0.02 ± 0.04	0.25	–3.33 ± 0.02
ETH2MTA-2^d^	–5.91 ± 0.02	–5.87 ± 0.02	–0.04 ± 0.03	0.01	–5.90 ± 0.02
MET2AMM-2	–6.05 ± 0.02	–6.19 ± 0.02	0.14 ± 0.03	0.00	–6.22 ± 0.02
MET2AMM-3^d^	–5.60 ± 0.02	–6.19 ± 0.02	0.59 ± 0.03	0.00	–6.22 ± 0.02
ETH2AMM-2	–5.91 ± 0.02	–6.06 ± 0.02	0.15 ± 0.03	0.00	–6.08 ± 0.02
ETH2AMM-3^d^	–5.69 ± 0.02	–6.06 ± 0.02	0.37 ± 0.03	0.00	–6.08 ± 0.02
ACE2PEOL-2	1.02 ± 0.02	1.05 ± 0.03	–0.03 ± 0.04	0.11	1.03 ± 0.02
ACE2PEOL-3^d^	1.05 ± 0.02	1.05 ± 0.03	0.00 ± 0.04	1.00	1.03 ± 0.02

a See Table 2 for abbreviations used.

b *p*-value obtained using Welch’ *t* test (cf. main text).

c Best practice results from Table 4.

d Naive approach.

By contrast, statistically significant deviations were obtained in the case of triple junctions (ETH2MTA-2, ETH2AMM-3, MET2AMM-3), where the relative solvation free energy obtained along the alchemical path deviated up to 0.6 kcal/mol when bonded terms involving the dummy atom(s) were treated naively. To understand these findings, let us focus on methane to ammonia. Here, one hydrogen becomes a dummy atom. To avoid flapping, all bond angle terms present in methane need to be kept. If this is done by adopting the force field parameters which correspond to the physical end state, i.e., what we refer to as the naive approach MET2AMM-3, one introduces strain on the physical ammonia molecule, since the θ_D–N–H_i__ angle terms influence the physical θ_H_i_–N–H_j__ angles of ammonia and distort them from their equilibrium value. In other words, naively choosing the methane bond angle parameters introduces a geometrical strain. One would expect that the resulting artifacts can be removed by adapting the equilibrium value of the θ_D–N–H_ angle term so that the minimum energy geometry of ammonia is not affected any longer. This setup was tested in MET2AMM-2 (as well as ETH2AMM-2). The equilibrium angle for the dummy atom terms was adapted, but the force constant was kept at the methane value. As one sees in Table 5, this leads to an improvement, but a statistically significant cycle closure error remains. If, on the other hand, one follows the best practice outlined in Section 2.3.3, i.e., equilibrium values for the θ_D–N–H_ angles are adapted *and* the force constant is reduced, then one obtains excellent cycle closure (see the MET2AMM-1 and ETH2AMM-1 results in Table 4), even though the partition function does not factor exactly into contributions from the physical system and terms involving dummy atoms.

The effects, which the different treatments of dummy atoms have on the ammonia and methylamine endpoints, are easily understood by looking at their average dipole moments in aqueous solution, which we report in Table 6. In the case of the best practice treatment (MET2AMM-1, ETH2AMM-1, ETH2MTA-1), the average dipole moments are almost identical for the pure solute and the solute with the dummy atom(s) attached. By contrast, restraining the flexibility of the ammonia/amine angles (MET2AMM-2/3, ETH2AMM-2/3, ETH2MTA-2) lowers the average dipole moment, which leads to a more positive relative solvation free energy difference.

**Table 6:** Comparison of Average Dipole Moments (in Debye) of Ammonia [AMM] and Methylamine [MTA] End States in Aqueous Solution Using Different Treatments of Dummy Atoms with the Corresponding Solutes without Dummy atoms

	with dummy atoms	solute without dummy atoms
ETH2MTA-1^a^	2.03 ± 0.00	2.04 ± 0.00
ETH2MTA-2^b^	1.97 ± 0.00	2.04 ± 0.00
MET2AMM-1^a^	2.32 ± 0.01	2.33 ± 0.00
MET2AMM-2^c^	2.23 ± 0.00	2.33 ± 0.00
MET2AMM-3^b^	2.11 ± 0.00	2.33 ± 0.00
ETH2AMM-1^a^	2.32 ± 0.00	2.33 ± 0.00
ETH2AMM-2^c^	2.23 ± 0.00	2.33 ± 0.00
ETH2AMM-3^b^	2.16 ± 0.00	2.33 ± 0.00

a Best practice.

b Naive approach, see main text.

c All dummy angles of the triple junction adjusted to fit the physical equilibrium angle without weakening the force constant.

Finally, we return to the PRP2DIM-2 and PRP2DIM-3 results reported in Table 5. For both, the respective *p*-value > 0.05 suggests that cycle closure was achieved. However, looking at the detailed results for PRP2DIM-2 (Figure 11 of the SI), there are systematic differences both in gas phase and aqueous solution of ≈0.8 kcal/mol between TI and BAR. For PRP2DIM-3, on the other hand, the BAR and TI results agree, and the PRP2DIM-3 values also agree with the BAR result for PRP2DIM-2, suggesting that the problem affects TI only. The difference between the two setups is not the treatment of dummy atoms, which is identical in both cases (each of them is held separately by the bond, angle, dihedral approach, plus the angle_DH_1_-O-DH_2__ preventing the dummy atoms from sitting on top of each other) but the number of λ-states used, 39 (PRP2DIM-3) vs 21 (PRP2DIM-2) (cf. Methods). A plot of ⟨∂*U*/ ∂λ⟩_λ_ for the two cases is shown in Figure 18 of the SI. One sees that the integrand is well behaved until λ = 0.95, then drops off steeply by almost 100 kcal/mol as it approaches λ = 1. While not shown, individual error bars are very low, which is reflected by the high precision of the TI results for both PRP2DIM-2 and PRP2DIM-3 reported in Figure 11 of the SI. If using only 21 λ-states (PRP2DIM-2, red line/dots in Figure 18 of SI), no numerical quadrature method can follow the strong variation of the integrand. When using more λ-states (PRP2DIM-3), the integrand is sampled sufficiently often (blue line/dots in Figure 18 of SI), and numerical quadrature gives the correct result. The behavior of the integrand toward λ → 1 is caused by the nature of the dihedral angle terms employed to keep the dummy atoms connected to the physical system (cf. Section 2.2). The anchor point (by necessity) is a methyl group, which at the dimethyl ether endpoint is an almost free rotator. Hence, the dummy atoms are coupled to this rapid movement, resulting in the highly negative values of the integrand. As opposed to other cases of flapping, this motion is so rapid that the values of the integrand are very precise. The error observed in the case for PRP2DIM when using TI arises because of the insufficient number of λ-states in the rapidly varying region of the integrand. In the case of PRP2DIM-3, a larger number of λ-values was used in the problematic range; therefore, both TI and BAR gave identical results. The error observed for PRP2DIM-2 is almost identical in gas phase and aqueous solution, resulting in fortuitous error cancellation and apparent cycle closure for PRP2DIM-2 as reported in Table 5.

## 5 Concluding Discussion

Dummy atoms are required in almost all alchemical transformations, i.e., when calculating relative free energy differences. Figure 1a contrasts the theoretical situation (no dummy atoms) with practice (Figure 1b, dummy atoms present). The validity of relative free energy calculations rests on the equivalence of the two cases; i.e., the presence of dummy atoms must not affect the result. We showed that two requirements have to be fulfilled for this to hold true. On the one hand, the contribution of dummy atoms to the partition function has to be multiplicative; in this case, any effect they may have on a single free energy difference will cancel from the final double free energy difference of interest. On the other hand, the position and orientation of dummy atoms need to remain well defined; what we refer to as “flapping” has to be avoided.

In the single topology paradigm to set up alchemical transformations, we suggest to distinguish between terminal, dual, and triple junctions. In the first two cases, one can always cleanly separate degrees of freedom from dummy atoms and physical atoms while avoiding flapping. This is also the case for “planar” triple junctions, i.e., when the physical atoms forming the triple junction at the endpoint are coplanar. In the nonplanar case, one redundant bonded energy term needs to be kept to avoid flapping, which prevents the desired factorization of the partition function. Even in this case systematic errors can be kept negligibly small by a suitable adjustment of the force field terms involving dummy atoms. In biomolecular simulations, bonds are frequently held rigid by holonomic constraints to increase the integration time step. Complications from nonredundant degrees of freedom result from angle bending or dihedral angle terms, which are not subject to constraints. Therefore, all considerations and conclusions apply regardless of whether bonds are flexible or held rigid by SHAKE,^43^ RATTLE,^45^ or similar means.

In the literature, dummy atoms and side effects resulting from them are mostly discussed in connection with single topology setups. As mentioned in Section 2.4 the term dual topology is somewhat ambiguous. Our considerations do not apply to approaches where complete molecules (ligands or solutes) are duplicated and loosely held on top of each other by restraints.^28,29^ However, dual topology also refers to duplicating just the functional groups which are different between the two end states (cf. Figure 12). For all practical purposes, the atoms in the noninteracting groups at the endpoints of such a dual topology setup are also dummy atoms. In fact, in these varieties of dual topology, there are usually more dummy atoms present than in the equivalent single topology setup, as has been pointed out before.^12,13^ The dual topology setup of Figure 12 could be considered a “quadruple junction”. Since, however, any bonded terms between the two groups representing the two end states are removed, the dummy atoms can and should be treated as two distinct triple junctions.

Beyond these general considerations, there are some special cases which depend on the details of the force field employed. If the description of valence angle terms is augmented by Urey–Bradley terms, unwanted constraints are easily introduced. As outlined in Section 2.1.2, when a valence angle consists of two physical atoms and one dummy atom, no Urey–Bradley energy term should be applied in addition to the regular bond angle term. Almost all force fields employ improper dihedral angle energy terms under selected circumstances. Dihedral and improper dihedral angles are in many regards quite similar although, for example, in the CHARMM force field, a harmonic potential is used for impropers. From a chemical point of view, the difference to a proper dihedral is that one of the “bonds” of an improper dihedral is not a covalent bond. It is certainly possible and legitimate to attach a dummy atom to the physical system using a bond stretching, a valence angle, and an improper dihedral angle term. However, improper dihedrals are used sparingly in force fields and are difficult to add “by hand”, so, in general, we do not recommend them as a means to attach dummy atoms. Furthermore, our attempts to employ a bond, angle, *improper* dihedral approach turned out to be prone to flapping, quite similarly to a bond, two angles approach if the equilibrium angles are not set to 90°.

We tested the theoretical considerations and practical recommendations following from our analyses by calculating 24 relative solvation free energy differences for 12 pairs of molecules. Specifically, we checked whether the difference of the absolute solvation free energies and the relative solvation free energy difference calculated along the alchemical paths agreed within statistical error bars. Since in the latter case there is no ambiguity how to treat dummy atoms (the noninteracting atoms of the solute), any deviations obtained along the two paths point to the treatment of dummy atoms in the alchemical transformation as the source of error. In most calculations, the two results agreed excellently within very narrow statistical error bars, which were below ±0.1 kcal/mol in all calculations reported in this study. Obviously, this was expected for all calculations in which we followed best practice. In addition, we also obtained good agreement (*p* > 0.05) for terminal and dual junctions in which dummy atoms were treated “naively”, i.e., in which all bonded terms acting on them were kept, even if these redundant terms prevent separability of the partition function. Similarly, for the dual topology system studied, no differences between the best practice and a naive treatment of bonded terms to atoms in the respective noninteracting group could be discerned. By contrast, statistically significant deviations of up to 0.6 kcal/mol between the two paths were found in all cases involving nonplanar triple junctions. In these cases, the naive treatment of the bonded terms involving the dummy atom(s) distorted the average geometry of the physical endpoint, lowering its average dipole moment; hence, the interaction with the solvent was modified. What we refer to as the naive treatment seems to be used frequently in practice. Our results indicate that systematic errors from doing so are often negligible, in particular, if the alchemical transformation corresponds to a terminal or dual junction. Nevertheless, the results for MET2AMM, ETH2AMM, and ETH2MTA serve as a warning that treating dummy atoms incorrectly can lead to erroneous results.

Our analysis and examples cover most alchemical transformations which are likely to arise in practice, with the exception of ring closure/breaking.^46^ Note, however, that in the case of terminal junctions the group transformed to dummy atoms can be cyclic (cf. the toluene to methane example). Our considerations and conclusions apply to any simulation package capable of carrying out alchemical FES. It was stated that “dummy atoms can, in principle, affect free energies, but handled correctly, their effects can often be neglected”.^12^ This study asserts this statement, and we hope that it will serve as the guide on how to handle dummy atoms correctly.

## Supporting Information Available

(1) Outline of derivations needed for results presented in Section 2.1.1, plus a two-dimensional example illustrating how coupling between dummy and physical atoms can be analyzed using the formalism of constraints. (2) Derivation why attaching a dummy atom by a bond and two angles prevents separation of the partition function except in a special case, plus detailed analysis of the energy barrier along different pathways for inversion at a pyramidal center when interactions are described by typical bonded force field terms. (3) Detailed description on how the statistical error was estimated in TI. (4) Pseudocode used for statistical error estimation in the spline-based TI approach used. (5) Detailed specifications for each transformation studied, as well as individual gas phase/solution free energy differences. (6) Table comparing absolute solvation free energies calculated here with experimental values (where available) from the Minnesota Solvation Database (https://doi.org/10.13020/3eks-j059). (7) Figure illustrating the behavior of ⟨∂*U*/∂λ⟩_λ_ for PRP2DIM-2/3. Various analysis tools as well as scripts to (re)create all CHARMM inputs used can be downloaded from Zenodo (https://doi.org/10.5281/zenodo.4381707). (PDF)
