# Supplementary material for: Dummy Atoms in Alchemical Free Energy Calculations
Source: J Chem Theory Comput. 2021 Jun 14;17(7):4403–19. doi: 10.1021/acs.jctc.0c01328 (PMC8280730; doi:10.1021/acs.jctc.0c01328)
Supplement: Supplementary file 1 — ct0c01328_si_001.pdf [file ct0c01328_si_001.pdf]

Dummy atoms in alchemical free energy calculations

Markus Fleck,[†] Marcus Wieder,[‡] and Stefan Boresch[*,†]

†*University of Vienna, Faculty of Chemistry, Department of Computational Biological Chemistry, Währingerstraße 17, A-1090 Vienna, Austria*

‡*Department of Pharmaceutical Chemistry, Faculty of Life Sciences, University of Vienna, Althanstraße 14, 1090 Vienna, Austria*

E-mail: stefan@mdy.univie.ac.at

Contents

1 Dummy atom contributions to the partition function 3

1.1 General considerations 3

1.2 Analysis of coupling between dummy and physical atoms in 2D space 6

2 Details concerning coupled three angles 10

2.1 Additional theoretical details 10

2.2 Energy barrier of inversion at a pyramidal center 12

3 Error analysis in thermodynamic integration 22

4 Pseudo code for statistical error estimation in the spline based thermodynamic integration approach used 25

5 Detailed specifications for each transformation studied 27

5.1 Terminal junctions 29
5.1.1 Hexane–Propane 29
5.1.2 Toluene–Methane 30
5.2 Dual Junctions 31
5.2.1 Ethane–Methanol 31
5.2.2 Methane–Water 32
5.2.3 Toluene–Pyridine 33
5.2.4 Hexane–Propanol 34
5.2.5 Propane–Dimethylether 35
5.3 Triple Junctions 36
5.3.1 Acetone–2-Propenol 36
5.3.2 Phenol–Cyclohexadienone 37
5.3.3 Ethane–Methylamine 38
5.3.4 Methane–Ammonia 39
5.3.5 Ethane–Ammonia 40
5.4 Dual Topology junctions 41
5.4.1 Acetone–2-Propenol dual topology 41

6 Comparison of absolute solvation free energies from simulation with experiment 42

7 Integrand $\langle \frac{\partial U}{\partial \lambda} \rangle$ for PRP2DIM-2/3 44

References 45

1 Dummy atom contributions to the partition function

We first sketch the derivations leading to the starting point of the theoretical considerations of the main manuscript (Sect. 1.1). In addition (Sect. 1.2), we exemplify the effect of redundant bonded terms for a two-dimensional example.

1.1 General considerations

Since we are concerned with classical mechanical systems, the contribution of the kinetic energy to the partition function can always be separated, and its contribution to double free energy differences cancels. We start by exploring under what conditions the configurational partition function Z of a molecule to which dummy atoms are attached, e.g., solutes $\mathrm{L1^D}$ and $\mathrm{L2^D}$ in Fig. 1(b) of the main manuscript, can be written as the product $Z(L^D) = Z(L) \times Z(D)$. L denotes the physical molecule and the superscript D indicates the presence of dummy atoms. Clearly, if the partition function can be written as a product, any dummy atom contribution cancels from the relative free energy differences of interest (cf. Fig. 1 of the main manuscript). If, on the other hand, there is no such separability of the partition function, or, in other words, if there is coupling between the energy terms of the physical system and the (bonded) energy terms involving dummy atoms, the equilibrium geometry as well as the dynamics of the physical atoms may be different in the presence and absence of dummy atoms. The physical atoms interact with the remainder of the system (water, protein etc.); these interactions depend on the (average) geometry of these atoms, as well as their dynamics. In the context of a thermodynamic cycle, these interactions take place in two different environments, e.g., gas phase and aqueous solution (calculation of relative solvation free energies), or in aqueous solution and in the protein (relative binding free energy calculations). Thus, in the presence of coupling, the identity $\Delta A_2 - \Delta A_1 \neq \Delta A'_2 - \Delta A'_1$ (cf. Fig. 1 of the main manuscript) may not hold.

We start with the potential energy function of some molecule $\mathrm{L^D}$ in which one or more

dummy atoms are present. Depending on the application our molecule could be in gas phase, aqueous solution or bound to a protein, we will refer to this as environment (E). The potential energy function can be schematically written as

$$U_{L^D} = U_{LE}(\mathbf{r}_{\mathrm{L}}, \mathbf{r}_{\mathrm{E}}) + U_D(\mathbf{b}_{\mathrm{LD}}(\mathbf{r}_{\mathrm{L}}, \mathbf{r}_{\mathrm{D}}), \mathbf{b}_{\mathrm{D}}(\mathbf{r}_{\mathrm{D}})) \tag{1}$$

In Eq. 1 the term U_{LE} encompasses all bonded and non-bonded interactions within the physical molecule and the non-bonded interactions between L and E. The second term U_D comprises all interactions in which dummy atoms participate. By employing the notation $\mathbf{b}_{\mathrm{LD}}(\mathbf{r}_{\mathrm{L}}, \mathbf{r}_{\mathrm{D}})$ and $\mathbf{b}_{\mathrm{D}}(\mathbf{r}_{\mathrm{D}})$, we emphasize that dummy atoms interact only through bonded terms. Since $\mathbf{r}_{\mathrm{L}}$ appears in both terms on the right hand side, no separation of the configurational partition function is possible in Cartesian coordinates.

The desired factorization can be accomplished by a partial change of variables from Cartesian to *suitable* internal coordinates $\{\mathbf{b}'_{\mathrm{LD}}, \mathbf{b}'_{\mathrm{D}}\}$. Adapting steps outlined, e.g., in Refs. 1–4, we obtain

$$Z = \int d\mathbf{r}_{\mathrm{E}} d\mathbf{r}_{\mathrm{L}} \; \exp(-\beta U_{LE}(\mathbf{r}_{\mathrm{L}}, \mathbf{r}_{\mathrm{E}})) \int d\mathbf{b}'_{\mathrm{LD}} d\mathbf{b}'_{\mathrm{D}} \; |\hat{J}_{\mathrm{LD,D}}(\mathbf{b}'_{\mathrm{LD}}, \mathbf{b}'_{\mathrm{D}})| \; \exp(-\beta U_D(\mathbf{b}'_{\mathrm{LD}}, \mathbf{b}'_{\mathrm{D}})) \tag{2}$$

Here $|\hat{J}_{\mathrm{LD,D}}(\mathbf{b}'_{\mathrm{LD}}, \mathbf{b}'_{\mathrm{D}})|$ is the Jacobian resulting from the coordinate transformation. It is *independent* of $\mathbf{r}_{\mathrm{E}}$ and $\mathbf{r}_{\mathrm{L}}$ because of the choice of $\{\mathbf{b}'_{\mathrm{LD}}, \mathbf{b}'_{\mathrm{D}}\}$ defining the position of the dummy atoms *relative* to the coordinates of the physical atoms. Eq. 2 has the desired form

$$Z = Z_{LE}(\mathbf{r}_{\mathrm{L}}, \mathbf{r}_{\mathrm{E}}) \times Z_D(\mathbf{b}'_{\mathrm{LD}}, \mathbf{b}'_{\mathrm{D}}) \tag{3}$$

However, the factorization of Eq. 2 into a term for the physical system and a term comprising the dummy atom contributions depends on several prerequisites. In general, the steps leading to Eq. 2 assume that one uses exactly *three* internal degrees of freedom and associated force field terms per (dummy) atom;[1–4] Herschbach, Johnston and Rapp refer to

these as *non-redundant* degrees of freedom.[1] E.g., given four non-linear, unbranched atoms, for which the Cartesian coordinates of atoms 1–3 are known, the position of atom 4 can be specified in terms of the bond distance d_{43}, the angle θ_{432} and the dihedral angle φ_{4321}. Any additional force field term acting on 4 constitutes a *redundant* degree of freedom. As already mentioned, in modern force fields an energy term is assigned to each valence and dihedral angle formed by the covalent bonds; this means that there are usually more than three bonded energy terms acting on an atom. Thus, in most practical cases the three non-redundant internal coordinates $\{\mathbf{b}'_{\mathrm{LD}}, \mathbf{b}'_{\mathrm{D}}\}$ employed in the coordinate transformation leading to Eq. 2 are a subset of the bonded energy terms present. Consequently, if widespread practice is followed and the bonded energy terms attaching a dummy atom to a physical atom are the same as in the corresponding native state, then the position and orientation of the dummy atom relative to the physical system depends on (many) more than three degrees of freedom (bonded energy terms).

When investigating how the presence of such redundant terms affects the separability of the partition function, one has to distinguish two cases. Any redundant degrees of freedom (bonded energy terms), which depend only on positions (coordinates) of dummy atoms, are of no concern; their contribution to the partition function can be factored even in Cartesian coordinates. In fact, within large groups of dummy atoms all bonded interactions should be kept to maintain its structural integrity. By contrast, care is required in the "junction region", i.e., for redundant bonded terms, which involve coordinates of physical atoms as well as dummy atoms. It is these cases we will analyze in the following subsections.

In biomolecular simulations bonds are frequently held rigid by holonomic constraints to increase the integration time-step. As will be shown in the following subsections, complications from non-redundant degrees of freedom result from angle bending or dihedral angle terms, which are not subject to constraints. Therefore, all considerations given below apply regardless whether bonds are flexible or held rigid by SHAKE,[5] RATTLE[6] or similar means.

1.2 Analysis of coupling between dummy and physical atoms in 2D space

Keeping non-redundant bonded energy terms in the junction region may be convenient as one does not have to choose exactly three bonded terms, and it may be helpful or even needed to prevent flapping (cf. Sect. 2.2 of the main manuscript). To illustrate how we investigate whether/when doing so affects the separability of the partition function Eq. 3, we discuss here a two-dimensional example. As a model, we consider the alchemical transformation from ethene (ETH) to water, specifically, the water end point with three dummy atoms attached (WAT-D) depicted on the right hand side of Fig. 1. When the transmutation

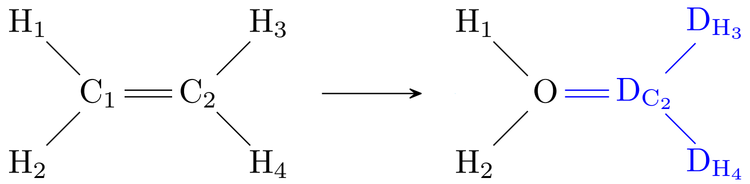

Figure 1: Schematic representation of the alchemical transformation from ethene (ETH) to water (with dummy atoms, WAT-D). Both endpoints are considered to be planar.

to water is completed, no intramolecular non-bonded interactions are present. Since we assume the molecules to be perfectly planar, the only force field terms needed are the usual quadratic bond stretching and angle bending terms, $k(x-x_0)^2 = k(\Delta x)^2$, where Δx denotes the displacement of a bond length r or an angle θ from its equilibrium value.

In 2D there are $2N-3$ non-redundant intramolecular degrees of freedom, i.e., in our example (six atoms, three physical, three dummy atoms) there are 9 non-redundant degrees of freedom. We start with the potential energy functions of physical water and the dummy

part, respectively,

$$\begin{aligned}
U_{wat} &= k_{\text{O-H}_1}(\Delta r_{\text{O-H}_1})^2 + k_{\text{O-H}_2}(\Delta r_{\text{O-H}_2})^2 \\
&+ k_{\text{H}_1\text{-O-H}_2}(\Delta\theta_{\text{H}_1\text{-O-H}_2})^2 \\
U_{pure\ dummy} &= k_{\text{D}_{\text{C}_2}\text{-D}_{\text{H}_3}}(\Delta r_{\text{D}_{\text{C}_2}\text{-D}_{\text{H}_3}})^2 + k_{\text{D}_{\text{C}_2}\text{-D}_{\text{H}_4}}(\Delta r_{\text{D}_{\text{C}_2}\text{-D}_{\text{H}_4}})^2 \\
&+ k_{\text{D}_{\text{H}_3}\text{-D}_{\text{C}_2}\text{-D}_{\text{H}_4}}(\Delta\theta_{\text{D}_{\text{H}_3}\text{-D}_{\text{C}_2}\text{-D}_{\text{H}_4}})^2 \qquad (4)
\end{aligned}$$

where the subscripts refer to the atom labels of Fig. 1. Having used 6 out of 9 non-redundant intramolecular degrees of freedom, we have available three energy terms to attach the dummy atoms to the physical water part. Specifically, we anchor D_{C_2} relative to water and O relative to the dummy part. In 2D, in addition to the bond $r_{\text{O-D}_{\text{C}_2}}$, one angle per side is sufficient; no dihedral angle is needed:

$$U_{junction} = k_{\text{O-D}_{\text{C}_2}}(\Delta r_{\text{O-D}_{\text{C}_2}})^2 + k_{\text{H}_1\text{-O-D}_{\text{C}_2}}(\Delta\theta_{\text{H}_1\text{-O-D}_{\text{C}_2}})^2 + k_{\text{O-D}_{\text{C}_2}\text{-D}_{\text{H}_3}}(\Delta\theta_{\text{O-D}_{\text{C}_2}\text{-D}_{\text{H}_3}})^2 \qquad (5)$$

Thus, using Eqs. 4 and 5 the full energy function for the WAT-D endpoint is

$$U_{WAT-D} = U_{wat} + \underbrace{U_{junction} + U_{pure\ dummy}}_{U_{dummy}} \qquad (6)$$

To illustrate the steps leading to Eq. 2 in the general treatment, we use Eq. 6 and explicitly write the partition function for the WAT-D end state

$$\begin{aligned}
Z_{\text{WAT-D}} &= \left[\int d\mathbf{r}_{\text{O}} d\mathbf{r}_{\text{H}_1} d\mathbf{r}_{\text{H}_2} \exp(-\beta U_{wat})\right] \times \\
&\left[\int_0^\infty \mathrm{d}r_{\text{O-D}_{\text{C}_2}}\, r_{\text{O-D}_{\text{C}_2}} \int_0^{2\pi} \mathrm{d}\theta_{\text{H}_1\text{-O-D}_{\text{C}_2}} \int_0^\infty \mathrm{d}r_{\text{D}_{\text{C}_2}\text{-D}_{\text{H}_3}}\, r_{\text{D}_{\text{C}_2}\text{-D}_{\text{H}_3}} \int_0^{2\pi} \mathrm{d}\theta_{\text{O-D}_{\text{C}_2}\text{-D}_{\text{H}_3}}\right. \\
&\left.\int_0^\infty \mathrm{d}r_{\text{D}_{\text{C}_2}\text{-D}_{\text{H}_4}}\, r_{\text{D}_{\text{C}_2}\text{-D}_{\text{H}_4}} \int_0^{2\pi} \mathrm{d}\theta_{\text{D}_{\text{H}_3}\text{-D}_{\text{C}_2}\text{-D}_{\text{H}_4}} \exp(-\beta U_{dummy})\right] \\
&= Z_{wat} \times Z_{dummy} \qquad (7)
\end{aligned}$$

where the square brackets indicated the two multiplicative factors. Cartesian coordinates are kept for the physical system (water), but for the dummy part of WAT-D we transform to the internal coordinates present in the bonded energy terms of Eq. 6. The Jacobian factors $r_{\text{A-B}}$ resulting from the transformation from Cartesian to planar polar coordinates positioning atom B relative to atom A are written explicitly; one sees that they do not depend on degrees of freedom of the physical water molecules.

At the corresponding ETH endpoint, two additional angle bending terms, $k_{\text{H}_2\text{-O-D}_{\text{C}_2}}(\Delta\theta_{\text{H}_2\text{-O-D}_{\text{C}_2}})^2$ and $k_{\text{D}_{\text{H}_4}\text{-D}_{\text{C}_2}\text{-O}}(\Delta\theta_{\text{D}_{\text{H}_4}\text{-D}_{\text{C}_2}\text{-O}})^2$, are present. We now investigate the effect of including either of these redundant terms on the separability of the partition function at the WAT-D endpoint. Let us start with the angle term in $\theta_{\text{D}_{\text{H}_4}\text{-D}_{\text{C}_2}\text{-O}}$. In the general notation of Eq. 2, it is not part of the non-redundant degrees of freedom $\{\mathbf{b}'_{\text{LD}}, \mathbf{b}'_{\text{D}}\}$, i.e., it is not one of the integration variables in the configurational integral Eq. 7. Therefore, we need to express it in terms of the non-redundant internal coordinates chosen earlier, $r_{\text{O-D}_{\text{C}_2}}$, $\theta_{\text{H}_1\text{-O-D}_{\text{C}_2}}$, $\theta_{\text{D}_{\text{H}_3}\text{-D}_{\text{C}_2}\text{-O}}$, $r_{\text{D}_{\text{C}_2}\text{-D}_{\text{H}_3}}$, $r_{\text{D}_{\text{C}_2}\text{-D}_{\text{H}_4}}$ and $\theta_{\text{D}_{\text{H}_3}\text{-D}_{\text{C}_2}\text{-D}_{\text{H}_4}}$. Adding a redundant degree of freedom is equivalent to introducing a geometrical constraint, which in this specific case is given by

$$\theta_{\text{D}_{\text{H}_3}\text{-D}_{\text{C}_2}\text{-D}_{\text{H}_4}} + \theta_{\text{D}_{\text{H}_3}\text{-D}_{\text{C}_2}\text{-O}} + \theta_{\text{D}_{\text{H}_4}\text{-D}_{\text{C}_2}\text{-O}} = 2\pi. \tag{8}$$

Adding $k_{\text{D}_{\text{H}_4}\text{-D}_{\text{C}_2}\text{-O}}(\Delta\theta_{\text{D}_{\text{H}_4}\text{-D}_{\text{C}_2}\text{-O}})^2$ to U_{WAT-D} (Eq. 6) and utilizing Eq. 8, we obtain for the potential energy function of WAT-D

$$\begin{aligned} &U_{wat} + U_{dummy} + k_{\text{D}_{\text{H}_4}\text{-D}_{\text{C}_2}\text{-O}}(\Delta\theta_{\text{D}_{\text{H}_4}\text{-D}_{\text{C}_2}\text{-O}})^2 = \\ &\qquad U_{wat} + U_{dummy} + k_{\text{D}_{\text{H}_4}\text{-D}_{\text{C}_2}\text{-O}}\left[\left(2\pi - \theta_{\text{D}_{\text{H}_3}\text{-D}_{\text{C}_2}\text{-D}_{\text{H}_4}} - \theta_{\text{D}_{\text{H}_3}\text{-D}_{\text{C}_2}\text{-O}}\right) - \theta^0_{\text{D}_{\text{H}_4}\text{-D}_{\text{C}_2}\text{-O}}\right]^2 \end{aligned} \tag{9}$$

On the right hand side of Eq. 9 the redundant energy term has been expressed in terms of non-redundant degrees of freedom. The superscript 0 indicates the equilibrium value of the additional force-field term. Its instantaneous value depends on $\theta_{\text{D}_{\text{H}_3}\text{-D}_{\text{C}_2}\text{-D}_{\text{H}_4}}$ and

$\theta_{\mathrm{D_{H_3}\text{-}D_{C_2}\text{-}O}}$, all of which are internal coordinates with respect to dummy atoms. Thus, no coupling with the physical degrees of freedom (water) is introduced and the partition function of the WAT-D endpoint following from Eq. 9 remains separable. While the additional $k_{\mathrm{D_{H_4}\text{-}D_{C_2}\text{-}O}}(\Delta\theta_{\mathrm{D_{H_4}\text{-}D_{C_2}\text{-}O}})^2$ term will affect single free energy differences, it will cancel exactly from double free energy differences between two environments and can safely be included as a force-field term to keep the dummy atoms attached to the physical system. An important observation here is that this can be deduced directly from the constraint equation Eq. 8, which does *not* contain any terms involving degrees of freedom of the *physical molecule*. Explicitly writing expressions for the potential energy function, such as Eq. 9 above, is not needed, which simplifies analyses considerably.

Let us repeat the above steps for the second redundant degree of freedom, the $\theta_{\mathrm{H_2\text{-}O\text{-}D_{C_2}}}$ angle, one would potentially like to keep. In this case, the relevant geometrical constraint reads

$$\theta_{\mathbf{H_1\text{-}O\text{-}H_2}} + \theta_{\mathrm{H_1\text{-}O\text{-}D_{C_2}}} + \theta_{\mathrm{H_2\text{-}O\text{-}D_{C_2}}} = 2\pi. \qquad (10)$$

Eq. 10 involves $\theta_{\mathrm{H_1\text{-}O\text{-}H_2}}$ (emphasized in boldface), an internal coordinate of the physical water molecule. While one can express the redundant energy term $k_{\mathrm{H_2\text{-}O\text{-}D_{C_2}}}(\Delta\theta_{\mathrm{H_2\text{-}O\text{-}D_{C_2}}})^2$ in terms of the non-redundant degrees of freedom used in Eqs. 4 and 5, in analogy to the steps leading to Eq. 9, the resulting configurational integral is not separable anymore; in other words, the desired factorization Eq. 3 is no longer possible. This can be deduced immediately from the simultaneous presence of physical and dummy degrees of freedom in the constraint Eq. 10.

To summarize, the addition of an energy term in a redundant internal degree of freedom introduces a geometric constraint. If the constraint involves only internal degrees of freedom pertaining to one or more dummy atoms, then the physical part of the system is not affected, and any contribution, which the additional force field term makes to the partition function, cancels from double free energy differences. If, on the other hand the constraint couples the redundant degree of freedom with one or more physical degrees of freedom, then the physical

system is affected and the partition function cannot be factorized.

2 Details concerning coupled three angles

2.1 Additional theoretical details

We outlined in the main manuscript ("Coupled three angles" in Sect. 2.1.2) that unintended coupling between degrees of freedom of dummy atoms and physical atoms arises when there are three atoms bound to a central atom, and one of these is transformed into a dummy atom. The simplest possible model for this is the alchemical transformation of ammonia to water, cf. Fig. 5 of the main manuscript. We showed that the following constraint applies to the physical bond angle

$$|\theta_{\text{D-O-H}_1} - \theta_{\text{D-O-H}_2}| \;\leq\; \theta_{\text{H}_1\text{-O-H}_2} \;\leq\; M(\theta_{\text{D-O-H}_1}, \theta_{\text{D-O-H}_2}) \tag{11}$$

where we use the shorthand

$$M(\theta_{\text{D-O-H}_1}, \theta_{\text{D-O-H}_2}) \equiv \min(\theta_{\text{D-O-H}_1} + \theta_{\text{D-O-H}_2},\; 2\pi - \theta_{\text{D-O-H}_1} - \theta_{\text{D-O-H}_2})$$

The upper and lower bounds in Eq. 11 both arise in the limiting case of all four atoms being coplanar. The lower bound can be deduced immediately from Figs. 2(d,e) with $\theta_2 = \theta_{\text{H}_1\text{-O-H}_2}$ (see 2.2); similarly, the upper bound follows from Figs. 2(b,c) with $\theta_3^{(\prime)} = \theta_{\text{H}_1\text{-O-H}_2}$ Using the methodology of Herschbach, Johnston and Rapp (see their Table I),[1] the partition function of the water end state with the dummy atom attached can be written as

$$\begin{aligned} Z \;=\; & V 8\pi^2 \int_0^\infty \mathrm{d}r_{\text{D-O}}\, r_{\text{D-O}}^2 \int_0^\infty \mathrm{d}r_{\text{O-H}_1}\, r_{\text{O-H}_1}^2 \int_0^\pi \mathrm{d}\theta_{\text{D-O-H}_1}\, \sin(\theta_{\text{D-O-H}_1}) \int_0^\infty \mathrm{d}r_{\text{O-H}_2}\, r_{\text{O-H}_2}^2 \\ & \times \int_0^\pi \mathrm{d}\theta_{\text{D-O-H}_2} \int_{|\theta_{\text{D-O-H}_1} - \theta_{\text{D-O-H}_2}|}^{M(\theta_{\text{D-O-H}_1}, \theta_{\text{D-O-H}_2})} \mathrm{d}\theta_{\text{H}_1\text{-O-H}_2}\, \sin(\Psi)^{-1} \exp(-U/kT) \end{aligned} \tag{12}$$

with

$$\sin(\Psi) = \sqrt{\frac{1-\cos(\theta_{\text{D-O-H}_1})^2-\cos(\theta_{\text{D-O-H}_2})^2-\cos(\theta_{\text{H}_1\text{-O-H}_2})^2-2\cos(\theta_{\text{D-O-H}_1})\cos(\theta_{\text{D-O-H}_2})\cos(\theta_{\text{H}_1\text{-O-H}_2})}{\sin(\theta_{\text{H}_1\text{-O-H}_2})^2\sin(\theta_{\text{D-O-H}_2})^2}}.$$

The potential energy function U consists of all bonded energy terms present in water with the dummy atom attached to it. Since our integration variables are exactly the internal coordinates of these energy term, which are additive, the potential energy function U itself in this non-redundant coordinate set is unproblematic. However, the partition function Eq. 12 does not factorize because of the limits of integration and the functional form of the Jacobian factor $\sin\Psi$, which couples all three angles.

Thus, attaching a dummy atom by a bond and two angles does *not* lead to a multiplicative contribution to the partition function. Two limiting cases, however, are of special interest. If one attempts to keep all atoms, including the dummy atom, in the same plane, the hard constraint $\theta_{\text{H}_1\text{-O-H}_2} + \theta_{\text{H}_1\text{-O-D}} + \theta_{\text{H}_2\text{-O-D}} = 2\pi$ applies, and an influence of the dummy atom on the physical water molecule cannot be avoided. In fact, this is the limit case with the strongest coupling. If, on the other hand, one chooses $\theta_{\text{D-O-H}_1} = \theta_{\text{D-O-H}_2} = \pi/2$, then the limits of integration reduce to

$$\begin{aligned} |\theta_{\text{D-O-H}_1} - \theta_{\text{D-O-H}_2}| &= 0 \\ M(\theta_{\text{D-O-H}_1}, \theta_{\text{D-O-H}_2}) &= \pi \end{aligned} \tag{13}$$

and

$$\sin(\Psi) = 1 \tag{14}$$

With these simplifications Eq. 12 can be rewritten so that the partition function of the physical water molecule becomes a multiplicative factor, i.e., the desired separability of the partition function is regained. The somewhat surprising conclusion from this analysis is that

whenever one anchors a dummy atom by one bond and two angles with respect to a physical molecule, one should set the equilibrium values of the two angles involving the dummy atom to 90 degrees in order to maintain separability of the partition function (Eq. 3), at least in good approximation since the dummy angles still fluctuate around 90 degrees. As shown next, this choice for the bond angles involving the dummy atom also effectively prevents flapping.

2.2 Energy barrier of inversion at a pyramidal center

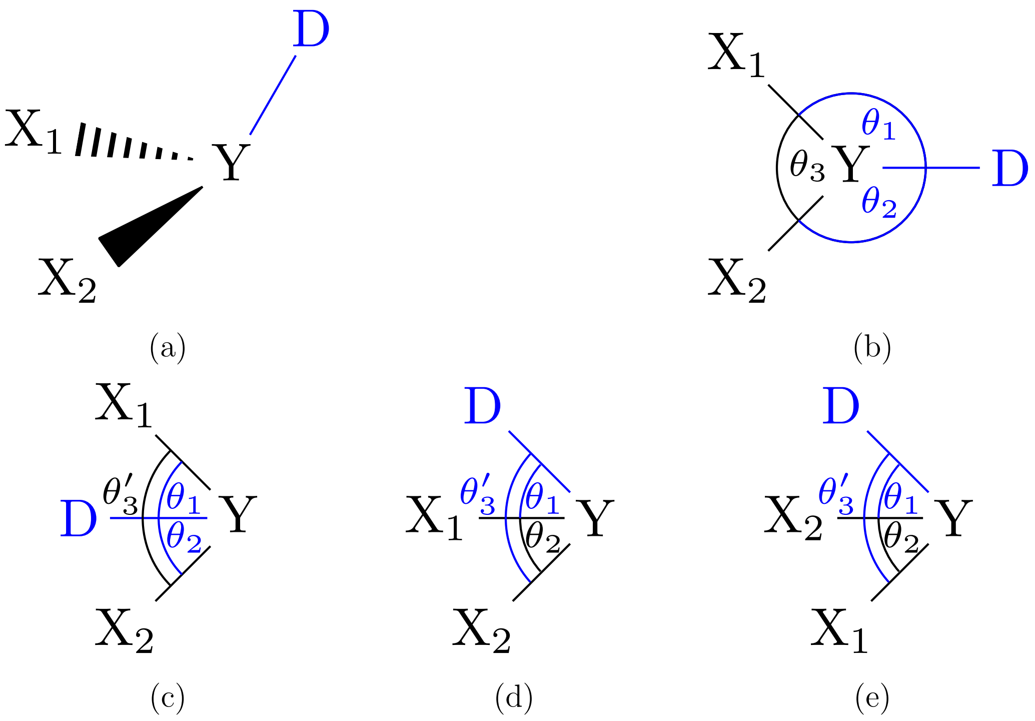

Figure 2: Possible paths of pyramidal flapping. (a) Dual junction in ammonia-like sp^3 geometry. (b) Symmetric pathway: Y flaps through the midpoint of X_1, X_2 and D or, equivalently, D crosses the X_1-Y-X_2 plane following a path distant to X_1 and X_2. (c) Canonical asymmetric path: D takes the path between X_1 and X_2 to cross the X_1-Y-X_2 plane. The dash in θ_3' indicates that it is chosen as $0 \leq \theta_3' \leq \pi$, so $\theta_1 + \theta_2 + \theta_3' \neq 2\pi$. For usual geometries encountered in chemistry, θ_1 and θ_2 are acute angles (in contrast to case (b)), i.e., $0 \leq \theta_1, \theta_2 \leq \pi/2$. If unusual geometries are permitted, one (but only one) of them may be obtuse. (d) Non-canonical asymmetric path I: X_1 takes the path between D and X_2 to cross the D-Y-X_2 plane. (e) Non-canonical asymmetric path II: same as (c) and (d), but X_2 takes the path between D and X_1 to cross the D-Y-X_1 plane.

We just showed that attaching a dummy atom through a bond stretching and two bond angle terms to a physical system leads to a three angle constraint (two dummy angles, one physical angle), which potentially influences the physical system. Somewhat unexpectedly, the effect of this constraint can be (mostly) avoided by setting the equilibrium value of the two angle terms involving the dummy atom to 90°. In Sect. 2.2 of the main manuscript we showed that positioning a dummy atom relative to the physical system through a bond stretching and two bond angle terms is prone to flapping, as this choice of internal coordinates leads to two sets of Cartesian coordinates, which are equivalent with respect to their internal coordinates for the dummy atom. In the main manuscript, our example was the alchemical transformation of ammonia to water, with one of the ammonia hydrogens becoming a dummy atom attached to the water oxygen. This dummy atom can be either "above" or "below" the plane formed by the water atoms. When the dummy atom switches between the two possible positions, it has to traverse this plane, i.e., all four atoms are in the same plane, exactly the configuration for which the effect of the three angle constraint is most pronounced.

While the ammonia to water transformation may be considered a fringe case, positioning a dummy atom or group relative to a physical system through one bond and two bond angles is an appealing option. It is, therefore, necessary to understand the pathway of flapping which may arise in this case. Special emphasis has to be paid to the case where the equilibrium angles involving dummy atoms have been set to 90° to mitigate the effect of the three angle constraint. Further, at the ammonia end state, "flapping" is normal since this is the nitrogen inversion occurring for ammonia and amines. Conversely, at the water end state (assuming a flexible water molecule), we would like to keep the dummy atom in a fixed position relative to the water molecule to prevent the planar transition state, which through a three angle constraint would influence the angle bending motion of water.

Fig. 2(a) shows the most general form of attaching a dummy atom D to a branched physical system X_1-Y-X_2. In the ammonia to water transformation, this reduces to H-O-H. This configuration arises in other model transformations considered in the main manuscript.

For the toluene to pyridine transformation one has (see Fig. 9) $D_C \hat{=} D$, $N \hat{=} Y$, $C_1 \hat{=} X_1$ and $C_5 \hat{=} X_2$. Similarly, for acetone to 2-propenol (see Fig. 12), the correspondence is $D_{H_{13}} \hat{=} D$, $C_1 \hat{=} Y$, $C_2 \hat{=} X_1$ and $H_{11} \hat{=} X_2$.

Figs. 2(b)–(e) illustrate the four possible pathways, along which "pyramidal flapping" may occur. All involve a configuration in which the four atoms are coplanar. Fig. 2(b) shows the symmetric path, where D passes through the X_1-Y-X_2 plane while staying at the opposite side of atoms X_1 and X_2. Alternatively, this path can be viewed as the central atom Y tunneling through the plane formed by X_1, X_2 and D. This latter view, of course, corresponds to nitrogen inversion. While this is the expected pathway for normal molecular geometries, we have to keep two aspects in mind. First, we are discussing dummy atom dynamics here, which we want to keep in a well defined position with respect to a physical molecule. Second, because we want to mitigate the effect of the associated three angle constraint on the physical system, we also have to consider the unusual case where angles X_i-Y-D are set to 90°.

The first possible asymmetric path is illustrated in Fig. 2(c): D crosses the plane X_1-Y-X_2 between X_1 and X_2. We refer to it as canonical asymmetric, as it is the dummy atom which crosses the plane. The second asymmetric path, shown in Fig. 2(d), is obtained if X_1 crosses the D-Y-X_2 plane between D and X_2. In the third asymmetric path (Fig. 2(e)), X_2 crosses the D-Y-X_1 plane between D and X_1.

To discriminate between these four potential pathways, we need to calculate the minimum energy of the planar configurations depicted in Figs. 2(b)–(e), which correspond to the respective transition state. The height of the energy barrier a particle has to cross along the four pathways also indicates how likely it is that flapping will occur. We start with the symmetric planar configuration shown in Fig. 2(b) and carry out an energy minimization under the constraint of planarity, i.e., the sum of the three angles has to equal 2π. The only energy terms we need to consider are the three angle bending terms; changes in bond lengths do not affect the angles. Due to non-bonded exclusions, there are neither Lennard-Jones, nor

electrostatic interactions present between the physical atoms X_1, X_2, Y, and obviously none for D. Assuming quadratic potentials, the potential energy term including the Lagrangian multiplier is:

$$\mathcal{L} = \sum_{j=1}^{3} (\theta_j - \theta_j^0)^2 k_j - \lambda(\sum_{j=1}^{3} \theta_j - 2\pi) \tag{15}$$

We want to find the angles θ_i; θ_i^0 and k_i are the equilibrium angles and force constant of the respective force field terms. Taking the derivatives and setting them to zero leads to the following system of equations

$$\begin{aligned} \frac{\partial \mathcal{L}}{\partial \theta_i} &= 2(\theta_i - \theta_i^0)k_i - \lambda = 0 \qquad i = 1, 2, 3 \\ \frac{\partial \mathcal{L}}{\partial \lambda} &= \sum_{j=1}^{3} \theta_j - 2\pi = 0, \end{aligned} \tag{16}$$

with solutions

$$\begin{aligned} \theta_i &= \theta_i^0 + \frac{\lambda}{2k_i} \qquad i = 1, 2, 3 \\ 2\pi &= \sum_{j=1}^{3} \theta_j = \sum_{j=1}^{3} \theta_j^0 + \lambda \frac{\sum_{j<l} k_j k_l}{2 \prod_{j=1}^{3} k_j} \\ \lambda &= \frac{2(2\pi - \sum_{j=1}^{3} \theta_j^0) \prod_{j=1}^{3} k_j}{\sum_{j<l} k_j k_l} \\ \theta_i &= \theta_i^0 + \frac{(2\pi - \sum_{j=1}^{3} \theta_j^0) \prod_{j=1}^{3} k_j}{k_i \sum_{j<l} k_j k_l}. \end{aligned} \tag{17}$$

To find the energy of the transition state, we insert the expressions for the three angles into the energy term of the Lagrangian multiplier equation and obtain

$$E = \sum_{j=1}^{3} (\theta_j - \theta_j^0)^2 k_j = \sum_j \Big(\frac{(2\pi - \sum_{l=1}^{3} \theta_l^0) \prod_{l=1}^{3} k_l}{k_j \sum_{l<m} k_l k_m} \Big)^2 k_j = \Big(\frac{(2\pi - \sum_{l=1}^{3} \theta_l^0) \prod_{l=1}^{3} k_l}{\sum_{l<m} k_l k_m} \Big)^2 \sum_j \frac{1}{k_j}. \tag{18}$$

Next, we turn to the asymmetric cases Figs. 2(c)–(e). Because of our choice of labeling, the same derivation can be applied to all three cases. Here, the Lagrangian multiplier

equation is:

$$\mathcal{L} = \sum_{j=1}^{3}(\theta'_j - \theta^0_j)^2 k_j - \lambda(\sum_{j=1}^{3}\theta_j - 2\pi)$$
$$\theta'_i = \begin{cases} \theta_i & \text{for } 0 \leq \theta_i \leq \pi \\ 2\pi - \theta_i, & \text{for } \pi < \theta_i < 2\pi \end{cases} \tag{19}$$

Because of the energy function for angle bending terms, we need to introduce the auxiliary variables θ'_i. Specifically, the instantaneous value of an angle in an angle bending energy term is expected to be in the range $0 \leq \theta_i \leq \pi$. However, to define the constraint for the coplanar configuration of interest, we also need to allow for angles $\pi < \theta_i < 2\pi$. To proceed, we assume that

$$0 \leq \theta_{1,2} \leq \pi$$
$$\pi < \theta_3 < 2\pi \tag{20}$$

Importantly, θ_3 is always the angle formed by the two atoms (plus central atom Y), between which the respective third atom crosses the plane (θ'_3 is its complement to 2π). E.g., in Fig. 2(c), θ_3 describes the angle formed by atoms X_1 and X_2 (with Y as center), and atom D crosses the X_1-Y-X_2 plane between them. In Fig. 2(d), X_1 crosses the D-Y-X_2 plane between atoms D and X_2 etc. Using this notation, the system of equations to solve is:

$$\frac{\partial \mathcal{L}}{\partial \theta'_i} = 2(\theta'_i - \theta^0_i)k_i - \lambda = 0 \qquad i = 1, 2$$
$$\frac{\partial \mathcal{L}}{\partial \theta'_3} = 2(\theta'_3 - \theta^0_3)k_3 + \lambda = 0$$
$$\frac{\partial \mathcal{L}}{\partial \lambda} = \sum_{j=1}^{3}\theta_j - 2\pi = 0, \tag{21}$$

which leads to

$$\begin{aligned}
\theta'_i &= \theta_i^0 + \frac{\lambda}{2k_i} \qquad \theta_i = \theta'_i \qquad\qquad i = 1,2 && (22)\\
\theta'_3 &= \theta_3^0 - \frac{\lambda}{2k_3} \qquad \theta_3 = 2\pi - \theta'_3 = 2\pi - \theta_3^0 + \frac{\lambda}{2k_3} && (23)\\
2\pi &= \sum_{j=1}^{3} \theta_j = 2\pi + \theta_1^0 + \theta_2^0 - \theta_3^0 + \lambda \frac{\sum_{j<l} k_j k_l}{2\prod_{j=1}^{3} k_j}\\
\lambda &= \frac{2(\theta_3^0 - \theta_2^0 - \theta_1^0)\prod_{j=1}^{3} k_j}{\sum_{j<l} k_j k_l}\\
\theta'_i &= \theta_i^0 + \frac{(\theta_3^0 - \theta_2^0 - \theta_1^0)\prod_{j=1}^{3} k_j}{k_i \sum_{j<l} k_j k_l} \qquad\qquad i = 1,2\\
\theta'_3 &= \theta_3^0 - \frac{(\theta_3^0 - \theta_2^0 - \theta_1^0)\prod_{j=1}^{3} k_j}{k_3 \sum_{j<l} k_j k_l}.
\end{aligned} \qquad (24)$$

Having found the θ', we can insert them in the energy function of the Lagrangian multiplier equation and obtain

$$E = \sum_{j=1}^{3} (\theta'_j - \theta_j^0)^2 k_j = \Big(\frac{(\theta_3^0 - \theta_2^0 - \theta_1^0)\prod_{j=1}^{3} k_j}{\sum_{j<l} k_j k_l}\Big)^2 \sum_{j=1}^{3} \frac{1}{k_j}. \qquad (25)$$

Having derived expressions for the (effective) energy barriers along the four possible paths, we can explore their dependence on the choice of equilibrium angles and force constants. All of the following examples are inspired by the alchemical transformation of ammonia to water. Specifically, if we set $\theta_1^0 = \theta_2^0 = \theta_3^0 = 107.1$ deg and $k_1 = k_2 = k_3 = 41.50$ kcal mol^{-1} rad^{-2}, the force field parameters for ammonia taken from the CHARMM general force field,[7–9] the symmetric energy barrier is 6.3 kcal mol^{-1} and all asymmetric energy barriers equal 48.3 kcal mol^{-1}.

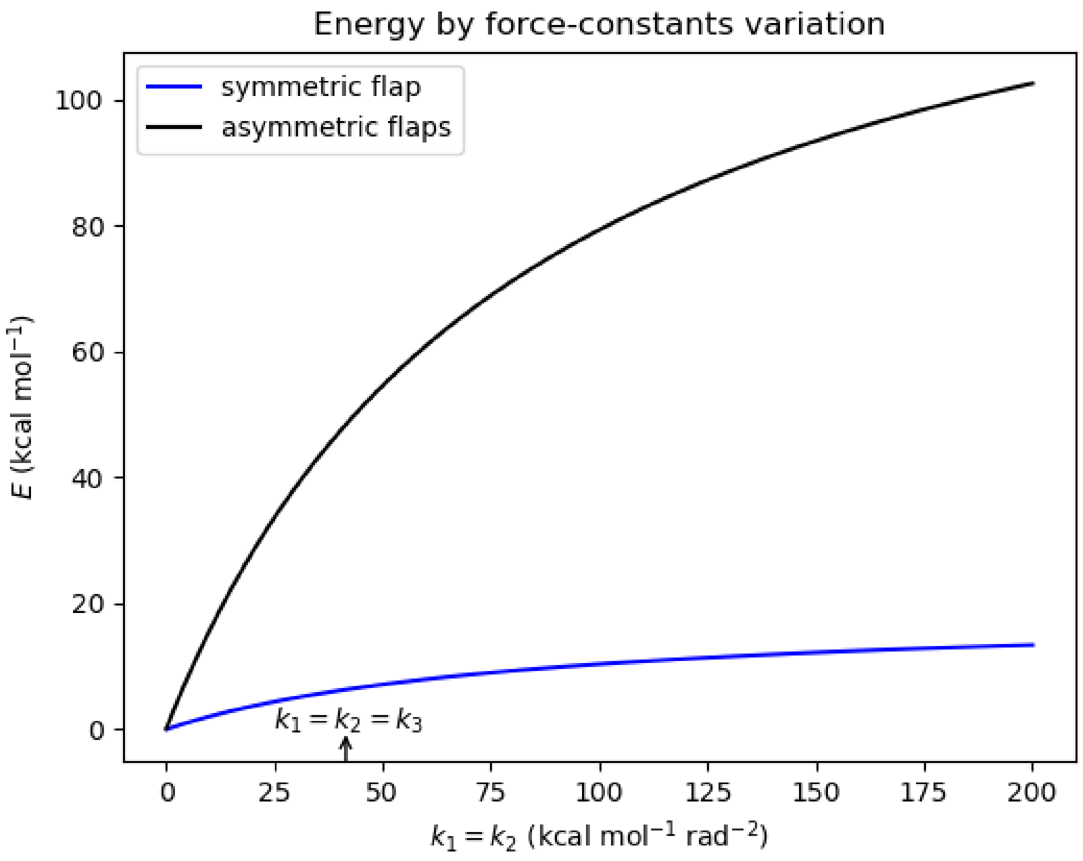

Figure 3: Dependence of the energy barriers for a pyramidal flap on the dummy angle force constants. $\theta_1^0 = \theta_2^0 = \theta_3^0 = 107.1$ deg and $k_3 = 41.50$ kcal mol^{-1} rad^{-2}. The energy barrier of the asymmetric flaps is considerably higher. Index 3 is tied to the physical angle.

Fig. 3 shows how the respective barrier heights change as a function of the force constants k_1 and k_2 for the two angles involving the the dummy atom. The ammonia-like case $k_1 = k_2 = k_3 = 41.50$ kcal mol^{-1} rad^{-2} is indicated by the small arrow. The three asymmetric flaps all have the same energy barrier (minimum energy of the coplanar configuration). This follows from the observation that the minimum energy for all three asymmetric flaps can be obtained by permuting indices 1, 2 and 3 in Eq. 25, while keeping the association of the force-field equilibrium angles θ^0 and force-constants k fixed. E.g., index 3 always refers to the physical angle degree of freedom. Doing so is not in line with the labeling used in Figs. 2(d,e), the utility of which was mostly unification of the mathematical treatment. As

expected, the symmetric flap, corresponding to nitrogen inversion in the case of ammonia, has a far lower energy barrier than the hypothetical asymmetric paths. One sees in Fig. 3 that the height of this barrier depends only weakly on the value of the force constants k_1 and k_2, i.e., extremely high values of the force constants for the two angle bending terms involving the dummy atom would be necessary to prevent flapping.

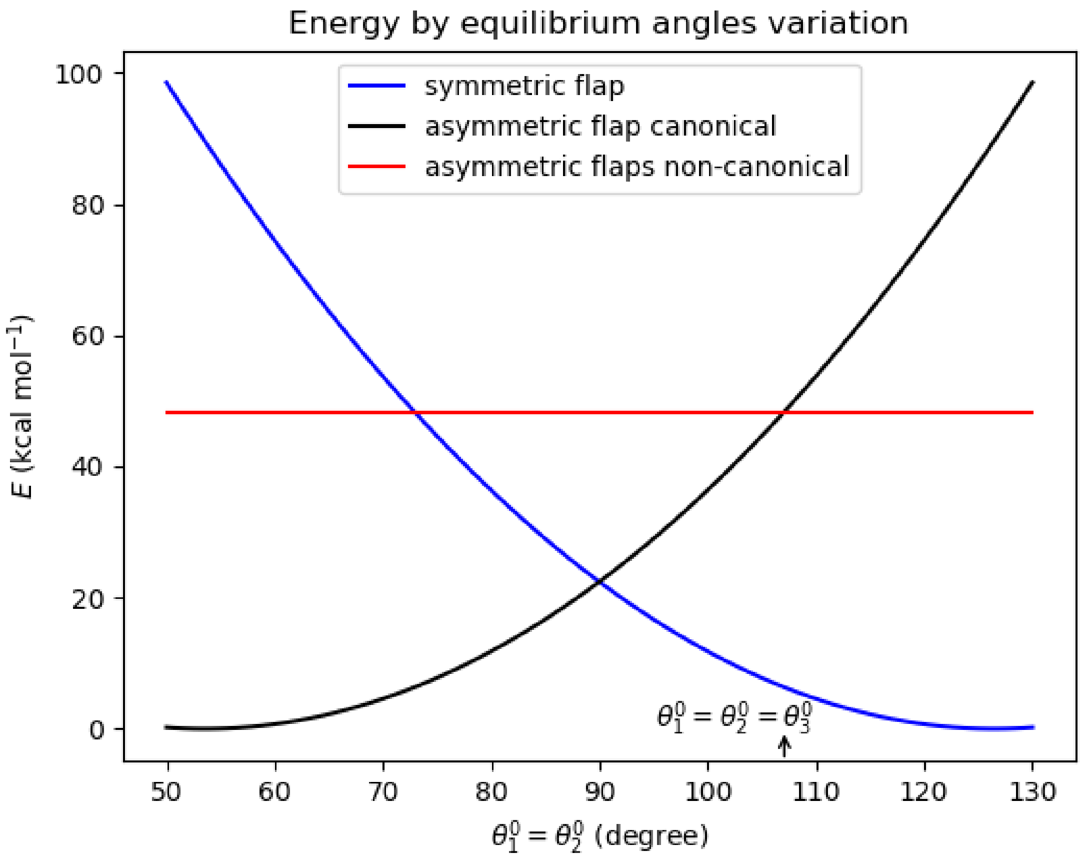

Figure 4: Dependence of the energy barriers for a pyramidal flap on the dummy angle equilibrium values. $\theta_3^0 = 107.1$ deg and $k_1 = k_2 = k_3 = 41.50$ kcal mol^{-1} rad^{-2}. There is a sweet spot at $\theta_1^0 = \theta_2^0 = 90$ deg. Index 3 is tied to the physical angle.

Since we know from considerations concerning the separability of the partition function (see Sect 2.1 above and Sect 2.1.2 of the main manuscript) that the equilibrium angles for the two terms involving the dummy atom should be set to 90°, we explore next whether and how the findings from Fig. 3 change as a function of the equilibrium angles θ_1^0 and θ_2^0 (the

angle degrees of freedom involving the dummy atom). The resulting plot is shown in Fig. 4. All three force constants are set to a fixed value, the equilibrium value of the physical angle θ_3^0 is set to 107.1°, whereas the equilibrium angles of the dummy angles are varied. We now can distinguish three cases. The two non-canonical symmetric flaps (Figs. 2(d) and (e)) lead to a high energy, flat curve (in red). If one again ties index 3 in Eq. 25 to the physical angle, then these two pathways correspond to exchanging index 3 in Eq. 25 with index 1 or 2, respectively. While Eq. 25 is invariant to changing the indices of the force constants k_i, this is not the case for the equilibrium angles because of the $\theta_3^0 - \theta_2^0 - \theta_1^0$ term (in Fig. 3, we had $\theta_1^0 = \theta_2^0 = \theta_3^0$, but now we are varying θ_1^0 and θ_2^0). For the two asymmetric non-canonical cases, by index permutation, the term reads either $\theta_1^0 - \theta_2^0 - \theta_3^0$ or $\theta_2^0 - \theta_3^0 - \theta_1^0$. Thus, any variation of θ_2^0 and θ_1^0 cancels, resulting in the flat red function shown in Fig. 4.

The much more interesting feature of Fig. 4, however, is the mirror symmetry, centered around 90°, between the symmetric (black curve) and the asymmetric canonical energy barriers (blue curve). This cross-over point at 90° can be understood considering Figs. 2(b) and (c). If $\theta_1 = \theta_2 = 90°$, the physical angle, i.e. θ_3 in Fig. 2(b) and θ_3' in Fig. 2(c), is at 180°. Then, the two cases are essentially equivalent. Lowering the physical angle (θ_3 or θ_3') forces θ_1 and θ_2 out of their equilibrium position at 90°. Only the direction is different: in the symmetric case, θ_1 and θ_2 are forced to values $> 90°$, whereas in the asymmetric case, θ_1 and θ_2 are forced to values $< 90°$. The magnitude of these elongations, $|\theta_1 - \pi/2|$ and $|\theta_2 - \pi/2|$, by symmetry arguments, is the same in both cases. The energy function is quadratic and depends only on the magnitude of the displacement, which explains the mirror symmetry of the energy barriers. For the force constant chosen in Fig. 4 and with dummy equilibrium angles of 107.1°, the energy barrier of the symmetric flapping pathway is only 6.3 kcal/mol, too low to prevent flapping. As one can see in Fig. 4, setting the dummy equilibrium angles to 90°, increases the barrier to > 20 kcal/mol. Using slightly higher than usual force constants ($k_1 = k_2 = 100$ kcal mol^{-1}rad^{-2}) raises the barrier height > 35 kcal/mol, sufficiently high so that a flap will not be encountered during finite simulation lengths. Additionally, proceeding

in this manner blocks both the symmetric and asymmetric flapping pathways equally.

3 Error analysis in thermodynamic integration

Free energy differences were calculated using Thermodynamic Integration (TI),[10,11] as well as Bennett's acceptance ratio (BAR).[12] The results obtained with the latter were computed as a control; for additional details see Sect. 5 below. Each of our 5×21 simulations ($N = 5$ repetitions, $K = 21$ λ-states) per system and transformation are statistically independent. Using BAR, each of the individual $\Delta A_{\lambda_i \to \lambda_{i+1}}$, $(i = 1, \dots K-1)$ was, therefore, obtained from five independent simulations; similarly, in TI we have five values for each of the K derivatives $\langle \frac{\partial U}{\partial \lambda} \rangle_{\lambda_i}$ $(i = 1, \dots K)$. In the case of BAR, Gaussian error propagation based on the standard deviation of the individual $\Delta A_{\lambda_i \to \lambda_{i+1}}$ is straightforward; in the following, we outline how we estimated the overall statistical error using TI.

In TI, the integral over λ has to be approximated by numerical quadrature. Numerical integration algorithms, applied to TI, can formally always be written as

$$\Delta G_r = \int_0^1 d\lambda \left\langle \frac{\partial U}{\partial \lambda} \right\rangle_{\lambda, r} \approx \sum_{i=1}^{K} W_{\lambda_i} \left\langle \frac{\partial U}{\partial \lambda} \right\rangle_{\lambda_i, r} \tag{26}$$

where $\left\langle \frac{\partial U}{\partial \lambda} \right\rangle_{\lambda_i, r}$ is the potential energy derivative of the ith λ-state averaged over the rth of our $N = 5$ independent simulations and $K = 21$ denotes the number of simulated λ-states. The W_{λ_i} are constants depending on the quadrature scheme. If they are known (as, e.g., when using the trapezoidal rule, Simpson's rule, Gauss-Legendre or Clenchaw-Curtis integration), error propagation based on the standard deviation of the individual $\left\langle \frac{\partial U}{\partial \lambda} \right\rangle_{\lambda_i}$ is straightforward.

In this work, however, we fitted the integrand using natural cubic splines, approximating it in a piece-wise continuous manner by cubic polynomials, which were then integrated analytically.[13,14] Denoting this spline-fit-then-integrate approach as splint[], one can show that[13]

$$\Delta G_r = \int_0^1 d\lambda \left\langle \frac{\partial U}{\partial \lambda} \right\rangle_{\lambda, r} \approx \text{splint}\left[\left\langle \frac{\partial U}{\partial \lambda} \right\rangle_{\lambda_1, r}, \dots, \left\langle \frac{\partial U}{\partial \lambda} \right\rangle_{\lambda_K, r} \right] = \sum_{i=1}^{K} W_{\lambda_i} \left\langle \frac{\partial U}{\partial \lambda} \right\rangle_{\lambda_i, r}; \tag{27}$$

i.e., splint[] can be regarded as any other numerical quadrature method. However, obtaining the coefficients W_{λ_i} is not as obvious as in standard numerical quadrature methods. Because of the linearity of the weighted sum in Eq. 27, we find for the free energy difference ΔG, i.e., the average over the $N = 5$ independent results ΔG_r:

$$\begin{aligned}
\Delta G &= \frac{1}{N}\sum_{r=1}^{N} \Delta G_r = \frac{1}{N}\sum_{r=1}^{N} \text{splint}\left[\left\langle\frac{\partial U}{\partial\lambda}\right\rangle_{\lambda_1,r}, \ldots, \left\langle\frac{\partial U}{\partial\lambda}\right\rangle_{\lambda_K,r}\right] \\
&= \text{splint}\left[\overline{\left\langle\frac{\partial U}{\partial\lambda}\right\rangle_{\lambda_1,r}}, \ldots, \overline{\left\langle\frac{\partial U}{\partial\lambda}\right\rangle_{\lambda_K,r}}\right] \\
\overline{\left\langle\frac{\partial U}{\partial\lambda}\right\rangle_{\lambda_i}} &= \frac{1}{N}\sum_{r=1}^{N} \left\langle\frac{\partial U}{\partial\lambda}\right\rangle_{\lambda_j,r}
\end{aligned} \tag{28}$$

The corresponding standard deviation σ is

$$\begin{aligned}
\sigma &= \sqrt{\sum_{i=1}^{K} W_{\lambda_i}^2 \sigma_i^2} \\
\sigma_i &= \sqrt{\frac{1}{N-1}\sum_{r=1}^{N}\left(\left\langle\frac{\partial U}{\partial\lambda}\right\rangle_{\lambda_i,r} - \overline{\left\langle\frac{\partial U}{\partial\lambda}\right\rangle_{\lambda_i}}\right)^2}
\end{aligned} \tag{29}$$

The coefficients W_{λ_i} can be extracted by means of custom code from spline routine used; this is, e.g., done in the widely-used software package Alchemical Analysis.[15] We took a different, yet mathematically equivalent approach, which requires neither knowledge of implementation dependent representations of the coefficients W_{λ_i}, nor of the computational details of the spline routine used. Specifically, we used the Python package SciPy (www.scipy.org), which wraps the FITPACK[16] library, but any numerical package capable of fitting and integrating natural cubic splines could have been chosen.

In order to compute the standard deviation σ (Eq. 29) without explicit knowledge of the

W_{λ_i}, we define

$$
\begin{aligned}
s &= \sqrt{\sum_{i=1}^{K} (s_{\lambda_i})^2} \\
s_{\lambda_i} &= \sqrt{\frac{1}{N-1} \sum_{r=1}^{N} \left[\mathrm{splint}_{\lambda_i} \left(\left\langle \frac{\partial U}{\partial \lambda} \right\rangle_{\lambda_i, r} \right) - \mathrm{splint}_{\lambda_i} \left(\overline{\left\langle \frac{\partial U}{\partial \lambda} \right\rangle}_{\lambda_i} \right) \right]^2} \\
\mathrm{splint}_{\lambda_i}(x) &= \mathrm{splint} \left[\overline{\left\langle \frac{\partial U}{\partial \lambda} \right\rangle}_{\lambda_1}, \ldots, \overline{\left\langle \frac{\partial U}{\partial \lambda} \right\rangle}_{\lambda_{i-1}}, x, \ \overline{\left\langle \frac{\partial U}{\partial \lambda} \right\rangle}_{\lambda_{i+1}}, \ldots \overline{\left\langle \frac{\partial U}{\partial \lambda} \right\rangle}_{\lambda_K} \right]
\end{aligned} \tag{30}
$$

Because Eq. 27 is a linear sum of the values of the integrand $\left\langle \frac{\partial U}{\partial \lambda} \right\rangle_{\lambda_i, r}$ at the λ-states, most of the terms $\overline{\left\langle \frac{\partial U}{\partial \lambda} \right\rangle}_{\lambda_i}$ cancel from Eq. 30 and one obtains

$$
\mathrm{splint}_{\lambda_i} \left(\left\langle \frac{\partial U}{\partial \lambda} \right\rangle_{\lambda_j, r} \right) - \mathrm{splint}_{\lambda_i} \left(\overline{\left\langle \frac{\partial U}{\partial \lambda} \right\rangle}_{\lambda_i} \right) = W_{\lambda_i} \left(\left\langle \frac{\partial U}{\partial \lambda} \right\rangle_{\lambda_i, r} - \overline{\left\langle \frac{\partial U}{\partial \lambda} \right\rangle}_{\lambda_i} \right) \tag{31}
$$

From Eq. 31 one sees that s in Eq. 30 and σ in Eq. 29 are, in fact, identical. Pseudo code for the approach outlined in Eqs. 30 and 31 is given in Sect. 4.

Our approach is very similar to the methodology available in the Alchemical Analysis package,[15] except for the following two differences. First, we use five independent simulations to compute the mean and standard deviation of $\left\langle \frac{\partial U}{\partial \lambda} \right\rangle$ instead of instantaneous $\frac{\partial U}{\partial \lambda}$ values from a single trajectory, corrected for statistical independence. Our approach outlined above can be cast into their formalism, if one considers $\left\langle \frac{\partial U}{\partial \lambda} \right\rangle_{\lambda_i, r}$ as the instantaneous value of $\frac{\partial U}{\partial \lambda}$ at frame number r (of state λ_i). Second, we report standard deviations instead of standard errors of the mean. To convert to the latter, our standard deviations would have to be scaled by $1/\sqrt{5} \approx 0.447$.

4 Pseudo code for statistical error estimation in the spline based thermodynamic integration approach used

Algorithm 1 Spline integration error propagation

```
### Calculate average ∂U/∂λ over replicas
for λ ← 1, num_lambdas do
    dudl_aver[λ] ← mean_r(dudl[λ][r])
end for

### Yield the average
result_average ← splineintegrate(dudl_aver)

### Calculate the s_λi
for λ ← 1, num_lambdas do
    dudl_tmp ← dudl_aver #copy the whole array
    for r ← 1, num_replicas do
        dudl_tmp[λ] ← dudl[λ][r]
        spl_int[r] ← splineintegrate(dudl_tmp)
    end for
    stddev_lam[λ]←stddev_r(spl_int[r])
end for

### Yield the total standard deviation via error propagation
result_stddev ← 0
for λ ← 1, num_lambdas do
    result_stddev ← result_stddev + (stddev_lam[λ])^2
end for
result_stddev ← √(result_stddev)
```

The above pseudo-code complements the description of Sect. 3. The individual, statistically independent $\left\langle \frac{\partial U}{\partial \lambda} \right\rangle_{\lambda_i,r}$ values ($r = 1, \ldots N$, $i = 1, \ldots K$, with $K = 21$, $N = 5$) are stored in the two-dimensional array dudl. The function splineintegrate() in the above code corresponds to the splint[] function of Sect. 3.

First, the array dudl_aver is filled with the averages over replicas $\overline{\left\langle \frac{\partial U}{\partial \lambda} \right\rangle}_{\lambda_i}$ for each state λ_i from the data $\left\langle \frac{\partial U}{\partial \lambda} \right\rangle_{\lambda_i,r}$ stored in dudl. The estimate for ΔG, i.e. result_average, is calculated

as the spline integral over dudl_aver; cf. Eq. 29.

This is followed by the estimation of the standard deviation s_{λ_i} for each λ-state; cf. Eq. 31. For each λ_i, the respective entry in dudl_aver[λ] is replaced by each of the individual values $\left\langle \frac{\partial U}{\partial \lambda} \right\rangle_{\lambda_i,r}$ from dudl[λ][r]. The resulting array, labeled dudl_tmp, is evaluated with splineintegrate() and the result stored in spl_int[r]. The latter is used to compute the standard deviation s_{λ_i} for each of the λ_i.

The overall error estimate is obtained by Gaussian error propagation. For further details, the reader is referred to the input scripts and source code available at Zenodo (https://zenodo.org/record/4381708).[17]

5 Detailed specifications for each transformation studied

Full technical details and additional results are summarized on one page per transformation for each of the 12 relative solvation free energy differences reported in the main manuscript.

For each alchemical pair, there is a figure showing the atom labels used. The accompanying table lists on the left hand side gas phase and solvation free energy differences obtained along the absolute and relative paths. If a relative free energy difference was computed with more than one approach, then individual results are given for each of them.

The right hand side of each table for the twelve alchemical transformations lists force field terms involving dummy atoms, which were either deleted and/or modified. Illustrative examples of such lists were given in Table 3 of the main manuscript. If more than one approach was used to carry out the alchemical transformation, this is indicated by footnotes. Urey-Bradley terms involving dummy atoms which would lead to a constraint were always deleted; this is not explicitly noted.

We report free energy differences obtained with thermodynamic integration (TI),[10] as well as Bennett's acceptance ratio (BAR) method[12] (see below). Tables 4 and 5 of the main manuscript were compiled from the TI results reported here. All free energy differences were also calculated using BAR based on the same underlying MD simulations used for TI. Coordinates were saved to disk every 50 steps (cf. Methods of main manuscript); each trajectory saved at λ_i was used to compute energies at λ_{i-1}, λ_i and λ_{i+1}. Thus, the overall free energy difference of a transformation was calculated as the sum of 20 individual free energy differences; except for PRP2DIM-3, where we employed 39 λ-states and hence the total free energy difference is the sum of 38 contributions.

As described in the main manuscript, each simulation was repeated five times (by starting from different random initial velocities); hence, for each free energy difference between λ_i and λ_{i+1} we computed five values $\Delta A^j_{\lambda_i \to \lambda_{i+1}}$ ($j = 1, \ldots 5$). Rather than just averaging

over these (i.e., $\langle \Delta A \rangle_{\lambda_i \to \lambda_{i+1}} = 1/5 \sum_{j=1}^{5} \Delta A^{j}_{\lambda_i \to \lambda_{i+1}}$), we combined the raw data from the respective simulations (forward and backward energy differences), and computed our best expectation value for the free energy difference $\Delta A^{E}_{\lambda_i \to \lambda_{i+1}}$ from these combined data. The statistical uncertainty for this step was estimated as the standard deviation of the five individual $\Delta A^{j}_{\lambda_i \to \lambda_{i+1}}$ values. Thus, using BAR, the total free energy difference was estimated as $\Delta A^{E}_{0 \to 1} = \sum_{k=1}^{20} \Delta A^{E}_{\lambda_i \to \lambda_{i+1}}$. The corresponding error estimate was obtained by Gaussian error propagation from the variances $\sigma^2(\Delta A_{\lambda_i \to \lambda_{i+1}})$ of the individual steps.

Tools to (re)create all calculations reported in this work have been uploaded to Zenodo.[17]

5.1 Terminal junctions

5.1.1 Hexane–Propane

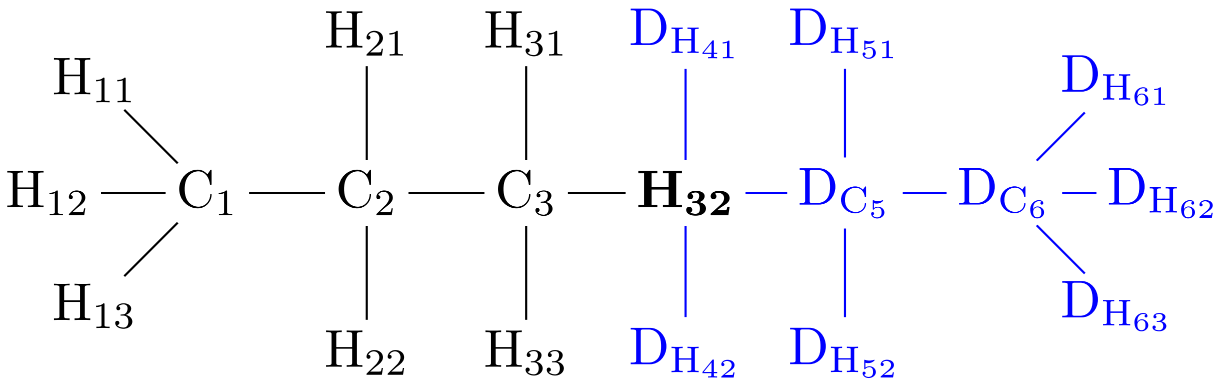

Figure 5: Hexane→Propane transformation, Propane endpoint

Relative	$\Delta\Delta G_{\mathrm{solv}}$	ΔG_{aq}	ΔG_{gas}
HEX2PRP-1			
TI	-0.47 ± 0.04	-4.02 ± 0.03	-3.55 ± 0.02
BAR	-0.47 ± 0.04	-4.01 ± 0.05	-3.54 ± 0.01
HEX2PRP-2[a]			
TI	-0.50 ± 0.05	-3.44 ± 0.04	-2.94 ± 0.03
BAR	-0.51 ± 0.08	-3.44 ± 0.03	-2.93 ± 0.07

Absolute	$\Delta G^{\mathrm{abs}}_{\mathrm{solv}}$	$\Delta G^{\mathrm{abs}}_{\mathrm{aq}}$	$\Delta G^{\mathrm{abs}}_{\mathrm{gas}}$
Hexane			
TI	2.82 ± 0.06	-7.98 ± 0.05	-5.16 ± 0.02
BAR	2.83 ± 0.07	-7.99 ± 0.07	-5.16 ± 0.05
Propane			
TI	2.33 ± 0.03	-5.31 ± 0.03	-2.98 ± 0.00
BAR	2.34 ± 0.04	-5.32 ± 0.04	-2.98 ± 0.00
Hexane → Propane		$\Delta\Delta G^{\mathrm{abs}}_{\mathrm{solv}}$	
TI		-0.49 ± 0.06	
BAR		-0.49 ± 0.03	

Deleted:
D_{C_5}-H_{32}-C_3-H_{31}
D_{C_5}-H_{32}-C_3-H_{32}
$D_{H_{41}}$-H_{32}-C_3-H_{31}
$D_{H_{41}}$-H_{32}-C_3-H_{32}
$D_{H_{42}}$-H_{32}-C_3-H_{31}
$D_{H_{43}}$-H_{32}-C_3-H_{32}

Modified:
None

[a] naive approach, see introduction.

5.1.2 Toluene–Methane

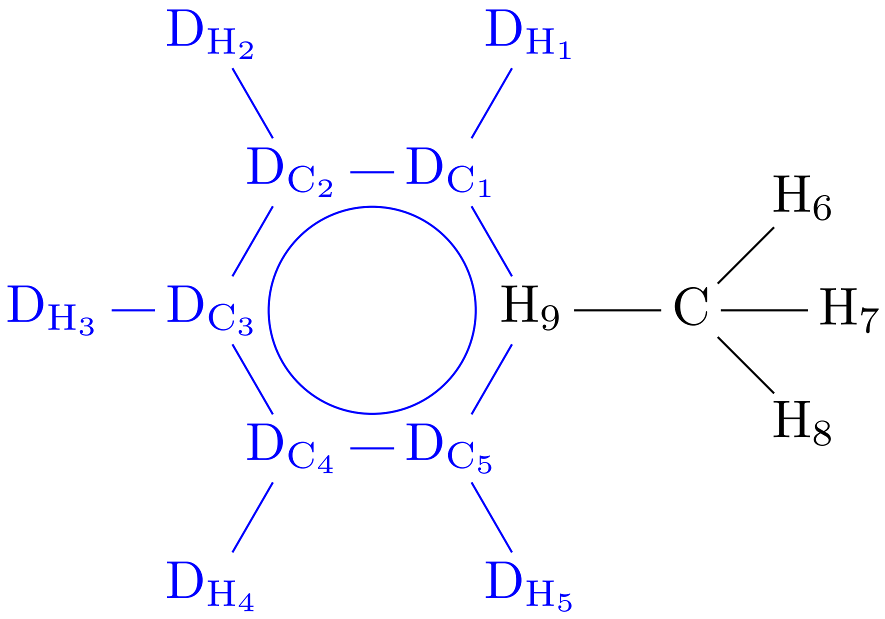

Figure 6: Toluene→Methane transformation, Methane endpoint

Relative	$\Delta\Delta G_{\text{solv}}$	ΔG_{aq}	ΔG_{gas}
TOL2MET			
TI	2.46 ± 0.02	-3.43 ± 0.02	-5.89 ± 0.00
BAR	2.44 ± 0.02	-3.43 ± 0.02	-5.87 ± 0.03
Absolute	$\Delta G^{\text{abs}}_{\text{solv}}$	$\Delta G^{\text{abs}}_{\text{aq}}$	$\Delta G^{\text{abs}}_{\text{gas}}$
Toluene[a]			
TI	-0.10 ± 0.05	-5.97 ± 0.05	-6.07 ± 0.00
BAR	-0.06 ± 0.04	-6.00 ± 0.04	-6.06 ± 0.00
Methane[b]			
TI	2.40 ± 0.02	-2.40 ± 0.02	0.00 ± 0.00
BAR	2.39 ± 0.01	-2.39 ± 0.01	0.00 ± 0.00
Toluene[a] → Methane[b]		$\Delta\Delta G^{\text{abs}}_{\text{solv}}$	
TI		2.50 ± 0.05	
BAR		2.46 ± 0.04	

Deleted:
D_{C_1}-H_9-C-H_7
D_{C_5}-H_9-C-H_7
D_{C_1}-H_9-C-H_8
D_{C_5}-H_9-C-H_8

Modified:
None

[a] with 567 water molecules;
[b] with 567 water molecules.

5.2 Dual Junctions

5.2.1 Ethane–Methanol

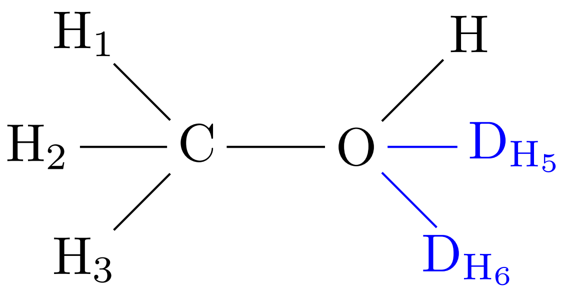

Figure 7: Ethane→Methanol transformation, Methanol endpoint

Relative	$\Delta\Delta G_{solv}$	ΔG_{aq}	ΔG_{gas}
ETH2MEOH			
TI	-6.93 ± 0.03	0.24 ± 0.03	7.17 ± 0.00
BAR	-6.95 ± 0.04	0.23 ± 0.03	7.18 ± 0.04
Absolute	ΔG^{abs}_{solv}	ΔG^{abs}_{aq}	ΔG^{abs}_{gas}
Ethane			
TI	2.25 ± 0.02	-11.11 ± 0.02	-8.86 ± 0.00
BAR	2.25 ± 0.02	-11.11 ± 0.02	-8.86 ± 0.00
Methanol			
TI	-4.69 ± 0.02	-10.59 ± 0.02	-15.28 ± 0.00
BAR	-4.70 ± 0.02	-10.59 ± 0.02	-15.29 ± 0.00
Ethane → Methanol		$\Delta\Delta G^{abs}_{solv}$	
TI		-6.94 ± 0.02	
BAR		-6.95 ± 0.01	

Deleted:
D_{H_5}-O-D_{H_6}
D_{H_5}-O-C-H_1
D_{H_5}-O-C-H_2
D_{H_5}-O-C-H_3
D_{H_6}-O-C-H_1
D_{H_6}-O-C-H_2
D_{H_6}-O-C-H_3

Modified[a]:
D_{H_5}-O-C
D_{H_5}-O-H
D_{H_6}-O-C
D_{H_6}-O-H

[a] all to 90° equilibrium angle and 100 kcal/rad^2 force constant.

5.2.2 Methane–Water

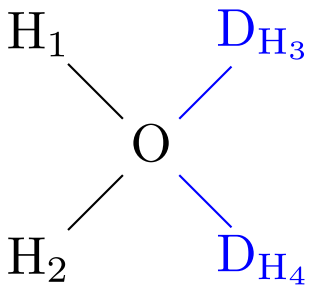

Figure 8: Methane→Water transformation, Water endpoint

Relative	$\Delta\Delta G_{solv}$	ΔG_{aq}	ΔG_{gas}
MET2WAT			
TI	-9.18 ± 0.04	-7.81 ± 0.04	1.36 ± 0.01
BAR	-9.19 ± 0.03	-7.83 ± 0.04	1.36 ± 0.01
MET2WAT-qfs[a]			
TI	-9.24 ± 0.03	-7.93 ± 0.03	1.31 ± 0.00
BAR	-9.29 ± 0.02	-7.94 ± 0.03	1.36 ± 0.01
Absolute	ΔG^{abs}_{solv}	ΔG^{abs}_{aq}	ΔG^{abs}_{gas}
Methane[b]			
TI	2.38 ± 0.02	-2.38 ± 0.02	0.00 ± 0.00
BAR	2.38 ± 0.01	-2.38 ± 0.01	0.00 ± 0.00
Water			
TI	-6.89 ± 0.01	6.89 ± 0.01	0.00 ± 0.00
BAR	-6.89 ± 0.01	6.89 ± 0.01	0.00 ± 0.00
Water[a]			
TI	-6.88 ± 0.01	6.88 ± 0.01	0.00 ± 0.00
BAR	-6.89 ± 0.01	6.89 ± 0.01	0.00 ± 0.00
Methane[b] → Water[a]		$\Delta\Delta G^{abs}_{solv}$	
TI		-9.27 ± 0.02	
BAR		-9.27 ± 0.02	

Deleted:
D_{H_3}-O-D_{H_4}

Modified[c]:
D_{H_3}-O-H_1
D_{H_3}-O-H_2
D_{H_4}-O-H_1
D_{H_4}-O-H_2

[a] instead of the 1 fs integration step used in all other simulations, due to the small physical molecule size, a 0.25 fs integrator step was used here;
[b] with 567 water molecules, see methane-ammonia);
[c] all to 90° equilibrium angle and 100 kcal/rad^2 force constant.

5.2.3 Toluene–Pyridine

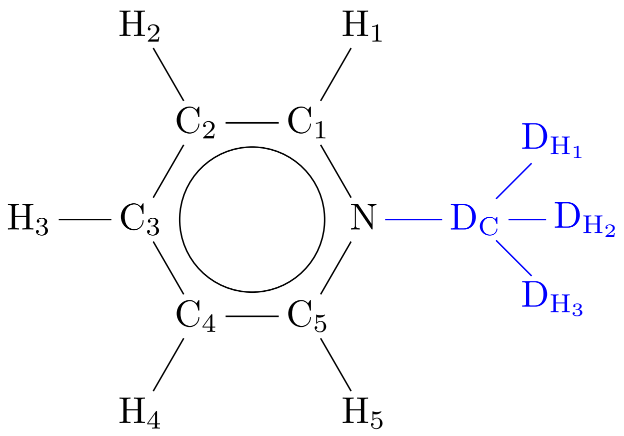

Figure 9: Toluene→Pyridine transformation, Pyridine endpoint

Relative	$\Delta\Delta G_{\mathrm{solv}}$	ΔG_{aq}	ΔG_{gas}
TOL2PYR-1[a]			
TI	-4.69 ± 0.02	3.97 ± 0.02	8.66 ± 0.01
BAR	-4.69 ± 0.02	3.97 ± 0.02	8.66 ± 0.01
TOL2PYR-2[b]			
TI	-4.67 ± 0.02	4.14 ± 0.02	8.81 ± 0.00
BAR	-4.67 ± 0.01	4.13 ± 0.02	8.81 ± 0.01
Absolute	$\Delta G^{\mathrm{abs}}_{\mathrm{solv}}$	$\Delta G^{\mathrm{abs}}_{\mathrm{aq}}$	$\Delta G^{\mathrm{abs}}_{\mathrm{gas}}$
Toluene[c]			
TI	-0.08 ± 0.03	-5.98 ± 0.03	-6.07 ± 0.00
BAR	-0.05 ± 0.02	-6.02 ± 0.03	-6.06 ± 0.00
Pyridine			
TI	-4.75 ± 0.03	-10.00 ± 0.03	-14.75 ± 0.00
BAR	-4.73 ± 0.03	-10.03 ± 0.04	-14.75 ± 0.00
Toluene[c] → Pyridine		$\Delta\Delta G^{\mathrm{abs}}_{\mathrm{solv}}$	
TI		-4.67 ± 0.04	
BAR		-4.68 ± 0.04	

Deleted:
D_C-N-C_5 [b]
D_C-N-C_1-C_2 [a]
D_C-N-C_1-H_1
D_C-N-C_5-H_5
D_C-N-C_5-C_4
D_{H_1}-D_C-N-C_5
D_{H_2}-D_C-N-C_5
D_{H_3}-D_C-N-C_5

Modified:
D_C-N-C_1-C_2 [b,e]
D_C-N-C_1 [a,d]
D_C-N-C_5 [a,d]

[a] bond and two angles approach;
[b] bond, angle, dihedral approach;
[c] with 562 water molecules;
[d] both to 90° equilibrium angle and 100 kcal/rad^2 force constant;
[e] to 0° equilibrium angle without periodicity and 100 kcal force constant.

5.2.4 Hexane–Propanol

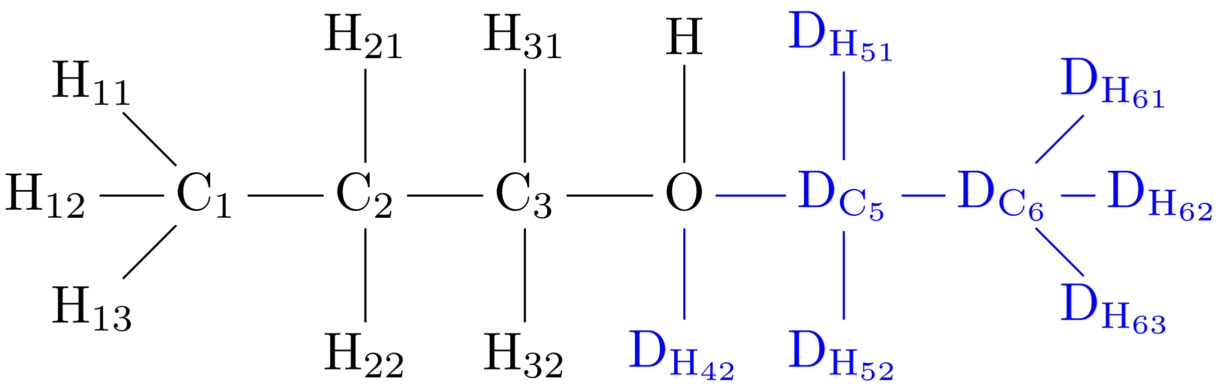

Figure 10: Hexane→Propanol transformation, Propanol endpoint, canonical transformation

Relative	$\Delta\Delta G_{\text{solv}}$	ΔG_{aq}	ΔG_{gas}
HEX2POL-1			
TI	-7.09 ± 0.04	-4.59 ± 0.03	2.50 ± 0.02
BAR	-7.09 ± 0.08	-4.58 ± 0.05	2.51 ± 0.04
HEX2POL-2[a]			
TI	-7.09 ± 0.05	-3.95 ± 0.04	3.15 ± 0.03
BAR	-7.10 ± 0.06	-3.95 ± 0.05	3.15 ± 0.03

Absolute	$\Delta G^{\text{abs}}_{\text{solv}}$	$\Delta G^{\text{abs}}_{\text{aq}}$	$\Delta G^{\text{abs}}_{\text{gas}}$
Hexane			
TI	2.82 ± 0.06	-7.98 ± 0.05	-5.16 ± 0.02
BAR	2.83 ± 0.07	-7.99 ± 0.07	-5.16 ± 0.05
Propanol			
TI	-4.28 ± 0.05	-3.48 ± 0.04	-7.77 ± 0.02
BAR	-4.28 ± 0.02	-3.48 ± 0.01	-7.77 ± 0.02
Hexane → Propanol		$\Delta\Delta G^{\text{abs}}_{\text{solv}}$	
TI		-7.11 ± 0.07	
BAR		-7.11 ± 0.08	

Deleted:

D_{C_5}-O-$D_{H_{42}}$
D_{C_5}-O-C_3-C_2
D_{C_5}-O-C_3-H_{31}
D_{C_5}-O-C_3-H_{32}
D_{C_6}-D_{C_5}-O-H
$D_{H_{51}}$-D_{C_5}-O-H
$D_{H_{52}}$-D_{C_5}-O-H
$D_{H_{42}}$-O-C_3-C_2
$D_{H_{42}}$-O-C_3-H_{31}
$D_{H_{42}}$-O-C_3-H_{32}
$D_{H_{42}}$-O-D_{C_5}-D_{C_6}
$D_{H_{42}}$-O-D_{C_5}-$D_{H_{51}}$
$D_{H_{42}}$-O-D_{C_5}-$D_{H_{52}}$

Modified:

D_{C_5}-O-C_3[b]
D_{C_5}-O-H[b]
$D_{H_{42}}$-O-C_3[b]
$D_{H_{42}}$-O-H[b]

[a] naive approach, see introduction;
[b] all to 90° equilibrium angle and 100 kcal/rad^2 force constant.

5.2.5 Propane–Dimethylether

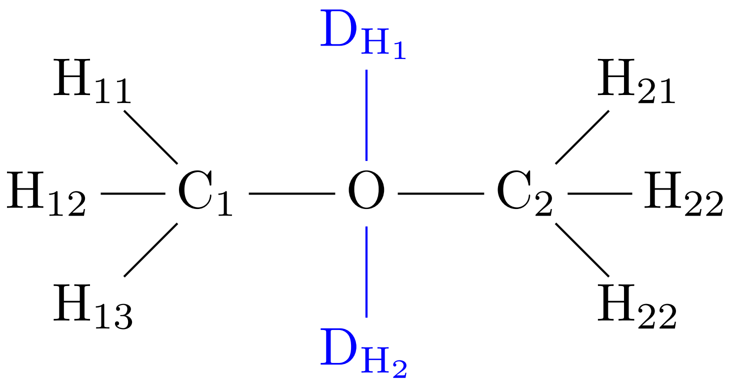

Figure 11: Propane→Dimethylether transformation, Dimethylether endpoint

Relative	$\Delta\Delta G_{\text{solv}}$	ΔG_{aq}	ΔG_{gas}
PRP2DIM-1[a]			
TI	-3.33 ± 0.02	-4.00 ± 0.02	-0.67 ± 0.00
BAR	-3.33 ± 0.02	-3.98 ± 0.03	-0.66 ± 0.02
PRP2DIM-2[b]			
TI	-3.36 ± 0.02	-7.69 ± 0.02	-4.32 ± 0.01
BAR	-3.36 ± 0.02	-6.92 ± 0.03	-3.56 ± 0.01
PRP2DIM-3[b,c]			
TI	-3.34 ± 0.02	-6.91 ± 0.02	-3.58 ± 0.01
BAR	-3.34 ± 0.02	-6.91 ± 0.02	-3.57 ± 0.01
Absolute	$\Delta G^{\text{abs}}_{\text{solv}}$	$\Delta G^{\text{abs}}_{\text{aq}}$	$\Delta G^{\text{abs}}_{\text{gas}}$
Propane			
TI	2.33 ± 0.03	-5.31 ± 0.03	-2.98 ± 0.00
BAR	2.34 ± 0.04	-5.32 ± 0.04	-2.98 ± 0.00
Dimethylether			
TI	-1.02 ± 0.02	-0.93 ± 0.02	-1.95 ± 0.00
BAR	-1.01 ± 0.03	-0.93 ± 0.03	-1.95 ± 0.00
Propane → Dimethylether		$\Delta\Delta G^{\text{abs}}_{\text{solv}}$	
TI		-3.36 ± 0.03	
BAR		-3.35 ± 0.04	

Deleted:
D_{H_1}-O-D_{H_2}[a]
D_{H_1}-O-C_2[b]
D_{H_2}-O-C_2[b]
D_{H_1}-O-C_1-H_{11}[a]
D_{H_1}-O-C_1-H_{12}
D_{H_1}-O-C_1-H_{13}
D_{H_1}-O-C_2-H_{21}
D_{H_1}-O-C_2-H_{22}
D_{H_1}-O-C_2-H_{23}
D_{H_2}-O-C_1-H_{11}[a]
D_{H_2}-O-C_1-H_{12}
D_{H_2}-O-C_1-H_{13}
D_{H_2}-O-C_2-H_{21}
D_{H_2}-O-C_2-H_{22}
D_{H_2}-O-C_2-H_{23}

Modified:
D_{H_1}-O-C_1[a,d]
D_{H_2}-O-C_1[a,d]
D_{H_1}-O-C_2[a,d]
D_{H_2}-O-C_2[a,d]

[a] bond and two angles approach;
[b] bond, angle, dihedral approach;
[c] fine-graining of the λ-states at the Dimethylether end state, see main text;
[d] 90° equilibrium angle and 100 kcal/rad^2 force constant.

5.3 Triple Junctions

5.3.1 Acetone–2-Propenol

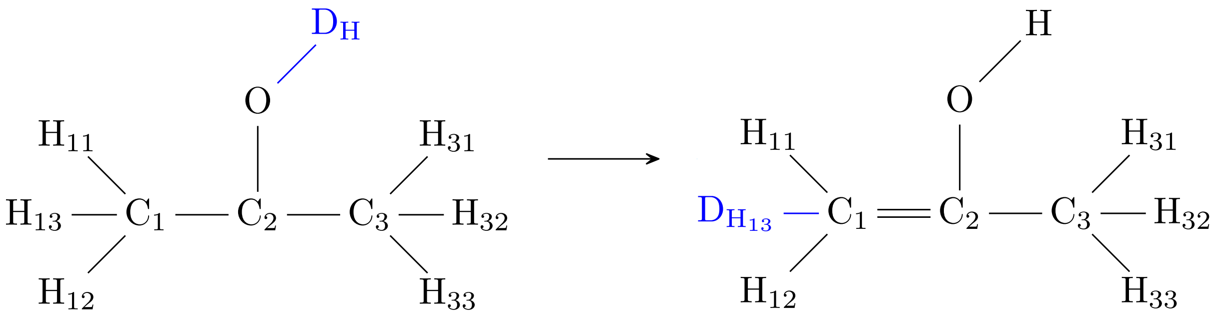

Figure 12: Acetone→2-Propenol transformation

Relative	$\Delta\Delta G_{\text{solv}}$	ΔG_{aq}	ΔG_{gas}
ACE2PEOL-1			
TI	1.03 ± 0.02	-21.71 ± 0.02	-22.74 ± 0.01
BAR	1.03 ± 0.04	-21.71 ± 0.03	-22.73 ± 0.01

Absolute	$\Delta G^{\text{abs}}_{\text{solv}}$	$\Delta G^{\text{abs}}_{\text{aq}}$	$\Delta G^{\text{abs}}_{\text{gas}}$
Acetone			
TI	-4.28 ± 0.02	40.90 ± 0.02	36.62 ± 0.00
BAR	-4.27 ± 0.02	40.89 ± 0.02	36.61 ± 0.00
2-Propenol			
TI	-3.23 ± 0.02	63.66 ± 0.02	60.43 ± 0.00
BAR	-3.23 ± 0.02	63.66 ± 0.02	60.43 ± 0.00
Acetone → 2-Propenol		$\Delta\Delta G^{\text{abs}}_{\text{solv}}$	
TI		1.05 ± 0.03	
BAR		1.04 ± 0.02	

Triple junction[b]:
planar

Deleted:
D_H-O-C_2-C_1 [a]
$D_{H_{13}}$-C_1-H_{12} [b]
$D_{H_{13}}$-C_1-C_2-C_3 [b]
$D_{H_{13}}$-C_1-C_2-O [b]

Modified[b,c]:
$D_{H_{13}}$-C_1-H_{11} [b,c]
$D_{H_{13}}$-C_1-C_2 [b,c]

[a] Acetone endpoint;
[b] 2-Propenol endpoint;
[c] both to 90° equilibrium angle and 100.0 kcal/rad^2 force constant.

5.3.2 Phenol–Cyclohexadienone

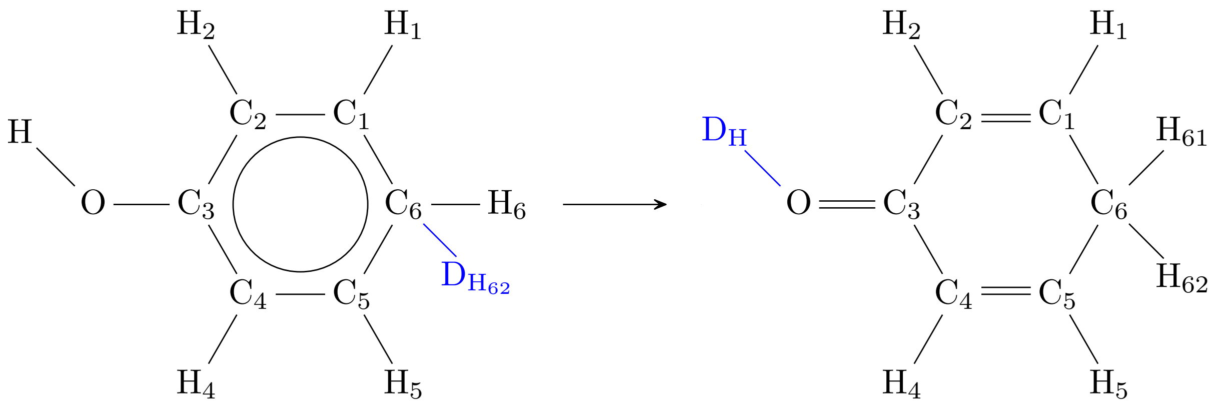

Figure 13: Phenol→Cyclohexadienone transformation

Relative	$\Delta\Delta G_{\text{solv}}$	ΔG_{aq}	ΔG_{gas}
PHE2CYC			
TI	-0.01 ± 0.02	8.41 ± 0.02	8.42 ± 0.00
BAR	-0.02 ± 0.04	8.41 ± 0.03	8.43 ± 0.02

Absolute	$\Delta G^{\text{abs}}_{\text{solv}}$	$\Delta G^{\text{abs}}_{\text{aq}}$	$\Delta G^{\text{abs}}_{\text{gas}}$
Phenol			
TI	-4.78 ± 0.04	4.39 ± 0.04	-0.39 ± 0.00
BAR	-4.74 ± 0.04	4.34 ± 0.04	-0.39 ± 0.00
Cyclohexadienone			
TI	-4.77 ± 0.04	-4.41 ± 0.04	-9.19 ± 0.00
BAR	-4.75 ± 0.05	-4.44 ± 0.06	-9.19 ± 0.00
Phenol→Cyclohexadienone		$\Delta\Delta G^{\text{abs}}_{\text{solv}}$	
TI		0.01 ± 0.06	
BAR		-0.01 ± 0.04	

Triple junction[a]:
planar

Deleted:
$D_{H_{62}}$-C_6-C_5[a]
$D_{H_{62}}$-C_6-C_5-H_5[a]
$D_{H_{62}}$-C_6-C_5-C_4[a]
$D_{H_{62}}$-C_6-C_1-H_1[a]
$D_{H_{62}}$-C_6-C_1-C_2[a]
D_H-O-C_3-C_4[b]

Modified[a,c]:
$D_{H_{62}}$-C_6-C_1
$D_{H_{62}}$-C_6-H_6

[a] Phenol endpoint;
[b] Cyclohexadienone endpoint;
[c] both to 90° equilibrium angle and 100.0 kcal/rad^2 force constant.

5.3.3 Ethane–Methylamine

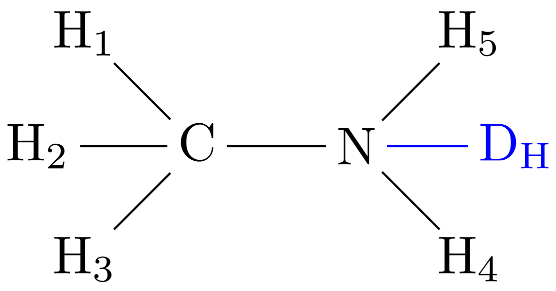

Figure 14: Ethane→Methylamine transformation, Methylamine endpoint

Relative	$\Delta\Delta G_{\mathrm{solv}}$	ΔG_{aq}	ΔG_{gas}
ETH2MTA-1			
TI	-5.90 ± 0.02	10.75 ± 0.02	16.65 ± 0.00
BAR	-5.90 ± 0.01	10.75 ± 0.01	16.65 ± 0.01
ETH2MTA-2[a]			
TI	-5.91 ± 0.02	12.50 ± 0.02	18.41 ± 0.00
BAR	-5.91 ± 0.02	12.50 ± 0.02	18.41 ± 0.00

Absolute	$\Delta G^{\mathrm{abs}}_{\mathrm{solv}}$	$\Delta G^{\mathrm{abs}}_{\mathrm{aq}}$	$\Delta G^{\mathrm{abs}}_{\mathrm{gas}}$
Ethane			
TI	2.25 ± 0.02	-11.11 ± 0.02	-8.86 ± 0.00
BAR	2.25 ± 0.02	-11.11 ± 0.02	-8.86 ± 0.00
Methylamine			
TI	-3.62 ± 0.02	-22.99 ± 0.02	-26.61 ± 0.00
BAR	-3.62 ± 0.02	-22.98 ± 0.02	-26.61 ± 0.00
Ethane → Methylamine		$\Delta\Delta G^{\mathrm{abs}}_{\mathrm{solv}}$	
TI		-5.87 ± 0.02	
BAR		-5.87 ± 0.02	

Triple junction class:
non-planar

Deleted:
D_H-N-C-H_1
D_H-N-C-H_2
D_H-N-C-H_3

Modified:
D_H-N-C[b]
D_H-N-H_4[c]
D_H-N-H_5[c]

[a] naive approach, see introduction;
[b] to 107.691° equilibrium angle and 3.55 kcal/rad^2 force constant;
[c] both to 107.14° equilibrium angle and 3.55 kcal/rad^2 force constant.

5.3.4 Methane–Ammonia

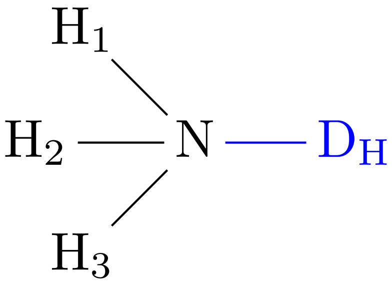

Figure 15: Methane→Ammonia transformation, Ammonia endpoint

Relative	$\Delta\Delta G_{\mathrm{solv}}$	ΔG_{aq}	ΔG_{gas}
MET2AMM-1			
TI	-6.22 ± 0.02	-7.16 ± 0.02	-0.95 ± 0.00
BAR	-6.21 ± 0.01	-7.16 ± 0.01	-0.95 ± 0.01
MET2AMM-2[a]			
TI	-6.05 ± 0.02	-5.46 ± 0.02	0.59 ± 0.00
BAR	-6.05 ± 0.02	-5.46 ± 0.02	0.59 ± 0.01
MET2AMM-3[b]			
TI	-5.60 ± 0.02	-4.83 ± 0.02	0.77 ± 0.00
BAR	-5.61 ± 0.02	-4.83 ± 0.02	0.77 ± 0.01
Absolute	$\Delta G^{\mathrm{abs}}_{\mathrm{solv}}$	$\Delta G^{\mathrm{abs}}_{\mathrm{aq}}$	$\Delta G^{\mathrm{abs}}_{\mathrm{gas}}$
Methane[c]			
TI	2.38 ± 0.02	-2.38 ± 0.02	0.00 ± 0.00
BAR	2.38 ± 0.01	-2.38 ± 0.01	0.00 ± 0.00
Ammonia			
TI	-3.81 ± 0.01	3.81 ± 0.01	0.00 ± 0.00
BAR	-3.82 ± 0.01	3.82 ± 0.01	0.00 ± 0.00
Methane[c] → Ammonia		$\Delta\Delta G^{\mathrm{abs}}_{\mathrm{solv}}$	
TI		-6.19 ± 0.02	
BAR		-6.20 ± 0.01	

Triple junction class:
non-planar

Deleted:
None

Modified:
D_H-N-H_1[d]
D_H-N-H_2[d]
D_H-N-H_3[d]

[a] simulated with angle force constants from Methane, however with equilibrium angles adjusted to 111.749°;
[b] naive approach, see introduction;
[c] with 567 water molecules;
[d] all to 111.749° equilibrium angle and 3.55 kcal/rad^2 force constant.

5.3.5 Ethane–Ammonia

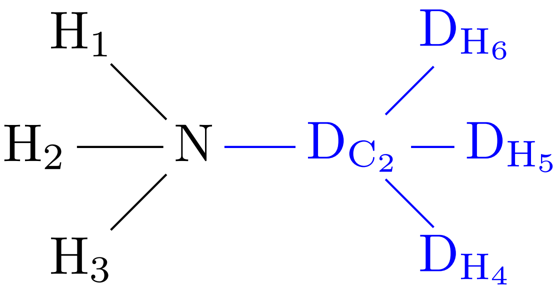

Figure 16: Ethane→Ammonia transformation, Ammonia endpoint

Relative	$\Delta\Delta G_{solv}$	ΔG_{aq}	ΔG_{gas}
ETH2AMM-1			
TI	-6.08 ± 0.02	-16.49 ± 0.02	-10.41 ± 0.00
BAR	-6.08 ± 0.02	-16.49 ± 0.02	-10.41 ± 0.01
ETH2AMM-2[a]			
TI	-5.91 ± 0.02	-14.75 ± 0.02	-8.84 ± 0.00
BAR	-5.91 ± 0.02	-14.75 ± 0.03	-8.84 ± 0.01
ETH2AMM-3[b]			
TI	-5.69 ± 0.02	-14.08 ± 0.02	-8.39 ± 0.00
BAR	-5.69 ± 0.02	-14.08 ± 0.02	-8.39 ± 0.00

Absolute	ΔG^{abs}_{solv}	ΔG^{abs}_{aq}	ΔG^{abs}_{gas}
Ethane			
TI	2.25 ± 0.02	-11.11 ± 0.02	-8.86 ± 0.00
BAR	2.25 ± 0.02	-11.11 ± 0.02	-8.86 ± 0.00
Ammonia			
TI	-3.81 ± 0.01	3.81 ± 0.01	0.00 ± 0.00
BAR	-3.82 ± 0.01	3.82 ± 0.01	0.00 ± 0.00
Ethane → Ammonia		$\Delta\Delta G^{abs}_{solv}$	
TI		-6.06 ± 0.02	
BAR		-6.07 ± 0.02	

Triple junction class:
non-planar

Deleted:
H_2-N-D_{C_2}-D_{H_4}
H_2-N-D_{C_2}-D_{H_5}
H_2-N-D_{C_2}-D_{H_6}
H_3-N-D_{C_2}-D_{H_4}
H_3-N-D_{C_2}-D_{H_5}
H_3-N-D_{C_2}-D_{H_6}

Modified:
H_1-N-D_{C_2} [c]
H_2-N-D_{C_2} [c]
H_3-N-D_{C_2} [c]

[a] simulated with angle force constants from Ethane, however with equilibrium angles adjusted to 111.749°;
[b] naive approach, see introduction;
[c] all to 111.749° equilibrium angle and 3.55 kcal/rad^2 force constant.

5.4 Dual Topology junctions

5.4.1 Acetone–2-Propenol dual topology

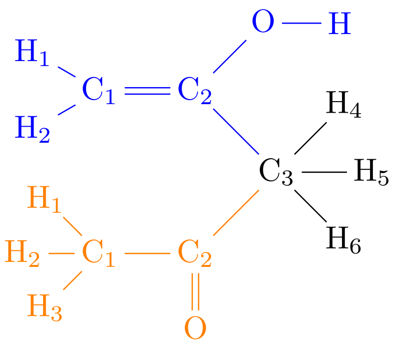

Figure 17: Acetone→2-Propenol transformation with dual topology, both endpoints

Relative	$\Delta\Delta G_{\mathrm{solv}}$	ΔG_{aq}	ΔG_{gas}
ACE2PEOL-2			
TI	1.02 ± 0.02	-23.00 ± 0.02	-24.01 ± 0.01
BAR	1.02 ± 0.02	-22.99 ± 0.03	-24.01 ± 0.01
ACE2PEOL-3[a]			
TI	1.05 ± 0.02	-22.77 ± 0.02	-23.82 ± 0.00
BAR	1.05 ± 0.02	-22.77 ± 0.02	-23.82 ± 0.00

Absolute	$\Delta G^{\mathrm{abs}}_{\mathrm{solv}}$	$\Delta G^{\mathrm{abs}}_{\mathrm{aq}}$	$\Delta G^{\mathrm{abs}}_{\mathrm{gas}}$
Acetone			
TI	-4.28 ± 0.02	40.90 ± 0.02	36.62 ± 0.00
BAR	-4.27 ± 0.02	40.89 ± 0.02	36.61 ± 0.00
2-Propenol			
TI	-3.23 ± 0.02	63.66 ± 0.02	60.43 ± 0.00
BAR	-3.23 ± 0.02	63.66 ± 0.02	60.43 ± 0.00
Acetone → 2-Propenol		$\Delta\Delta G^{\mathrm{abs}}_{\mathrm{solv}}$	
TI		1.05 ± 0.03	
BAR		1.04 ± 0.02	

Triple junction:
non-planar

Deleted:
None[b]

Modified:
H_4-C_3-C_2[c]
H_5-C_3-C_2[c]
H_6-C_3-C_2[c]
H_4-C_3-C_2[d]
H_5-C_3-C_2[d]
H_6-C_3-C_2[d]

[a] naive approach, see introduction;
[b] none in addition to what is anyway deleted in dual topology, i. e. any (bonded) terms spanning between the branches of the endstates (blue and orange);
[c] Acetone endpoint, all to 109.54° equilibrium angle and 3.55 kcal/rad^2 force constant;
[d] 2-Propenol endpoint, all to 111.23° equilibrium angle and 3.55 kcal/rad^2 force constant.

6 Comparison of absolute solvation free energies from simulation with experiment

Absolute solvation free energies for small molecules calculated with the cgenff force field family[7–9] are not readily available in the literature; therefore, we list them here for our model compounds. We compare these to the the experimental values compiled in the Minnesota Solvation Database,[18] as well as to recently reported computational results.[19,20] For the four systems for which a comparison to Ref. 19 is possible, the agreement is excellent. By contrast, the numbers reported by Huenenberger and co-workers[20] differ systematically. However, one should keep in mind the following differences in simulation setup. Calculations were carried out with GROMACS rather than CHARMM. Lennard-Jones interactions were truncated with a force switching function rather than switching the potential energy used in this work and in Ref. 19. A long-range correction for Lennard-Jones interactions was applied, which was not done here since our primary focus was closing the thermodynamic cycle (Fig. 1b of the main manuscript).

Table 1: Absolute aqueous solvation free energies for compounds used in this work

solute	$\Delta G^{\text{abs}}_{\text{solv}}$(this work) d	$\Delta G^{\text{abs}}_{\text{solv}}$ experiment[d]	$\Delta G^{\text{abs}}_{\text{solv}}$(Ref. 19)	$\Delta G^{\text{abs}}_{\text{solv}}$(Ref. 20)
Hexane	2.82 ± 0.06	2.49	2.77	2.42
Propane	2.33 ± 0.03	1.96		
Toluene[a]	-0.10 ± 0.05	-0.89		-0.61
Toluene[a]	-0.08 ± 0.03	-0.89		-0.61
Methane[b]	2.40 ± 0.02	2.00		
Methane[b]	2.38 ± 0.02	2.00		
Ethane	2.25 ± 0.02	1.83	2.23	
Methanol	-4.69 ± 0.02	-5.11	-4.68	-4.60
Water	-6.89 ± 0.01	-6.91	-6.91	-6.61
Water[c]	-6.88 ± 0.01	-6.91	-6.91	-6.61
Pyridine	-4.75 ± 0.03	-4.70		-5.00
Propenol	-4.28 ± 0.05	-4.83		
Dimethylether	-1.02 ± 0.02	-1.92		
Phenol	-4.78 ± 0.04	-6.62	-4.72	
Cyclohexadienone	-4.77 ± 0.04			
Methylamine	-3.62 ± 0.02	-4.56		
Ammonia	-3.81 ± 0.01	-4.29		
Acetone	-4.28 ± 0.02	-3.85		-4.52
2-Propenol	-3.23 ± 0.02			

[a]duplicate ensemble of five simulations with a different number of water molecules; [b]duplicate ensemble of five simulations with the same number of water molecules; [c]instead of the 1 fs integration step used in all other simulations, due to the small physical molecule size, a 0.25 fs integrator step was used here; [d]experimental values from Minnesota Solvation Database.[18]

7 Integrand $\langle \frac{\partial U}{\partial \lambda} \rangle$ for PRP2DIM-2/3

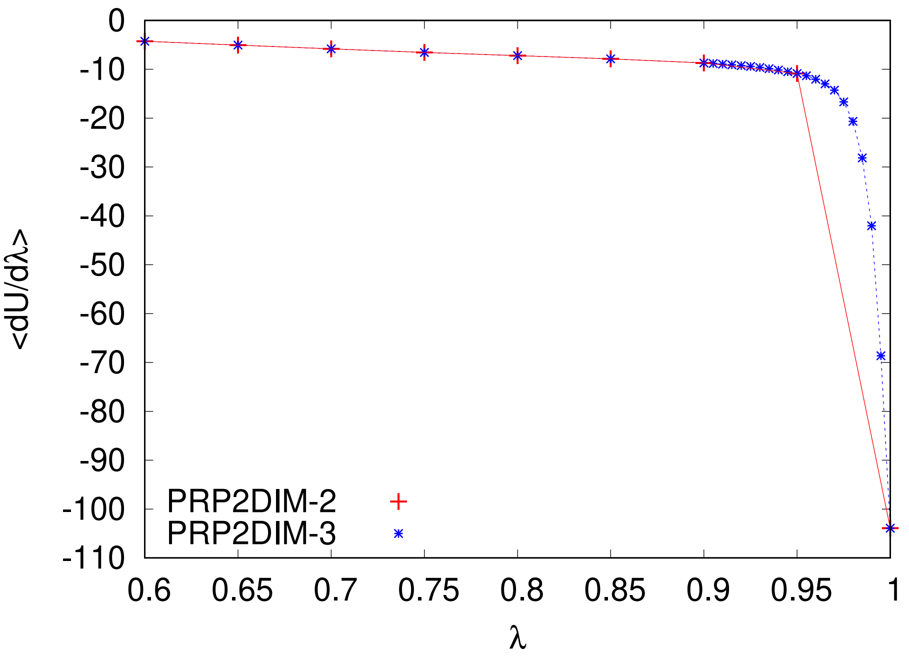

Figure 18: Plot of the integrand $\langle \frac{\partial U}{\partial \lambda} \rangle_\lambda$ (in kcal/mol) as a function of λ ($0.6 \leq \lambda \leq 1$) when using the usual 21 intermediate states (PRP2DIM-2) vs. a finer spacing near $\lambda = 1$ (PRP2DIM-3). For the reasons discussed in the main text, the integrand varies rapidly near $\lambda = 1$, which leads to a systematic error during numerical quadrature. The plot is shown for the gas phase, but the behavior is almost identical in aqueous solution, leading to fortuitous error cancellation for PRP2DIM-2 using TI for $\Delta\Delta A_{solv}$; cf. the main manuscript.

References

(1) Herschbach, D. R.; Johnston, H. S.; Rapp, D. Molecular Partition Functions in Terms of Local Properties. *J. Chem. Phys.* **1959**, *31*, 1652–1661.

(2) Gō, N.; Scheraga, H. A. On the Use of Classical Statistical Mechanics in the Treatment of Polymer Chain Conformation. *Macromolecules* **1976**, *9*, 535–542.

(3) Zhou, H.-X.; Gilson, M. K. Theory of Free Energy and Entropy in Noncovalent Binding. *Chem. Rev.* **2009**, *109*, 4092–4107.

(4) Potter, M. J.; Gilson, M. K. Coordinate Systems and the Calculation of Molecular Properties. *J. Phys. Chem. A* **2002**, *106*, 563–566.

(5) Ryckaert, J.-P.; Ciccotti, G.; Berendsen, H. J. Numerical integration of the cartesian equations of motion of a system with constraints: molecular dynamics of n-alkanes. *J. Comput. Phys.* **1977**, *23*, 327–341.

(6) Andersen, H. C. Rattle: A "velocity" version of the shake algorithm for molecular dynamics calculations. *J. Comput. Phys.* **1983**, *52*, 24–34.

(7) Vanommeslaeghe, K.; Hatcher, E.; Acharya, C.; Kundu, S.; Zhong, S.; Shim, J.; Darian, E.; Guvench, O.; Lopes, P.; Vorobyov, I.; Mackerell, A. D. CHARMM general force field: A force field for drug-like molecules compatible with the CHARMM all-atom additive biological force fields. *J. Comput. Chem.* **2010**, *31*, 671–690.

(8) Vanommeslaeghe, K.; A. D. MacKerell, J. Automation of the CHARMM General Force Field (CGenFF) I: Bond perception and atom typing. *J. Chem. Inf. Mod.* **2012**, *52*, 3144–3154.

(9) Vanommeslaeghe, K.; Raman, E. P.; A. D. MacKerell, J. Automation of the CHARMM General Force Field (CGenFF) II: Assignment of bonded parameters and partial atomic charges. *J. Chem. Inf. Model.* **2012**, *52*, 3155–3168.

(10) Kirkwood, J. G. Statistical Mechanics of Fluid Mixtures. *J. Chem. Phys.* **1935**, *3*, 300–313.

(11) Straatsma, T. P.; McCammon, J. A. Multiconfiguration thermodynamic integration. *J. Chem. Phys.* **1991**, *95*, 1175–1188.

(12) Bennett, C. H. Efficient estimation of free energy differences from Monte Carlo data. *J. Comput. Phys.* **1976**, *22*, 245–268.

(13) Paliwal, H.; Shirts, M. R. A Benchmark Test Set for Alchemical Free Energy Transformations and Its Use to Quantify Error in Common Free Energy Methods. *J. Chem. Theory Comput.* **2011**, *7*, 4115–4134.

(14) Bruckner, S.; Boresch, S. Efficiency of alchemical free energy simulations. II. Improvements for thermodynamic integration. *J. Comput. Chem.* **2011**, *32*, 1320–1333.

(15) Klimovich, P. V.; Shirts, M. R.; Mobley, D. L. Guidelines for the analysis of free energy calculations. *J. Comput. Aided Mol. Des.* **2015**, *29*, 397–411.

(16) Dierckx, P. *Curve and surface fitting with splines*; Oxford University Press: Oxford, 1995.

(17) Fleck, M.; Wieder, M.; Boresch, S. Suppport scripts for ”Dummy atoms in alchemical free energy calculations”. 2020; `https://doi.org/10.5281/zenodo.4381708`.

(18) Marenich, A. V.; Kelly, C. P.; Thompson, J. D.; Hawkins, G. D.; Chambers, C. C.; Giesen, D. J.; Winget, P.; Cramer, C. J.; Truhlar, D. G. Minnesota Solvation Database (MNSOL) version 2012. 2020.

(19) König, G.; Pickard, F.; Huang, J.; Thiel, W.; MacKerell, A.; Brooks, B.; York, D. A Comparison of QM/MM Simulations with and without the Drude Oscillator Model Based on Hydration Free Energies of Simple Solutes. *Molecules* **2018**, *23*, 2695.

(20) Kashefolgheta, S.; Oliveira, M. P.; Rieder, S. R.; Horta, B. A. C.; Acree, W. E.; Hünenberger, P. H. Evaluating Classical Force Fields against Experimental Cross-Solvation Free Energies. *J. Chem. Theory Comput.* **2020**, *16*, 7556–7580.
